# Global burden of multiple sclerosis and its attributable risk factors, 1990–2019

**DOI:** 10.3389/fneur.2024.1448377

**Published:** 2024-10-25

**Authors:** Saeid Safiri, Amir Ghaffari Jolfayi, Seyed Ehsan Mousavi, Seyed Aria Nejadghaderi, Mark J. M. Sullman, Ali-Asghar Kolahi

**Affiliations:** ^1^Neurosciences Research Center, Aging Research Institute, Tabriz University of Medical Sciences, Tabriz, Iran; ^2^Clinical Research Development Unit of Tabriz Valiasr Hospital, Tabriz University of Medical Sciences, Tabriz, Iran; ^3^Social Determinants of Health Research Center, Tabriz University of Medical Sciences, Tabriz, Iran; ^4^HIV/STI Surveillance Research Center, and WHO Collaborating Center for HIV Surveillance, Institute for Futures Studies in Health, Kerman University of Medical Sciences, Kerman, Iran; ^5^Systematic Review and Meta-analysis Expert Group (SRMEG), Universal Scientific Education and Research Network (USERN), Tehran, Iran; ^6^Department of Life and Health Sciences, University of Nicosia, Nicosia, Cyprus; ^7^Department of Social Sciences, University of Nicosia, Nicosia, Cyprus; ^8^Social Determinants of Health Research Center, Shahid Beheshti University of Medical Sciences, Tehran, Iran

**Keywords:** multiple sclerosis, death, prevalence, disability adjusted life years, global

## Abstract

**Background:**

Multiple sclerosis (MS) is a progressively debilitating disorder that has seen a notable rise in prevalence in recent years. This study examines the burden of MS from 1990 to 2019, providing a detailed analysis by age, sex, and sociodemographic index (SDI) across 204 countries and territories.

**Methods:**

Data on the prevalence, death and disability-adjusted life years (DALYs) attributable to MS were obtained from the publically available Global Burden of Disease 2019 project. The estimates are reported as numbers, percentages, and age-standardized rates per 100,000, accompanied by 95% uncertainty intervals.

**Results:**

In 2019, MS accounted for 1.8 million prevalent cases, 22.4 thousand deaths and 1.2 million DALYs worldwide. There were significant declines in the global age-standardized prevalence, mortality and DALY rates of MS over the period 1990–2019. In 2019, females exhibited a higher global point prevalence and a greater total number of prevalent MS cases than males across all age groups. At the regional level, a non-linear relationship was observed between the age-standardized DALY rate of MS and SDI.

**Conclusion:**

Although the global age-standardized DALY rate of MS decreased between 1990 and 2019, MS continues to account for a considerable number of DALYs and prevalent cases. Integrating MS and its associated risk factors into healthcare planning is vital, especially in areas with high levels of socioeconomic development.

## Introduction

Multiple sclerosis (MS) is a chronic autoimmune disease that severely impacts the central nervous system (CNS) and is marked by demyelination, inflammation, and neuronal loss. This condition leads to a variety of neurological symptoms, including vision problems, numbness, weakness, and cognitive difficulties (1). MS also presents a range of “invisible” symptoms that significantly contribute to the overall disease burden. Common invisible symptoms, such as fatigue, cognitive impairment, and deficits in memory and attention, can severely impact daily functioning and quality of life. Additionally, symptoms like dysphagia, urinary incontinence, and sexual dysfunction are prevalent, further diminishing patients’ well-being. Even though these symptoms may not be outwardly apparent, they profoundly affect the prognosis and daily well-being of people living with MS (2–5).

MS is a disabling disease that is affected by an interplay of genetic, environmental, and lifestyle risk factors, including vitamin D deficiency and smoking (6). In recent years, the worldwide prevalence of MS has increased notably, highlighting the growing impact of this condition on global health (7). The symptoms of MS vary depending on the location of the neuronal injury. These may manifest as optic neuritis (e.g., visual blurring, color blindness and reduced visual acuity), myelitis (e.g., sphincter or sexual dysfunction and paraesthesia), brainstem/cerebellar syndrome (e.g., dysphagia, hearing loss and nausea), and cerebral hemispheric syndrome (e.g., hemiparesis and hemisensory deficits) (8). Individuals with MS have a heightened risk of suicide, cardiovascular and respiratory disorders, as well as mortality (9). Beyond its health implications, MS imposes a substantial economic burden, with costs estimated to range between US$463 and US$58616 in low- and middle-income countries. This financial strain primarily arises from the need for caregiver support and the loss of productivity associated with the disease (10, 11).

In 2016, MS ranked 14th among neurological disorders in terms of the age-standardized disability adjusted life years (DALY) rate (12). Furthermore, that year the age-standardized point prevalence and DALY rate of MS were 30 and 16 per 100,000 population worldwide, respectively (12). Between 1990 and 2016, the biggest rises in the age-standardized point prevalence of MS were seen in the East Asia region (44.8%) and Canada (81.9%) (13). Moreover, in 2016 the largest age-standardized point prevalence of MS was seen in High-income North America (164.6 per 100,000 population) (13). Its burden is more pronounced among the middle-aged population and tends to rise with improvements in socioeconomic status (13).

Previous studies have detailed the burden of neurological disorders across different regions and countries utilizing Global Burden of Disease (GBD) data (14–17). Furthermore, the specific burden of MS in China has also been reported utilizing GBD 2019 data (18). In addition, another publication presented the global burden of MS utilizing GBD 2019 data (19). Nevertheless, this research has several limitations, such as not reporting the prevalence of MS, not reporting the burden attributed to different risk factors, missing sex specific indicators at the regional level, not providing this information by age group, the omission of informative maps, not reporting the regional relationship between the burden of MS and socio-economic level, not reporting changes between the years 1990 and 2019, and not reporting the specific numbers for the different regions and countries (19). Much of the information omitted in the previous research is essential for health policymaking. Therefore, our aim was to detail the prevalence, incidence and DALYs that were due to MS by sex, age, and sociodemographic index (SDI) in 204 countries and territories, spanning the years 1990–2019.

## Methods

### Overview

GBD 2019 systematically examined 369 diseases, injuries, and risk factors across 204 nations and territories, 7 super-regions, and 21 regions from 1990 to 2019. The main differences from previous years and methodology of GBD 2019 have been extensively covered elsewhere (20–22). Further specifics concerning the fatal and non-fatal estimates can be obtained from the Global Health Data Exchange.

### Case definition

MS is a chronic and progressive disease characterized by immune-mediated inflammation and demyelination within the CNS. In GBD 2019, the diagnosis of MS adhered to the McDonald criteria, Poser criteria, Schumacher criteria, and McAllen criteria, as well as clinical neurological examinations (21). All conditions coded as G35-G35.9 and 340–340.9, as per the International Classification of Diseases version 10 (ICD10) and ICD9, respectively, were classified as MS (21).

### Fatal estimation

The Cause of Death Ensemble Modeling (CODEm) approach was employed to calculate MS-related mortality for both sexes, covering an age range of 5 to 95+ years old (21). Supplementary Table S1 displays the covariates included in the modeling process. Unadjusted mortality estimates were refined with CoDCorrect to yield the years of life lost (YLLs). MS was modeled using data from the cause of death database, specifically utilizing vital registration and surveillance data. The study identified outliers by excluding data points that showed implausible values, notable deviations from established age or temporal trends, or significant discrepancies compared to data from the same location or similar locations with comparable characteristics, such as SDI (21). In cases where inconsistencies between various coding systems for a given location over time could not be resolved through data processing, a decision was made by GBD team to consider one system as reliable rather than excluding the other (21).

### Non-fatal estimation

Estimates for MS were calculated using two main types of data sources. The first type comprised studies gathered through systematic literature reviews of representative population-based observational studies, which were last updated in GBD 2017 (21, 23). Studies were excluded if they lacked a clearly defined sample or relied on specific clinics or patient organizations (23). The second type comprised claims data which were obtained and analyzed by the GBD Clinical informatics team. GBD 2019 incorporated new claims data from Poland and extended the coverage of claims data from the U.S.A. to encompass the years 2015–2016 (21). The mentioned data links encompassed all instances of inpatient and outpatient encounters tied to an individual, providing both primary and secondary diagnoses for each encounter (21). The process of identifying a prevalent case from claims data involved selecting individuals with at least one inpatient encounter or two or more outpatient encounters (21).

In cases where epidemiological data encompassed prevalence or incidence measures stratified by age for both sexes together, as well as for all ages combined by sex, we derived the sex ratio of cases from the study and used it to estimate age-sex-specific data for both sexes (21). In order to derive sex-specific metrics from studies that solely presented data for the sexes together, GBD employed a log sex ratio model in MR-BRT (21). This involved incorporating all sex-specific measurements from other studies into the database and merging them with the GBD sex-specific population estimates for the relevant age group (21). The data from the United States claims in 2000 pertains to a limited sub-group of individuals with commercial insurance. To address this, a pre-modeling bias adjustment was conducted by GBD using MR-BRT Crosswalk Adjustment for the aforementioned United States claims data. For MS estimation, DisMod-MR 2.1 was employed by GBD along with the following covariates: the healthcare access and quality index for excess mortality rate, and the absolute value of average latitude for the prevalence and incidence. The prior settings encompassed no remission across all ages, zero incidence or excess mortality for individuals younger than 5, and an incidence rate restricted to less than 0.000005 for ages above 60 (21).

### Severity and years lived with disability

The lay description and disability weights (DWs) of MS were defined according to Kurtzke’s Expanded Disability Status Scale (EDSS) (21). The disability levels and DWs associated with MS were categorized as follows: EDSS score of zero classified as asymptomatic with a DW of zero, an EDSS score of 0 to ≤3.5 was considered mild with a DW of 0.183 (0.124–0.253), an EDSS score of 3.5 to ≤6.5 was classified as moderate with a DW of 0.463 (0.313–0.613), and an EDSS score of 6.5 < to ≤9.5 was categorized as severe with a DW of 0.719 (0.534–0.858) (21). To address the lack of data on the number of cases with an EDSS score of zero in certain sources, GBD utilized a two-step meta-analysis approach (21). Initially, studies solely reporting the mild category were categorized to include only those providing information on the number of cases with EDSS scores of 0. Subsequently, meta-analyses were conducted on the percentage of asymptomatic and mild cases. Following this, the complete dataset underwent meta-analyses to establish the proportions of mild, moderate, and severe cases (21).

### Compilation of results

The process for calculating the YLLs involved multiplying the number of deaths in each age range by the residual life expectancy for that particular age range, using the GBD standard life table. The calculation of DALYs involved adding the Years Lived with Disability (YLDs) and YLLs together. The estimates were provided as numbers, proportions, and age-standardized rates per 100,000, each accompanied by 95% uncertainty intervals (UIs). The distribution of uncertainty was achieved by running 1,000 iterations at every computational step. This approach enabled the integration of uncertainty from various causes, such as estimates of residual non-sampling error, corrections of measurement error, and input data. The process for defining UIs involved identifying the 25th and 975th values from the ordered iterations. In our study, we employed Smoothing Splines models (22) to investigate the correlation between the SDI and the burden of MS, specifically in relation to the MS attributable DALYs.

### Risk factors

The percentage of MS-related deaths and DALYs due to smoking were estimated. To determine the prevalence of current and former smokers, cross-sectional nationally representative household surveys were utilized (20). GBD 2019 employed a Bayesian meta-regression model to generate nonlinear dose–response curves by synthesizing effect sizes derived from cohort and case–control studies (20). The exposure definition for relative risk was based on cigarettes per smoker per day, which was used to estimate the Population Attributable Fraction (PAF). Further details about the definition of this risk factor and its relative risk for MS can be found in another source (20).

## Results

### Global level

In 2019, there were 1.8 million (95% UI: 1.5 to 2.0) prevalent cases of MS, with an age-standardized point prevalence of 21.3 (95% UI: 18.5 to 23.9) per 100,000, representing a 6.2% (95% UI: −8.7 to −3.8) decline from 1990. That year, MS was responsible for 22.4 thousand deaths (95% UI: 20.2 to 27.8), with an age-standardized death rate of 0.3 (95% UI: 0.2 to 0.3), reflecting a 14% reduction (95% UI: −29.1 to −5.9) over the measurement period. Additionally, MS accounted for 1.2 million DALYs (95% UI: 1.0 to 1.4), with an age-standardized rate of 14.0 (95% UI: 12.0 to 16.6). Over the reporting period, DALYs decrease by 13.2% (95% UI: −20.9 to −6.3) (Table 1).

**Table 1:** Prevalent cases, deaths and DALYs due to multiple sclerosis in 2019 and percentage change of age-standardized rates (ASRs) per 100,000, by GBD region, from 1990 to 2019 (Generated from data available from: http://ghdx.healthdata.org/gbd-results-tool).

	Prevalence (95% UI)			Deaths (95% UI)			DALYs (95% UI)		
	No (95% UI)	ASRs per 100,000 (95% UI)	Percentage change in ASRs between 1990 and 2019	No (95% UI)	ASRs per 100,000 (95% UI)	Percentage change in ASRs between 1990 and 2019	No (95% UI)	ASRs per 100,000 (95% UI)	Percentage change in ASRs between 1990 and 2019
Global	1,756,792 (1,531,919, 1,973,623)	21.3 (18.5, 23.9)	−6.2 (−8.7, −3.8)	22,439 (20,226, 27,792)	0.3 (0.2, 0.3)	−14 (−29.1, −5.9)	1,159,832 (1,001,180, 1,381,870)	14 (12, 16.6)	−13.2 (−20.9, −6.3)
High-income Asia Pacific	24,006 (19,021, 29,147)	8.6 (6.8, 10.5)	11.4 (9.6, 13.1)	321 (266, 498)	0.1 (0.1, 0.1)	−18.3 (−37.6, 2.5)	15,516 (12,324, 20,968)	5.6 (4.4, 7.7)	−8.9 (−23.3, 4.7)
High-income North America	476,332 (438,853, 513,566)	103.8 (95.4, 112.1)	5.5 (−4.6, 14.5)	4,632 (3,116, 5,134)	0.8 (0.6, 0.9)	25.1 (−16.2, 44.7)	241,678 (195,635, 278,601)	49.3 (40.2, 57.4)	8.8 (−5.6, 17.8)
Western Europe	522,842 (459,258, 590,605)	88.5 (77.7, 100.7)	27.6 (24.7, 30.6)	5,235 (3,829, 6,538)	0.7 (0.5, 0.9)	−2.8 (−36.2, 9)	271,039 (222,001, 324,346)	43.5 (35.8, 52.7)	7.3 (−13.5, 16)
Australasia	21,100 (18,034, 23,970)	57 (48.4, 65.4)	43.2 (30.8, 55.1)	215 (156, 276)	0.5 (0.4, 0.6)	2.6 (−36.4, 19.5)	11,116 (9,024, 13,401)	28.9 (23.3, 35.3)	17.9 (−10.5, 32.9)
Andean Latin America	4,177 (3,242, 5,078)	6.8 (5.3, 8.3)	32.9 (28, 38.3)	79 (59, 101)	0.1 (0.1, 0.2)	2.9 (−28, 37.6)	3,735 (2,952, 4,623)	6.1 (4.9, 7.6)	7.2 (−17, 34)
Tropical Latin America	47,516 (38,255, 56,927)	18.9 (15.2, 22.6)	16 (13.3, 18.9)	421 (362, 599)	0.2 (0.1, 0.2)	5.9 (−20.9, 25)	25,312 (20,305, 31,971)	10.1 (8.1, 12.7)	10.2 (−8.8, 20.5)
Central Latin America	21,533 (17,150, 25,931)	8.4 (6.7, 10.2)	36.2 (32.9, 40.5)	533 (414, 683)	0.2 (0.2, 0.3)	40.5 (−7.2, 80.9)	24,524 (20,015, 29,814)	9.6 (7.9, 11.7)	38.5 (0.1, 68.1)
Southern Latin America	17,804 (14,604, 21,088)	23.6 (19.3, 28.1)	5.3 (2.3, 8.4)	185 (150, 315)	0.2 (0.2, 0.4)	−16.5 (−28.1, 16.8)	10,639 (8,314, 15,021)	14 (11, 19.8)	−8.5 (−18.8, 12.8)
Caribbean	5,285 (4,204, 6,317)	10.4 (8.3, 12.5)	16.4 (13.8, 19.6)	123 (92, 156)	0.2 (0.2, 0.3)	14.2 (−11.5, 37.5)	5,730 (4,627, 7,078)	11.3 (9.1, 14)	12.6 (−8.1, 30.3)
Central Europe	60,506 (53,805, 67,750)	41 (36.3, 46.2)	8.8 (5.1, 13.3)	1,148 (872, 1845)	0.6 (0.5, 1.1)	−26.7 (−44.1, 18.5)	51,364 (40,325, 75,257)	32.7 (25.6, 48.1)	−21.1 (−35.8, 16.4)
Eastern Europe	57,320 (47,592, 67,240)	22 (18.2, 26)	−15 (−16.9, −13.3)	1,421 (942, 2,848)	0.5 (0.3, 1)	−14.7 (−37.5, 29.6)	69,170 (48,217, 125,154)	26.2 (18.3, 47.6)	−14.2 (−32.9, 23.7)
Central Asia	28,520 (24,097, 32,799)	32.1 (27.4, 36.9)	−2 (−5.4, 1.8)	146 (117, 223)	0.2 (0.2, 0.3)	−5.9 (−21.9, 31.8)	12,221 (9,341, 15,781)	13.8 (10.7, 17.6)	−3.9 (−13.2, 10.8)
North Africa and Middle East	222,696 (190,733, 256,781)	39 (33.6, 44.7)	11.5 (10, 12.8)	1,436 (1,176, 1814)	0.3 (0.2, 0.3)	5.1 (−25, 47.6)	115,886 (93,053, 144,758)	19.9 (16.1, 24.7)	6.2 (−11.6, 30.5)
South Asia	136,608 (107,613, 167,811)	8 (6.3, 9.7)	14.3 (13, 15.8)	2,915 (2,387, 3,672)	0.2 (0.2, 0.2)	8.2 (−18.3, 64.4)	144,077 (119,712, 177,476)	8.6 (7.1, 10.5)	10.3 (−11.7, 47.5)
Southeast Asia	18,584 (14,181, 23,400)	2.6 (2, 3.2)	5.7 (4.2, 7.1)	771 (593, 1,106)	0.1 (0.1, 0.2)	−6 (−26.5, 30.4)	32,510 (25,887, 44,139)	4.5 (3.6, 6.1)	−10.9 (−30, 21.7)
East Asia	45,850 (35,804, 57,092)	2.4 (1.9, 3)	25.4 (22.8, 28.1)	1888 (1,527, 2,506)	0.1 (0.1, 0.1)	−27.5 (−46.1, 10.7)	75,175 (62,164, 95,960)	3.8 (3.1, 4.8)	−24.4 (−41.9, 11.1)
Oceania	201 (150, 258)	1.7 (1.3, 2.2)	−1 (−3.7, 2)	8 (6, 12)	0.1 (0.1, 0.1)	−10.2 (−28.3, 16.8)	363 (264, 502)	3.5 (2.5, 4.8)	−9.6 (−26.7, 14)
Western Sub-Saharan Africa	23,509 (18,611, 28,685)	7.8 (6.3, 9.2)	19.4 (16.7, 22.8)	559 (439, 740)	0.2 (0.2, 0.3)	28.4 (−7.3, 86.2)	28,344 (22,716, 35,170)	9.9 (8, 12.5)	28 (−4.3, 70)
Eastern Sub-Saharan Africa	13,071 (9,911, 16,699)	4.7 (3.7, 5.9)	4.8 (3.7, 6.1)	237 (136, 348)	0.1 (0.1, 0.2)	1.4 (−28.1, 37.1)	12,717 (8,611, 17,089)	5.1 (3.4, 6.9)	1 (−25, 27.6)
Central Sub-Saharan Africa	3,665 (2,759, 4,617)	4.1 (3.1, 5.1)	6.4 (3.4, 9.6)	79 (50, 123)	0.1 (0.1, 0.2)	4 (−23.8, 44.3)	4,031 (2,878, 5,787)	4.9 (3.5, 7.1)	4.4 (−20.8, 36.6)
Southern Sub-Saharan Africa	5,667 (4,417, 7,047)	7.8 (6.1, 9.5)	5 (3.4, 6.4)	87 (71, 104)	0.1 (0.1, 0.2)	4.2 (−9.7, 21.6)	4,686 (3,927, 5,553)	6.6 (5.6, 7.8)	1.4 (−9.7, 12.9)

### Regional level

In 2019, the regions with the highest age-standardized point prevalence of MS were High-income North America [103.8 (95% UI: 95.4 to 112.1)], Western Europe [88.5 (95% UI: 77.7 to 100.7)] and Australasia [57.0 (95% UI: 48.4 to 65.4)]. Conversely, the lowest age-standardized point prevalence rates were observed in Oceania [1.7 (95% UI: 1.3 to 2.2)], East Asia [2.4 (95% UI: 1.9 to 3.0)] and Southeast Asia [2.6 (95% UI: 2.0 to 3.2)] (Table 1). Supplementary Figure S1 presents the sex-specific estimates for the age-standardized point prevalence of MS in 2019 at the regional-level, while Supplementary Figure S2 illustrates the estimates for the number of prevalent cases of MS over the period 1990–2019.

In 2019, the highest age-standardized death rates due to MS were observed in High-income North America [0.8 (95% UI: 0.6 to 0.9)], Western Europe [0.7 (95% UI: 0.5 to 0.9)] and Central Europe [0.6 (95% UI: 0.5 to 1.1)]. Conversely, the lowest rates of MS were found in Oceania [0.1 (95% UI: 0.1 to 0.1)], East Asia [0.1 (95% UI: 0.1 to 0.1)] and High-income Asia Pacific [0.1 (95% UI: 0.1 to 0.1)] (Table 1). Supplementary Figure S3 presents the sex-specific estimates for the age-standardized death rate of MS in 2019 at the regional-level, while Supplementary Figure S4 illustrates the estimated number of MS-attributable death cases from 1990 to 2019 at the regional-level.

In 2019, the largest age-standardized DALY rates due to MS were recorded in High-income North America [49.3 (95% UI: 40.2 to 57.4)], Western Europe [43.5 (95% UI: 35.8 to 52.7)] and Central Europe [32.7 (95% UI: 25.6 to 48.1)]. Conversely, Oceania [3.5 (95% UI: 2.5 to 4.8)], East Asia [3.8 (95% UI: 3.1 to 4.8)] and Southeast Asia [4.5 (95% UI: 3.6 to 6.1)] had the lowest (Table 1). Supplementary Figure S5 depicts the sex-specific estimates for the age-standardized DALY rate of MS in 2019 at the regional level, while Supplementary Figure S6 shows the estimates for the number of DALYs of MS from 1990 to 2019 also at the regional level.

Between 1990 and 2019, the most substantial increases in the age-standardized point prevalence were seen in Australasia [43.2% (95% UI: 30.8 to 55.1)], Central Latin America [36.2% (95% UI: 32.9 to 40.5)], and Andean Latin America [32.9% (95% UI: 28.0 to 38.3)]. In contrast, Eastern Europe [−15.0% (95% UI: −16.9 to −13.3)] was the only region to show a decline (Table 1). Regional-level, sex-specific estimates for the proportional change in age-standardized point prevalence of MS from 1990 to 2019 are presented in Supplementary Figure S7.

The regions showed no significant changes in the age-standardized mortality rates between 1990 and 2019 (Table 1). Supplementary Figure S8 illustrates the sex-specific figures for the proportional change in regional age-standardized mortality rates over this period.

Between 1990 and 2019, Central Latin America [38.5% (95% UI: 0.1 to 68.1)] stood out as the sole GBD region to exhibit a rise in the age-standardized DALY rate of MS, while no regions recorded a decrease during this timeframe (Table 1). Supplementary Figure S9 presents regional-level, sex-specific estimates for the percentage change in age-standardized DALY rates from 1990 to 2019.

### Country level

**Figure 1:**
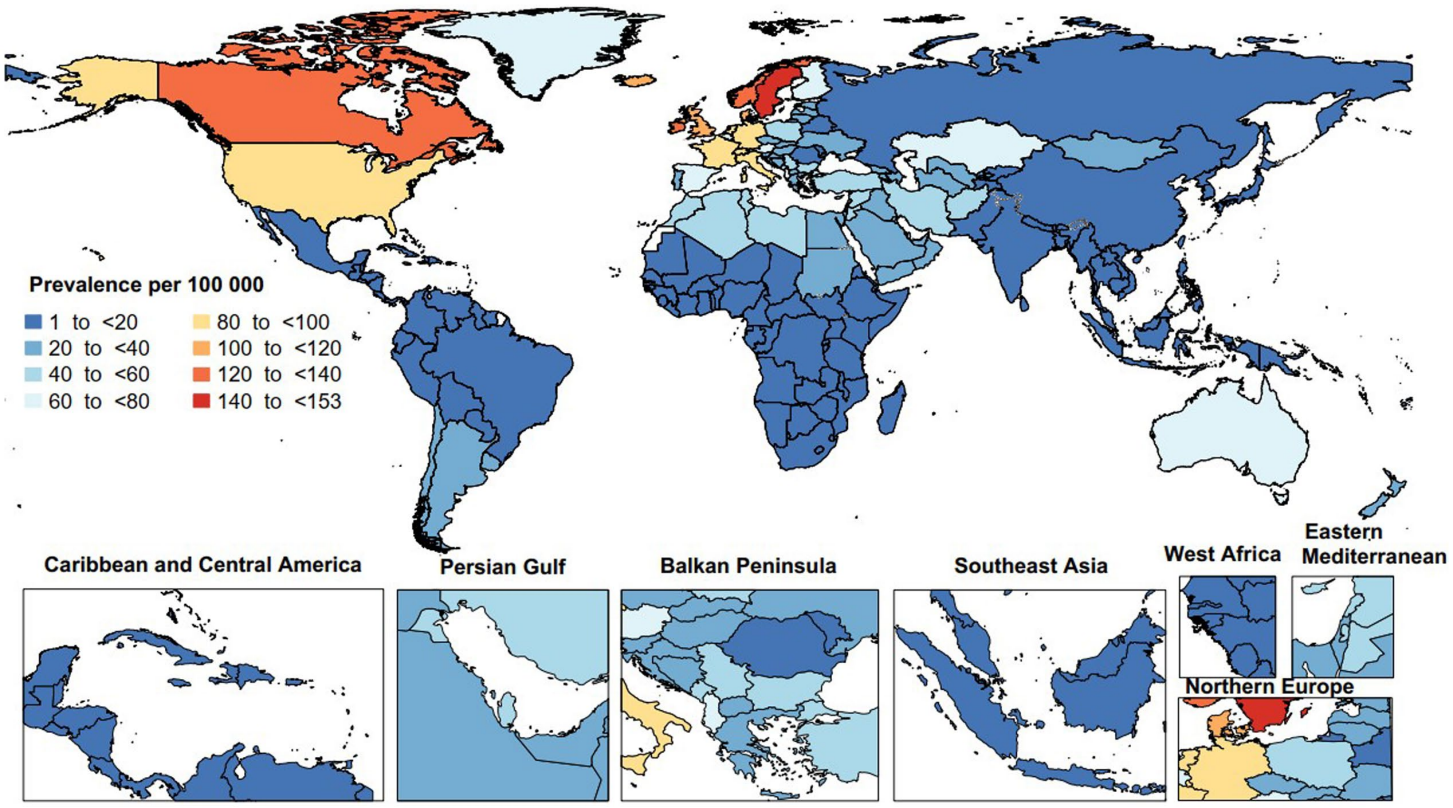
Age-standardized point prevalence of multiple sclerosis per 100,000 population in 2019, by country. (Generated from data available from: http://ghdx.healthdata.org/gbd-results-tool).

In 2019, the age-standardized point prevalence of MS varied from 1.4 to 152.2 per 100,000. Sweden [152.2 (95% UI: 132.1 to 174.0)], Norway [139.2 (95% UI: 117.0 to 163.8)], and Canada [136.8 (95% UI: 133.0 to 140.9)] had the largest age-standardized point prevalence of MS. Conversely, the lowest were seen in Nauru [1.4 (95% UI: 1.1 to 1.8)], Papua New Guinea [1.6 (95% UI: 1.2 to 2.0)], and Kiribati [1.6 (95% UI: 1.2 to 2.0)] (Figure 1).

**Figure 2:**
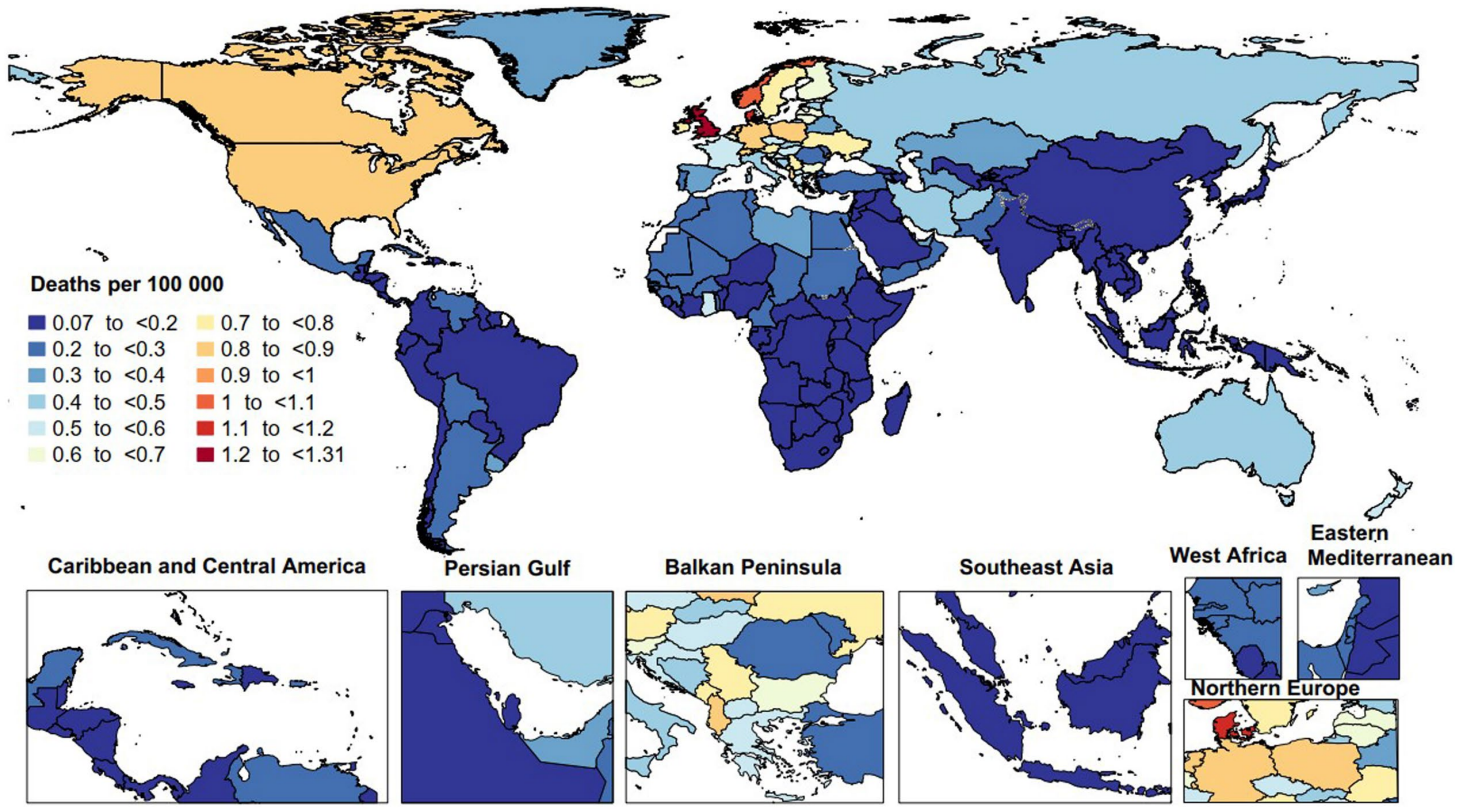
Age-standardized death rate of multiple sclerosis per 100,000 population in 2019, by country. (Generated from data available from: http://ghdx.healthdata.org/gbd-results-tool).

In 2019, the age-standardized mortality rate attributable to MS varied from 0.1 to 1.3 per 100,000 population. The United Kingdom [1.3 (95% UI: 1.0 to 1.6)], Denmark [1.1 (95% UI: 0.7 to 1.3)], and Norway [1.0 (95% UI: 0.5 to 1.2)] had the largest age-standardized death rates due to MS. In contrast, Guam [0.1 (95% UI: 0.1 to 0.1)], Singapore [0.1 (95% UI: 0.0 to 0.1)], and the Maldives [0.1 (95% UI: 0.0 to 0.1)] had the smallest rates in 2019 (Figure 2).

**Figure 3:**
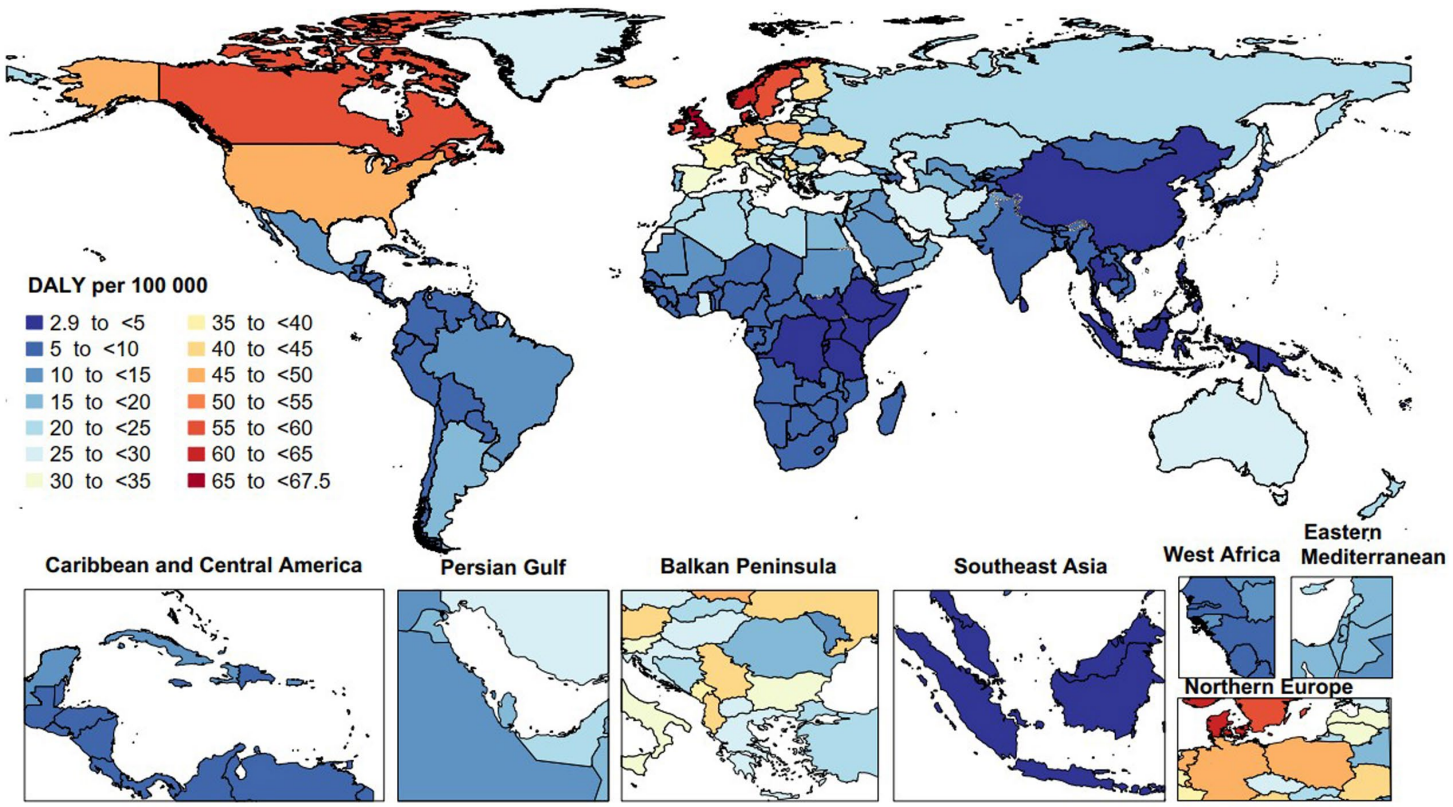
Age-standardized disability-adjusted life years (DALYs) rate of multiple sclerosis per 100,000 population in 2019, by country. (Generated from data available from: http://ghdx.healthdata.org/gbd-results-tool).

In 2019, the age-standardized DALY rates due to MS varied from 2.9 to 67.5 per 100,000. The United Kingdom [67.5 (95% UI: 57.2 to 82.3)], Norway [64.5 (95% UI: 45.5 to 79.4)], and Denmark [61.9 (95% UI: 47.2 to 74.1)] recorded the largest age-standardized DALY rates of MS. Conversely, the Maldives [2.9 (95% UI: 1.7 to 4.8)], Papua New Guinea [3.1 (95% UI: 2.0 to 4.5)], and Guam [3.1 (95% UI: 2.6 to 3.9)] had the lowest (Figure 3).

Between 1990 and 2019, the largest increases in the age-standardized point prevalence of MS were found in Taiwan [169.4% (95% UI: 138.8 to 206.2)], Ghana [62.5% (95% UI: 49.9 to 79.1)], and Kuwait [59.7% (95% UI: 50.5 to 69.9)]. Conversely, the largest declines during this period were seen in Hungary [−22.7% (95% UI: −30.2 to −14.7)], Ukraine [−16.8% (95% UI: −20.6 to −13.2)], and Uzbekistan [−16.2% (95% UI: −21.5 to −10.0)] (Supplementary Table S2).

Between 1990 and 2019, increases in the age-standardized mortality rates attributed to MS were only seen in Kenya [35.6% (95% UI: 6.6 to 92.3)] and Belize [29.2% (95% UI: 2.2 to 80.4)]. In contrast, decreases during this period were only seen in the Cook Islands [−29.2% (95% UI: −47.6 to −4.9)] and the Republic of Korea [−25.0% (95% UI: −55.4 to −0.7)] (Supplementary Table S3).

Increases in the age-standardized DALY rate of MS, from 1990 to 2019, were observed in Mexico [50.2% (95% UI: 3.3 to 91.0)], Greece [48.0% (95% UI: 4.8 to 77.1)] and Kuwait [38.2% (95% UI: 21.1 to 58.7)]. In contrast, only the Cook Islands [−25.2% (95% UI: −44.5 to −0.6)] recorded a decrease (Supplementary Table S4).

### Sex and age patterns

**Figure 4:**
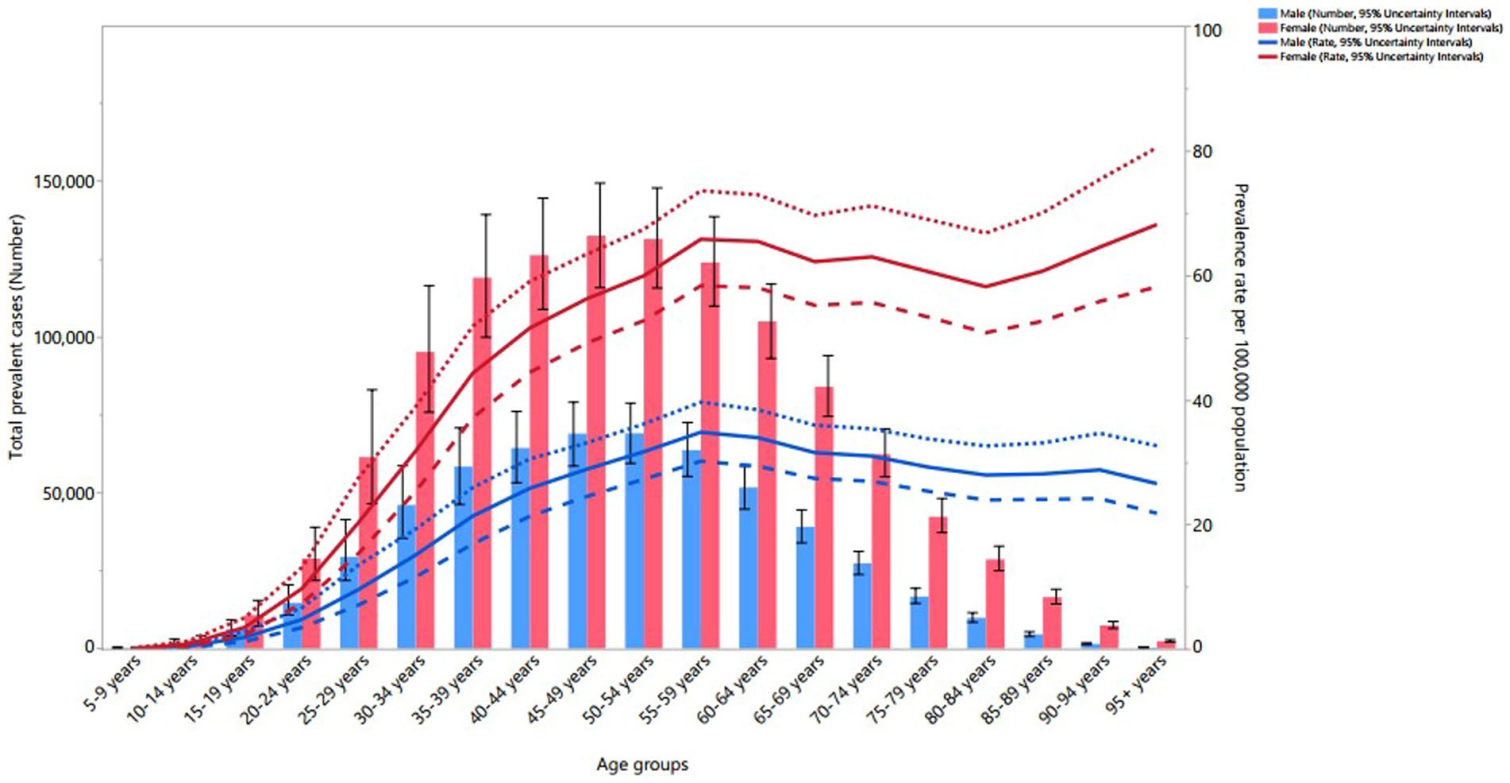
Global number of prevalent cases and age-standardized point prevalence of multiple sclerosis per 100,000 population by age and sex, 2019; dotted and dashed lines indicate 95% upper and lower UIs, respectively. (Generated from data available from: http://ghdx.healthdata.org/gbd-results-tool).

In 2019, the worldwide point prevalence of MS increased for both sexes until reaching the 55–59 age range, after which it stabilized. Globally, the number of prevalent cases in both sexes rose with age, peaking in 45–49 age range before declining to the 95+ age range. Moreover, females exhibited a higher point prevalence and a greater total number of prevalent cases than males across all age ranges, except for 5–19 age range (Figure 4).

In 2019, the worldwide mortality rate due to MS increased with age for both males and females, with the number of deaths peaking in the 55–59 age range before declining to the oldest age range. The mortality rate and the total number of deaths showed no significant sex differences (Supplementary Figure S10). Additionally, in 2019, the global DALY rate attributable to MS for both sexes rose up to the 55–59 age range, while the total DALYs rose until the 50–54 age range, before both decreased to the 95+ age range. Women exhibited higher DALY rates than males in the 20–29 and 85+ age groups, and higher total DALYs in the 20–29 and 65+ age range (Supplementary Figure S11).

### MS burden by SDI

**Figure 5:**
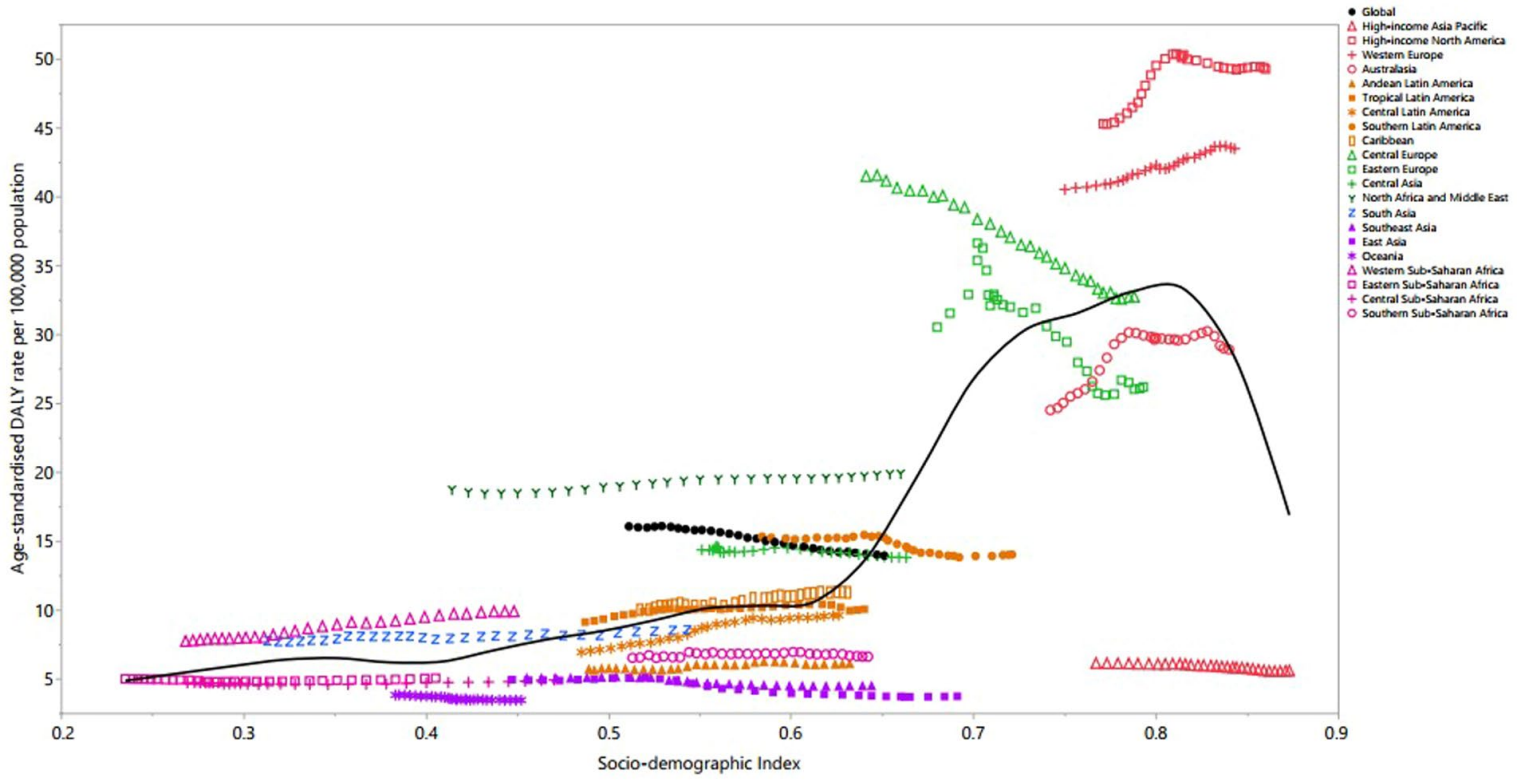
Age-standardized DALY rates of multiple sclerosis per 100,000 population for the 21 Global Burden of Disease regions by sociodemographic index (SDI), 1990–2019; expected values based on SDI and disease rates in all locations are shown as the black line. DALY, disability-adjusted life year. (Generated from data available from: http://ghdx.healthdata.org/gbd-results-tool).

Regionally, a non-linear relationship was observed between the age-standardized DALY rate of MS and the SDI from 1990 to 2019. The burden of MS rose slightly up to an SDI of 0.6, followed by a dramatic increase to an SDI of 0.8, then decreased with further increases in SDI. Regions such as High-income North America, Western Europe, North Africa and Middle East, and Western Sub-Saharan Africa displayed higher than expected DALY rates from 1990 to 2019. Conversely, High-income Asia Pacific, Andean Latin America, Central Latin America, Southeast Asia, East Asia, Oceania, Central Sub-Saharan Africa, and Southern Sub-Saharan Africa exhibited levels that were lower than expected across the measurement period (Figure 5).

In 2019, a clear linear positive association was evident between the age-standardized DALY rates due to MS and the SDIs of the 204 countries and territories. The burden of MS remained stable up to an SDI of 0.6, then dramatically increased to the highest SDI levels. Countries and territories, such as the United Kingdom, Norway, Denmark, Canada, Afghanistan, Albania and Ghana exhibited significantly higher than expected burdens. In contrast, Japan, Singapore, Guam, the Republic of Korea, American Samoa, China and Dominica had burdens that were lower than expected (Supplementary Figure S12).

### Deaths and DALYs due to smoking

The global proportion of MS-related DALYs and deaths that were due to smoking exhibited similar patterns among the different age groups. The global percentage of MS related DALYs that were due to smoking increased up to the 50–54 (24.6%) and 55–59 (12.7%) age ranges in men and women, respectively, then declined with advancing age (Supplementary Figure S13). Likewise, the global percentage of MS related deaths that were attributable to smoking increased up to the 50–54 (24.9%) and 55–59 (12.2%) age ranges in men and woman, respectively, before decreasing with advancing age (Supplementary Figure S14). Additionally, across all age groups, males had a higher percentage of MS-related deaths and DALYs that were due to smoking, compared to females.

The percentage of MS-related deaths and DALYs that were due to smoking were similar across GBD regions. In males, East Asia (32.2%), Eastern Europe (30.9%) and Central Europe (25.6%) had the largest proportion of DALYs due to smoking, while Andean Latin America (8.1%), Western Sub-Saharan Africa (9.0%), and Eastern Sub-Saharan Africa (10.2%) had the lowest (Supplementary Figure S15). For females, Central Europe (17.0%), Western Europe (14.5%), and High-income North America (14.3%) had the largest percentage of DALYs due to smoking, while Western Sub-Saharan Africa (1.1%), Central Sub-Saharan Africa (1.2%), and Eastern Sub-Saharan Africa (1.8%) had the lowest percentages (Supplementary Figure S15).

The percentage of MS-related deaths that were attributed to smoking varied across the GBD regions. In males, East Asia (31.8%), Eastern Europe (30.8%), and Southeast Asia (25.2%) had largest percentage of deaths that were attributable to smoking, while Andean Latin America (8.1%), Western Sub-Saharan Africa (8.9%), and Central Sub-Saharan Africa (10.3%) had the lowest (Supplementary Figure S16). For females, Central Europe (15.3%), High-income North America (13.3%), and Southern Latin America (13.2%) had the largest percentage of deaths attributable to smoking, while Western Sub-Saharan Africa (1.1%), Central Sub-Saharan Africa (1.3%), and Andean Latin America (1.7%) had the smallest percentages (Supplementary Figure S16).

## Discussion

The findings from our research revealed substantial reductions in the global MS rates over the past 30 years. In 2019, the prevalence of MS was consistently higher in women compared to men in all age groups. Additionally, the correlation between MS rates and socioeconomic development exhibited regional variations and did not adhere to a straightforward linear trend. This suggests that the relationship between MS prevalence and socioeconomic factors is complex and influenced by various regional dynamics.

The worldwide age-standardized rate of MS was 21.3 per 100,000 in 2019. Among the different regions, the highest point prevalence was seen in High-income North America, while Oceania had the lowest. According to Walton et al.’s research, the global number of individuals diagnosed with MS reached 2.8 million in 2020, marking a 30% increase since 2013. The worldwide prevalence for that year stood at 35.9 per 100,000 people, and this surge in the prevalence of MS occurred in all global regions (7). Of the 81 countries with data from both years, only 14% indicated a consistent or decreasing prevalence rate (7). Another study reported 2.2 million cases of MS globally in 2016, reflecting a 10.4% rise in the age-standardized point prevalence since 1990 (13). However, our study showed a decline in the age-standardized point prevalence of MS from 1990 to 2019. This difference may be as a result of advancements in the modeling techniques and the sources of data used in GBD 2019 and 2016. These advanced methods may have addressed data inconsistencies or refined the identification and counting of cases, potentially leading to a lower estimate of MS prevalence. However, this conclusion has limitations. The study might have overlooked factors such as regional disparities in healthcare access, under-reporting of MS cases in low-resource settings, and varying rates of misdiagnosis. Additionally, advancements in diagnostic tools do not always result in immediate changes in global data, particularly in countries with limited diagnostic capabilities. This inconsistency between findings and widespread evidence should prompt further investigation into the study’s methodology, potential biases, and limitations in data coverage or quality. Future GBD iterations should evaluate whether this declining trend will persist in the upcoming years, particularly after the coronavirus disease 2019 pandemic.

Our study revealed a higher prevalence of MS in women compared to men, with this difference being particularly pronounced among the elderly population. The increased likelihood of MS in women suggests the influence of sex-related factors, including hormones, genetics, environment influences, and epigenetics mechanisms. Despite the higher incidence rates and stronger immune responses observed in women, their prognosis does not appear to be worse than that of men. This indicates the presence of resilience mechanisms that may help mitigate disease progression (24, 25). Previous research has consistently shown that females are more susceptible to MS than males, with a prevalence ratio of roughly 3:1 (26–28). This pattern underscores the heightened susceptibility of females to several autoimmune conditions. However, women exhibit more robust immune reactions than men, responding more strongly to both endogenous and exogenous antigens, a pattern that has been observed across several species (29). Gender-specific differences can be attributable to sex chromosomes and hormonal influences (24, 29). These differences are reflected in antibody responses, CD4+ lymphocyte counts, cytokine productions and the impact of sexual hormones, which together contribute to the stronger immune responses seen in women, as well as their increased susceptibility to autoimmune diseases (27). Considering the impact of sex on disease prevalence and severity, it is important to implement novel sex-specific perspectives, recommendations, strategies, and therapies in disease prevention and treatment to improve outcomes (30).

Generally, the age-standardized DALY rate of MS tended to rise with increasing SDI levels, a pattern evident at both the regional and national levels. A study investigating the relationship between socioeconomic status and the progression and outcomes of MS, drawing data from cohorts in Canada and Wales, revealed a significant association between higher socioeconomic status and a reduced risk of severe disability progression. Specifically, those with better socioeconomic support had a lower risk of reaching significant disability milestones and transitioning to a more advanced stage of MS. Conversely, MS patients from lower socioeconomic backgrounds had a higher likelihood of experiencing a deterioration in their condition (31). Furthermore, a study involving 1.5 million Danes born between 1966 and 1992, explored the link between childhood socioeconomic status and the risk of MS from 1981 to 2007. The analysis considered household income and parental education levels when participants were 15 years old. While no significant correlation was found between childhood socioeconomic status and MS, a subtle trend indicated a reduced MS risk among children of better-educated parents, particularly mothers. Specifically, children whose mothers had completed secondary education experienced a 5% lower risk of developing MS, while those whose mothers achieved higher education had a 14% reduced risk (32). Furthermore, a systematic review encompassing 21 studies from 13 countries examined the association between MS and socioeconomic status. The findings from five studies conducted in countries with high inequality revealed an association between high socioeconomic status and MS. In contrast, 16 studies from more egalitarian countries found either no association or a link with low socioeconomic status. The link between high socioeconomic status and increased MS risk appears stronger in nations with greater inequality. However, the failure of many studies to control for other MS risk factors makes it challenging to draw definitive conclusions (33).

The percentage of MS-related deaths that were attributable to smoking rose with age, peaking in the 55–59-year-old age group. Cigarette smoking is known to initiate a pro-inflammatory reaction in the lungs, potentially increasing the risk of MS by fostering a possible cross-reactivity between lung and myelin antigens. Moreover, some compounds found in cigarette smoke might directly damage neurons. Among MS patients who smoke, there is evidence of more aggressive disease activity, accelerated brain degeneration, and increased disability (34). In-depth studies of the mechanisms connecting smoking and the deterioration of MS are urgently needed. However, it is clear that reducing smoking rates among MS patients is crucial (34). Additionally, another study highlighted that smokers demonstrated an elevated risk of MS progression (35). In a case–control study involving 9,419 MS patients, it was revealed that 13.1% of MS cases could be attributed to smoking. The attributable risk was higher in men (19.1%) than women (10.6%), while ex-smokers exhibited a minimal risk (0.6%). The study also found that avoiding tobacco smoking could potentially prevent at least 13% of MS cases (36). Another meta-analysis suggested that smoking has a causal role in both the onset and progression of MS. In light of this, anti-smoking campaigns should highlight MS as a significant health concern to underscore the importance of reducing smoking rates (37). Therefore, preventive programs targeting smoking could potentially reduce the incidence and burden of MS in the coming years. Furthermore, there are additional risk factors for MS, such as vitamin D deficiency (38), excess body weight (39), and insufficient physical activity (40, 41) that could be considered in the development of preventive programs for MS. Additionally, nutrition and diet may influence the development and progression of MS by impacting gut bacteria, enzyme functions, and factors related to vascular issues in MS patients. Studies suggest that a balanced diet, coupled with a healthy lifestyle, can improve various clinical indicators and elevate quality of life for individuals with MS (42). The risk of developing MS is influenced by genetics, and the primary genes associated with this risk are located within the major histocompatibility complex (43). It is important to acknowledge that MS is a multifactorial disease that is influenced by a combination of environmental and genetic factors. Unlike genetic determinants, environmental elements and lifestyle choices are modifiable risk factors. Therefore, making lifestyle modifications, maintaining a healthy weight, adding vitamin D supplements and increasing physical activity can help to prevent the onset of MS, as well as its progression (44).

Standard treatments for MS have traditionally aimed to manage symptoms and slow disease progression. Among these treatments, disease-modifying therapies (DMTs) like interferon beta and glatiramer acetate are commonly prescribed to reduce the frequency of relapses. Corticosteroids, like methylprednisolone, are employed during acute MS flare-ups to reduce inflammation. Additionally, physical therapy and treatments addressing fatigue and spasticity play crucial roles in comprehensive MS management (45, 46). Over the years, novel MS therapies have dramatically transformed the disease burden, particularly with the approval of B cell-targeted treatments, such as anti-CD20 therapies in high-income regions. Without considering the impact of these disease-modifying therapies, it is difficult to fully assess the overall burden of the disease. The reduction in MS burden is largely due to advancements in its management, especially the recognition of B cells’ key role in the pathogenesis of the disorder. B cells contribute to MS by activating pro-inflammatory T cells, secreting cytokines, and producing myelin-targeting autoantibodies. Therapies targeting B cell depletion, notably anti-CD20 monoclonal antibodies, have emerged as effective treatments. Ocrelizumab, an anti-CD20 therapy, has demonstrated significant success in reducing relapse rates and radiological markers in relapsing–remitting MS (RRMS), as well as lowering disability progression in primary progressive MS (PPMS). In 2017, Ocrelizumab became the first FDA-approved treatment for PPMS, marking a significant milestone in MS treatment (5, 47, 48).

This study has several limitations that should be considered. Firstly, the reliance on data sourced from the Global Burden of Disease study may be problematic, as it may not fully capture the true global and regional variation in multiple sclerosis (MS) rates. Inaccuracies in data collection from underdeveloped regions with limited healthcare infrastructure, along with the lack of comprehensive national studies in wealthier countries, can skew the reported prevalence and burden of MS. The use of GBD prediction models, which may lack critical variables necessary for a comprehensive risk assessment, could lead to incomplete or biased conclusions about the distribution and risk factors of MS. Another limitation arises from the absence of reliable predictive factors for MS, hindering thorough population-based risk evaluations. This issue is partly due to the scarcity of extensive longitudinal data on neurological disability that accurately represents the diverse demographics of multiple sclerosis. Thirdly, the GBD study focused solely on smoking as an attributable risk factor for MS, neglecting other potential risk factors such as obesity and nutritional deficiencies. This narrow focus may overlook the complex interplay of factors like obesity, nutrition, and physical activity, potentially introducing bias in assessing the full spectrum of modifiable risks associated with MS.

## Conclusion

Despite a decline in the age-standardized rate of MS over the period 1990–2019, the disease continues to impose a substantial burden, with a significant number of DALYs and prevalent cases. Moreover, about 14% of the MS burden is due to smoking and this figure is even higher among men. Therefore, healthcare planning and resource distribution should take into account MS and its associated risk factors, particularly in areas with high levels of socioeconomic development.

## Author note

This study is based on publicly available data and solely reflects the opinion of its authors and not that of the Institute for Health Metrics and Evaluation.

## Data Availability

The datasets presented in this study can be found in online repositories. The names of the repository/repositories and accession number(s) can be found at: https://vizhub.healthdata.org/gbd-results/.

## Supplementary material

SUPPLEMENTARY FIGURE S1 The age-standardized point prevalence of multiple sclerosis per 100 000 population in 2019 for the 21 Global Burden of Disease regions, by sex. (Generated using data available from: https://ghdx.healthdata.org/gbd-results-tool).

SUPPLEMENTARY FIGURE S2 Number of prevalent cases of multiple sclerosis from 1990 to 2019 for the 21 Global Burden of Disease regions. (Generated using data available from: https://ghdx.healthdata.org/gbd-results-tool).

SUPPLEMENTARY FIGURE S3 The age-standardized death rate of multiple sclerosis per 100 000 population in 2019 for the 21 Global Burden of Disease regions, by sex. (Generated using data available from: https://ghdx.healthdata.org/gbd-results-tool).

SUPPLEMENTARY FIGURE S4 Number of deaths of multiple sclerosis from 1990 to 2019 for the 21 Global Burden of Disease regions. (Generated using data available from: https://ghdx.healthdata.org/gbd-results-tool).

SUPPLEMENTARY FIGURE S5 The age-standardized disability-adjusted life years (DALYs) rate of multiple sclerosis per 100 000 population in 2019 for the 21 Global Burden of Disease regions, by sex. (Generated using data available from: https://ghdx.healthdata.org/gbd-results-tool).

SUPPLEMENTARY FIGURE S6 Number of disability-adjusted life years (DALYs) of multiple sclerosis from 1990 to 2019 for the 21 Global Burden of Disease regions. (Generated using data available from: https://ghdx.healthdata.org/gbd-results-tool).

SUPPLEMENTARY FIGURE S7 The percentage change in age-standardized point prevalence of multiple sclerosis from 1990 to 2019 for the 21 Global Burden of Disease regions, by sex. (Generated using data available from: https://ghdx.healthdata.org/gbd-results-tool).

SUPPLEMENTARY FIGURE S8 The percentage change in age-standardized death rate of multiple sclerosis from 1990 to 2019 for the 21 Global Burden of Disease regions, by sex. (Generated using data available from: https://ghdx.healthdata.org/gbd-results-tool).

SUPPLEMENTARY FIGURE S9 The percentage change in age-standardized disability-adjusted life years (DALYs) rate of multiple sclerosis from 1990 to 2019 for the 21 Global Burden of Disease regions, by sex. (Generated using data available from: https://ghdx.healthdata.org/gbd-results-tool).

SUPPLEMENTARY FIGURE S10 Global number of death cases and age-standardized death rate of multiple sclerosis per 100 000 population by age and sex, 2019; dotted and dashed lines indicate 95% upper and lower UIs, respectively. (Generated using data available from: https://ghdx.healthdata.org/gbd-results-tool).

SUPPLEMENTARY FIGURE S11 Global number of disability-adjusted life years (DALYs) and age-standardized disability-adjusted life years (DALYs) rate of multiple sclerosis per 100 000 population by age and sex, 2019; dotted and dashed lines indicate 95% upper and lower UIs, respectively. (Generated using data available from: https://ghdx.healthdata.org/gbd-results-tool).

SUPPLEMENTARY FIGURE S12 age-standardized DALY rates of multiple sclerosis per 100 000 population for the 204 countries and territories and sociodemographic Index, 2019; expected values are shown as the black line. DALY, disability-adjusted life year. (Generated using data available from: https://ghdx.healthdata.org/gbd-results-tool).

SUPPLEMENTARY FIGURE S13 Percentage of multiple sclerosis-related age-standardized DALYs that were due to smoking for the 21 Global Burden of Disease regions, by sex and age, 2019. DALY, disability-adjusted life year. (Generated using data available from: https://ghdx.healthdata.org/gbd-results-tool).

SUPPLEMENTARY FIGURE S14 Percentage of multiple sclerosis-related age-standardized deaths that were attributable to smoking for the 21 Global Burden of Disease regions, by sex and age, 2019. DALY, disability-adjusted life year. (Generated using data available from: https://ghdx.healthdata.org/gbd-results-tool).

SUPPLEMENTARY FIGURE S15 Percentage of multiple sclerosis-related age-standardized DALYs that were due to smoking for the 21 Global Burden of Disease regions, by sex and region, 2019. DALY, disability-adjusted life year. (Generated using data available from: https://ghdx.healthdata.org/gbd-results-tool).

SUPPLEMENTARY FIGURE S16 Percentage of multiple sclerosis-related age-standardized deaths that were due to smoking for the 21 Global Burden of Disease regions, by sex and region, 2019. DALY, disability-adjusted life year. (Generated using data available from: https://ghdx.healthdata.org/gbd-results-tool).
