# Supplementary figures and images for: Global burden of multiple sclerosis and its attributable risk factors, 1990–2019
Source: Front Neurol. 2024 Oct 25;15:1448377. doi: 10.3389/fneur.2024.1448377 (PMC11545682; doi:10.3389/fneur.2024.1448377)

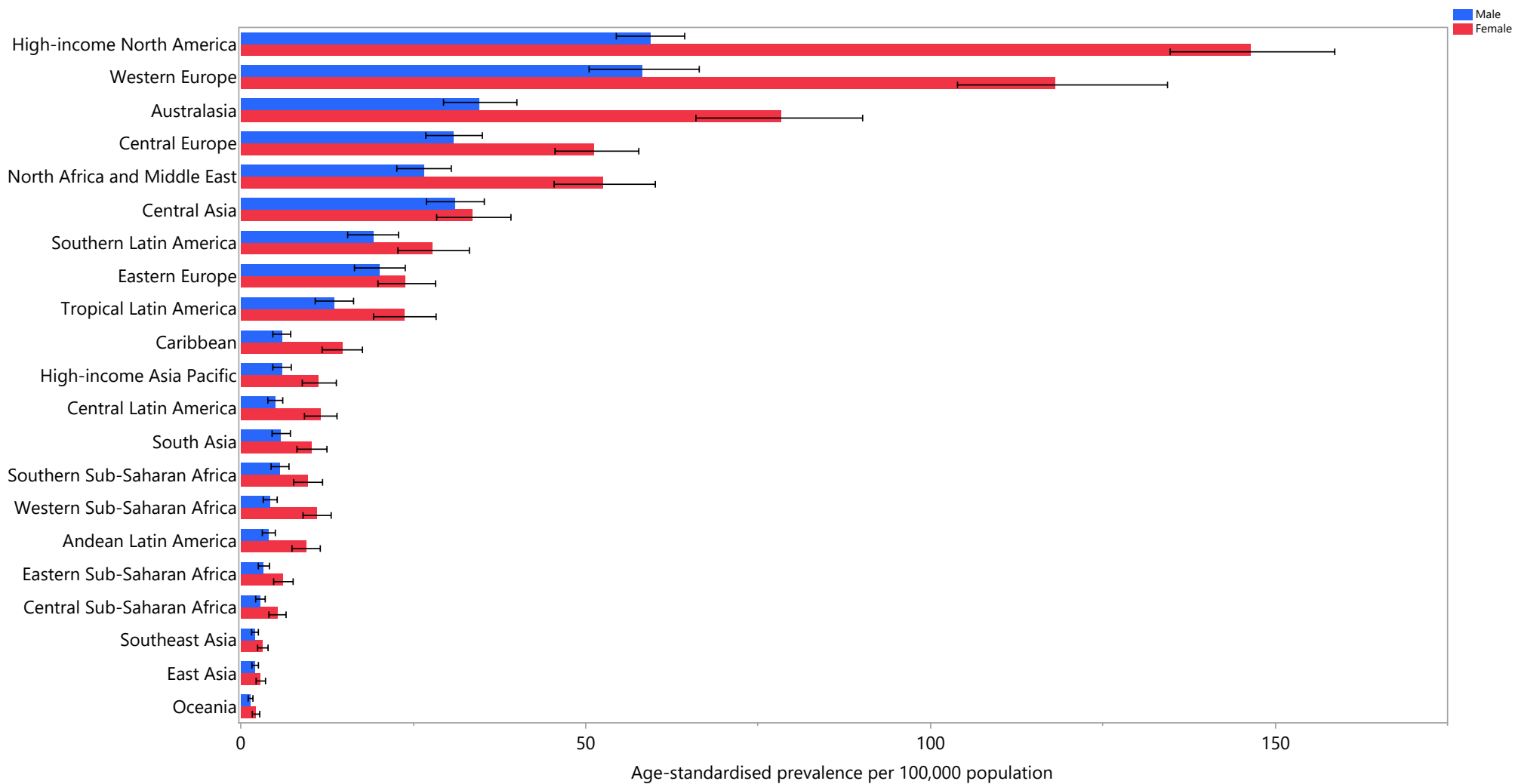

Supplement: SUPPLEMENTARY FIGURE S1 — The age-standardized point prevalence of multiple sclerosis per 100 000 population in 2019 for the 21 Global Burden of Disease regions, by sex. (Generated using data available from: https://ghdx.healthdata.org/gbd-results-tool). [file Data_Sheet_1.PDF]

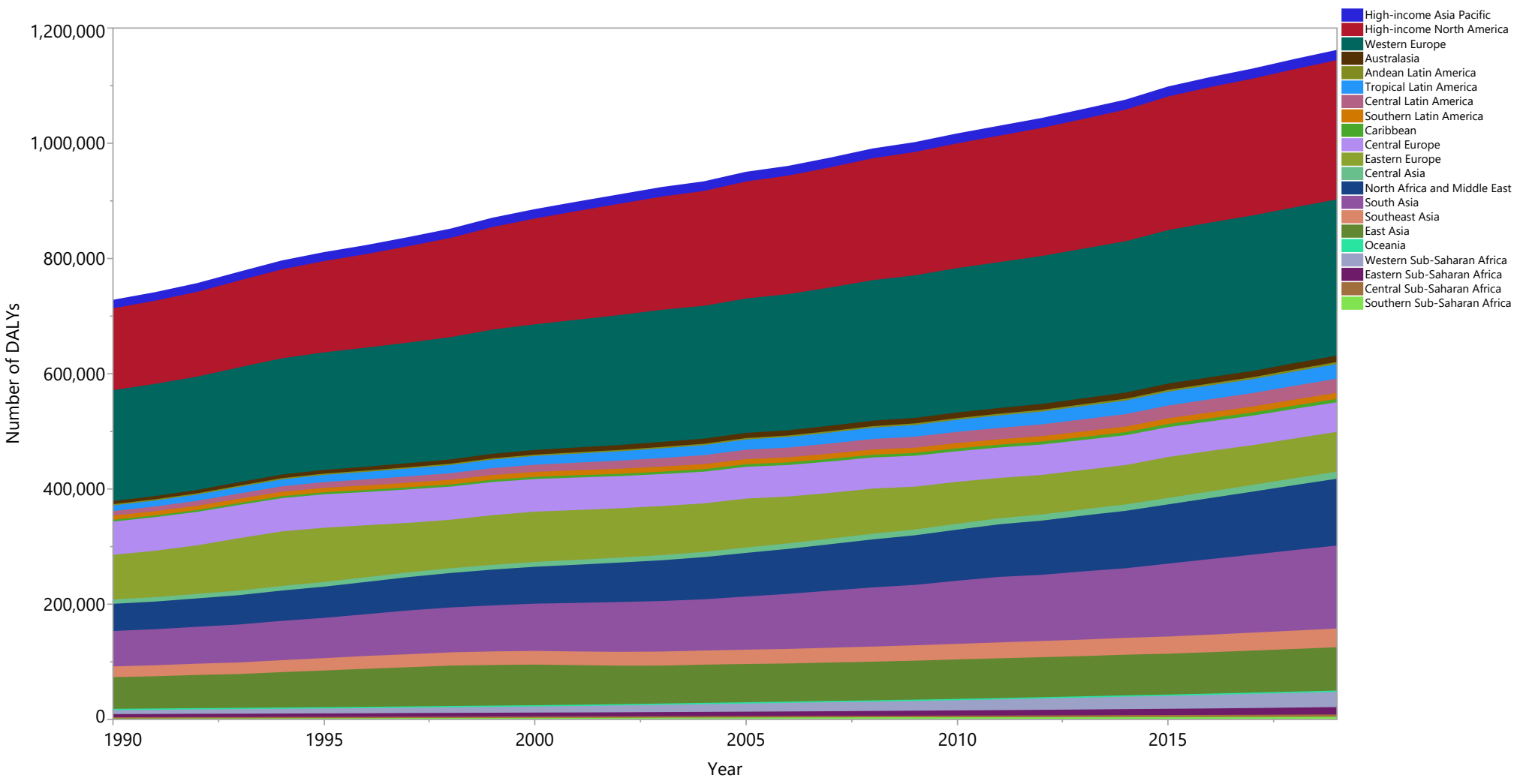

Supplement: SUPPLEMENTARY FIGURE S2 — Number of prevalent cases of multiple sclerosis from 1990 to 2019 for the 21 Global Burden of Disease regions. (Generated using data available from: https://ghdx.healthdata.org/gbd-results-tool). [file Data_Sheet_2.PDF]

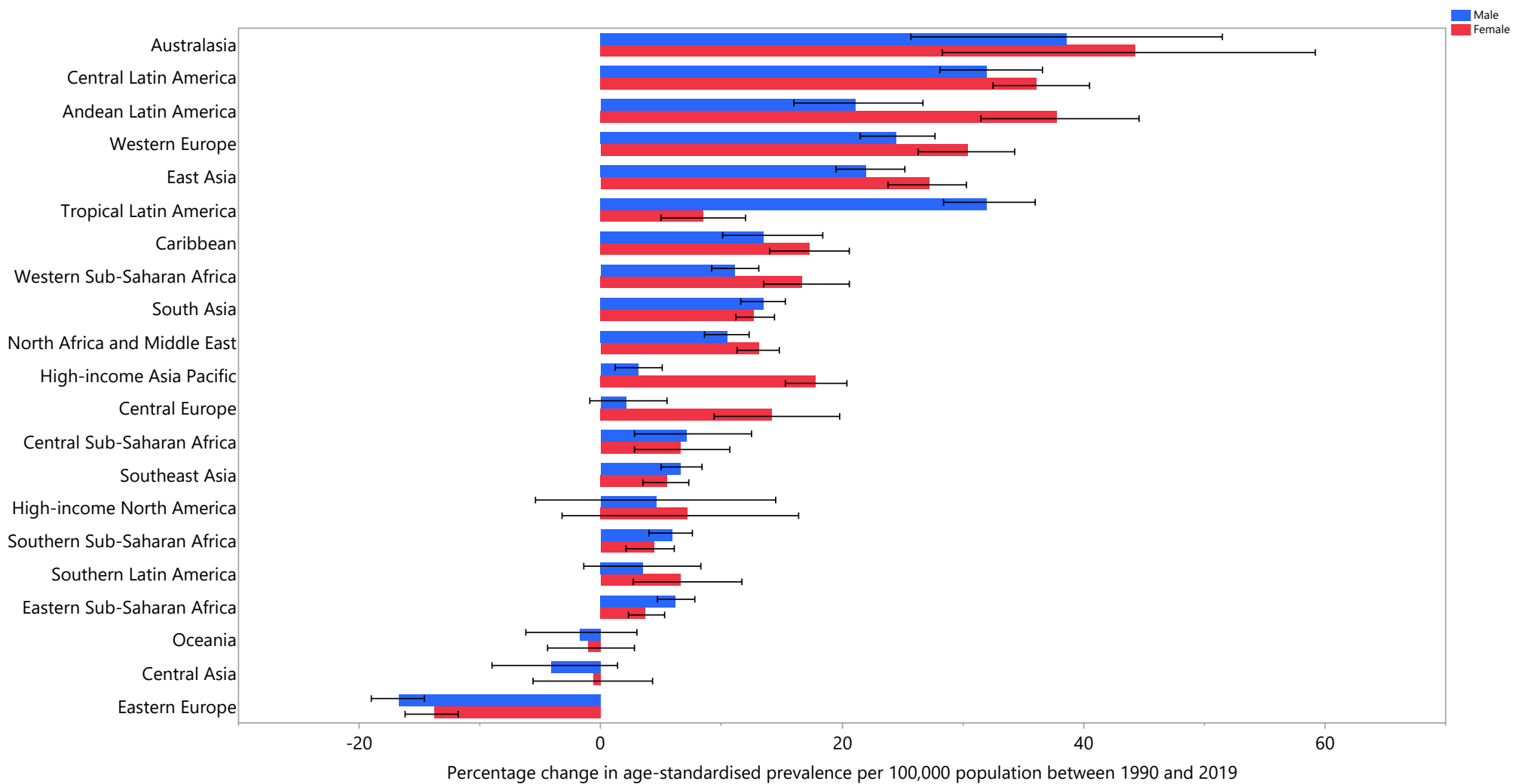

Supplement: SUPPLEMENTARY FIGURE S3 — The age-standardized death rate of multiple sclerosis per 100 000 population in 2019 for the 21 Global Burden of Disease regions, by sex. (Generated using data available from: https://ghdx.healthdata.org/gbd-results-tool). [file Data_Sheet_3.PDF]

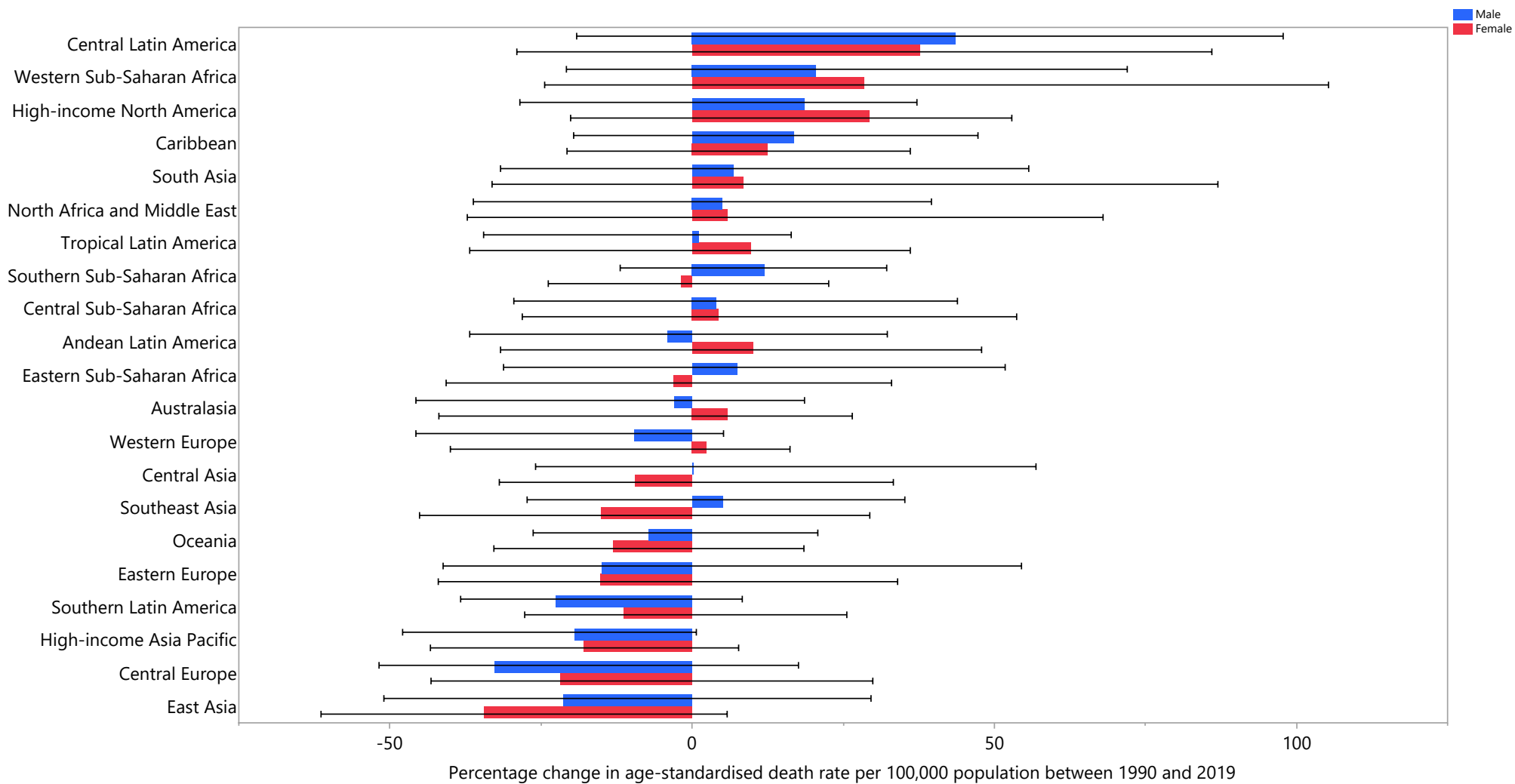

Supplement: SUPPLEMENTARY FIGURE S4 — Number of deaths of multiple sclerosis from 1990 to 2019 for the 21 Global Burden of Disease regions. (Generated using data available from: https://ghdx.healthdata.org/gbd-results-tool). [file Data_Sheet_4.PDF]

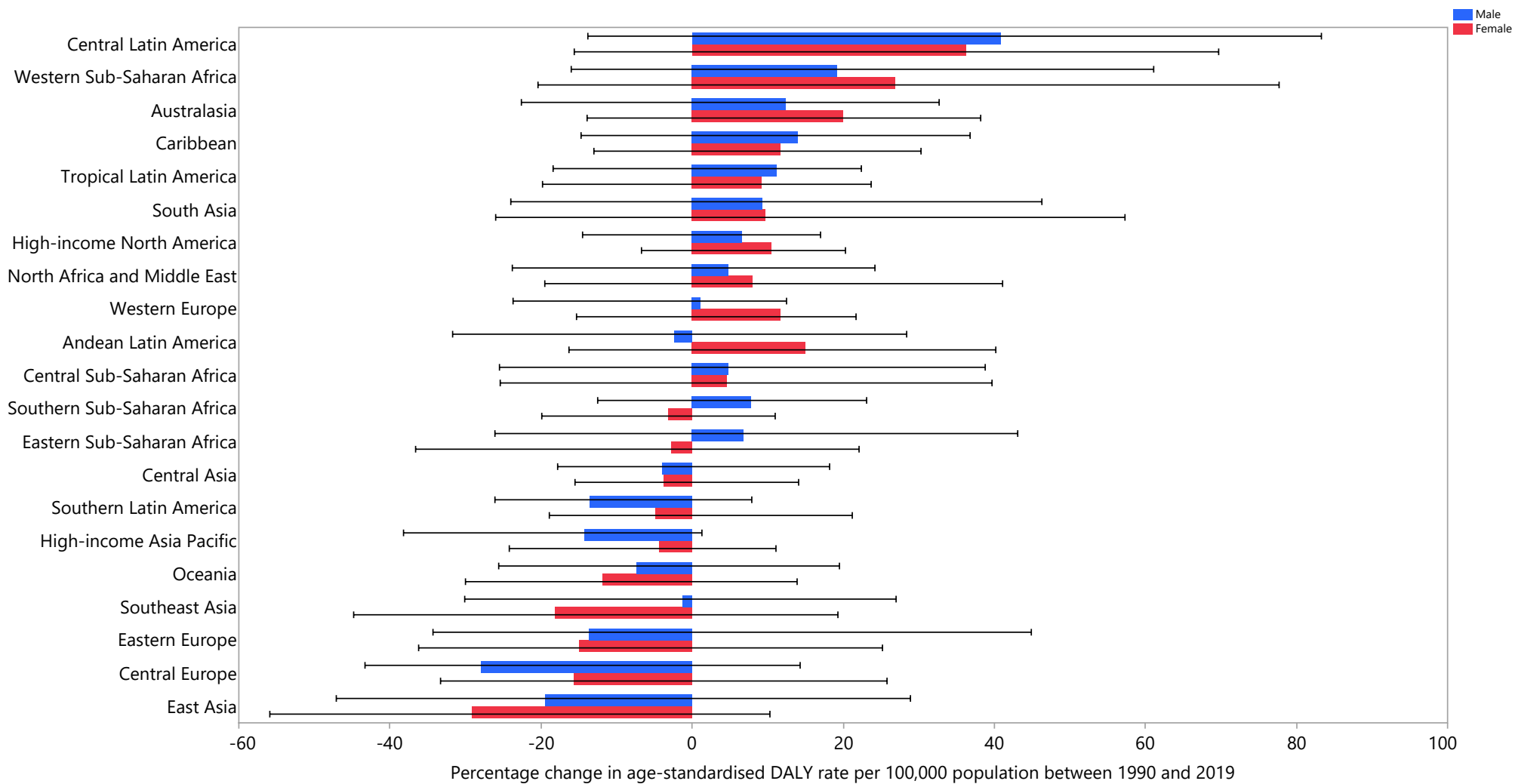

Supplement: SUPPLEMENTARY FIGURE S5 — The age-standardized disability-adjusted life years (DALYs) rate of multiple sclerosis per 100 000 population in 2019 for the 21 Global Burden of Disease regions, by sex. (Generated using data available from: https://ghdx.healthdata.org/gbd-results-tool). [file Data_Sheet_5.PDF]

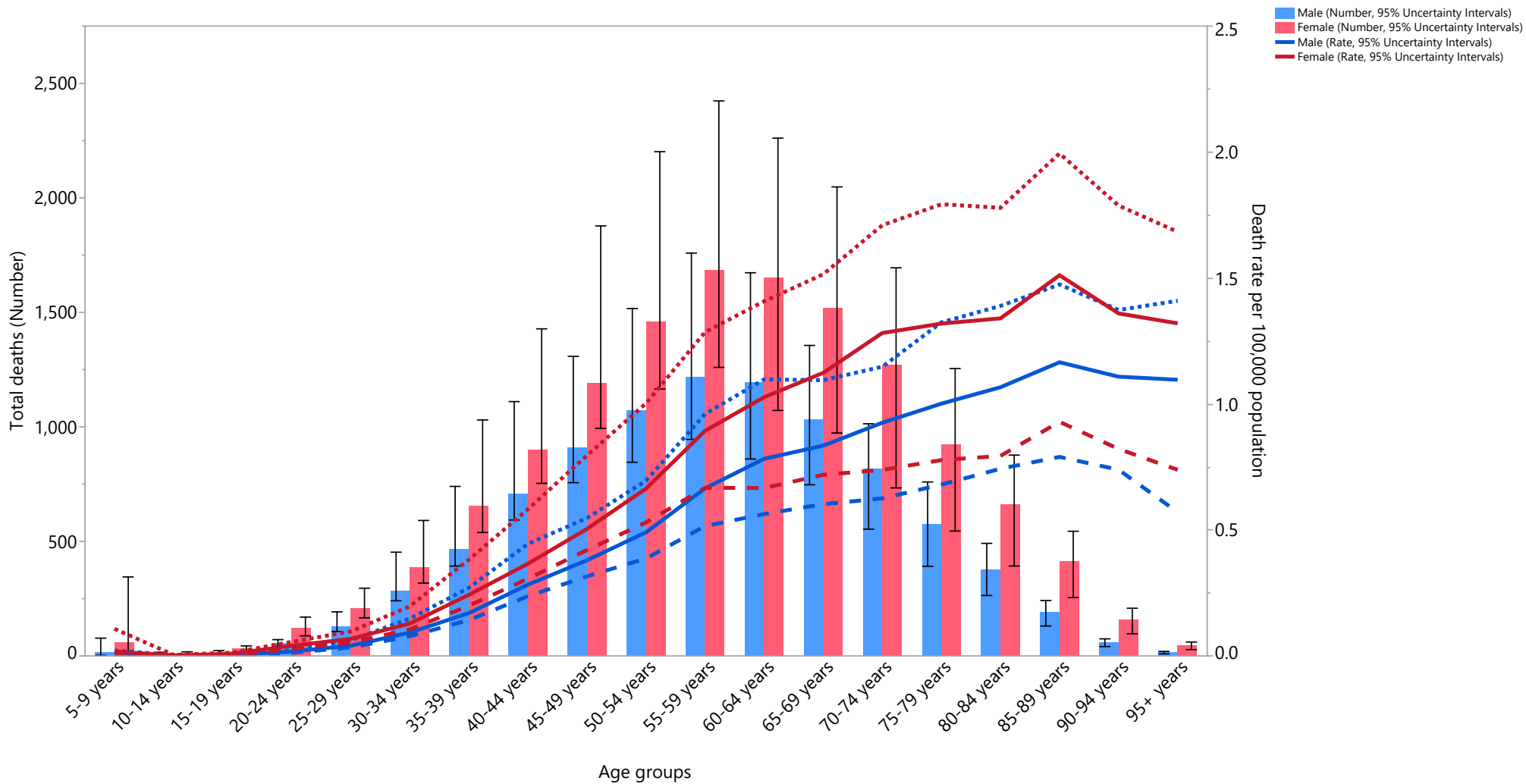

Supplement: SUPPLEMENTARY FIGURE S6 — Number of disability-adjusted life years (DALYs) of multiple sclerosis from 1990 to 2019 for the 21 Global Burden of Disease regions. (Generated using data available from: https://ghdx.healthdata.org/gbd-results-tool). [file Data_Sheet_6.PDF]

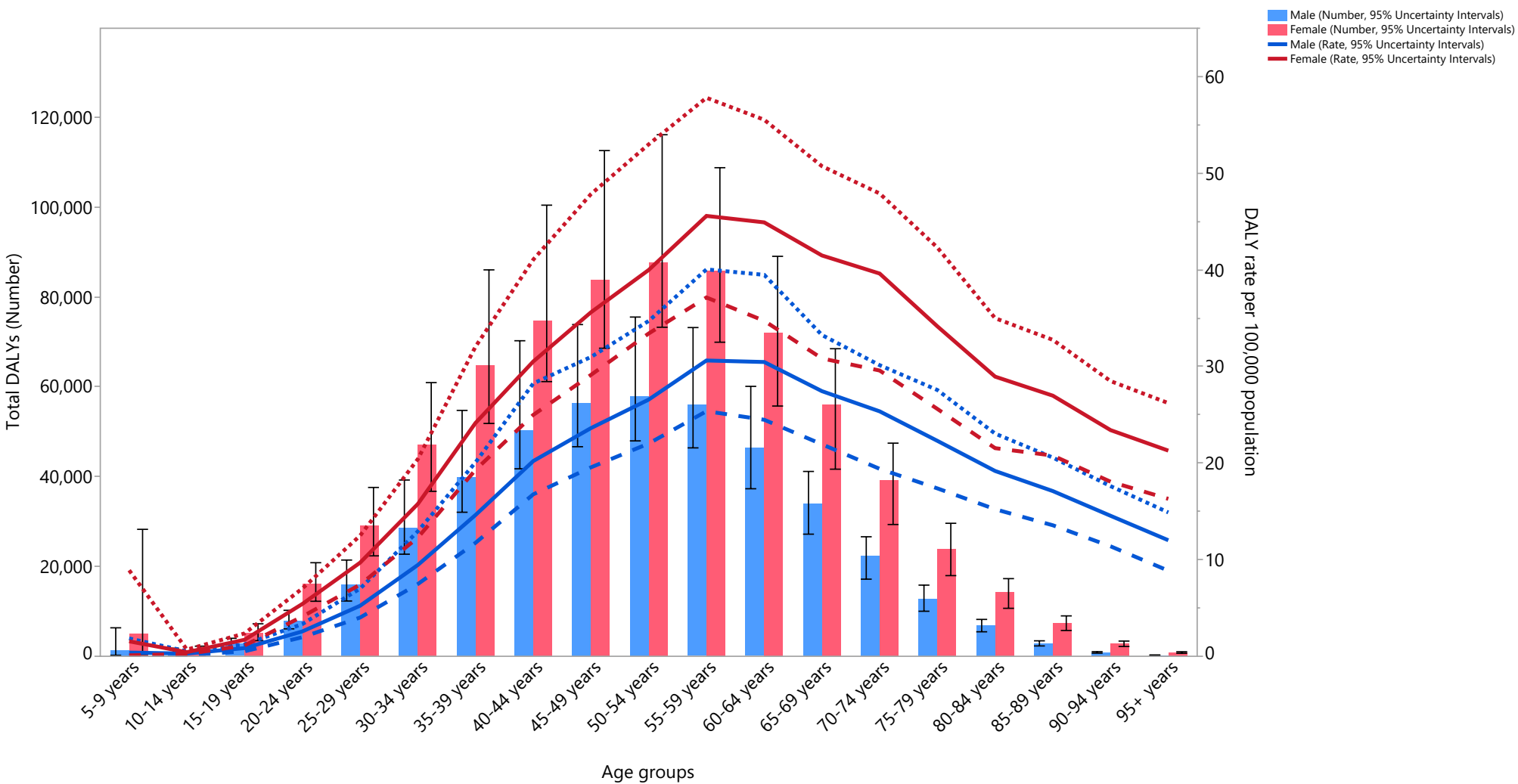

Supplement: SUPPLEMENTARY FIGURE S7 — The percentage change in age-standardized point prevalence of multiple sclerosis from 1990 to 2019 for the 21 Global Burden of Disease regions, by sex. (Generated using data available from: https://ghdx.healthdata.org/gbd-results-tool). [file Data_Sheet_7.PDF]

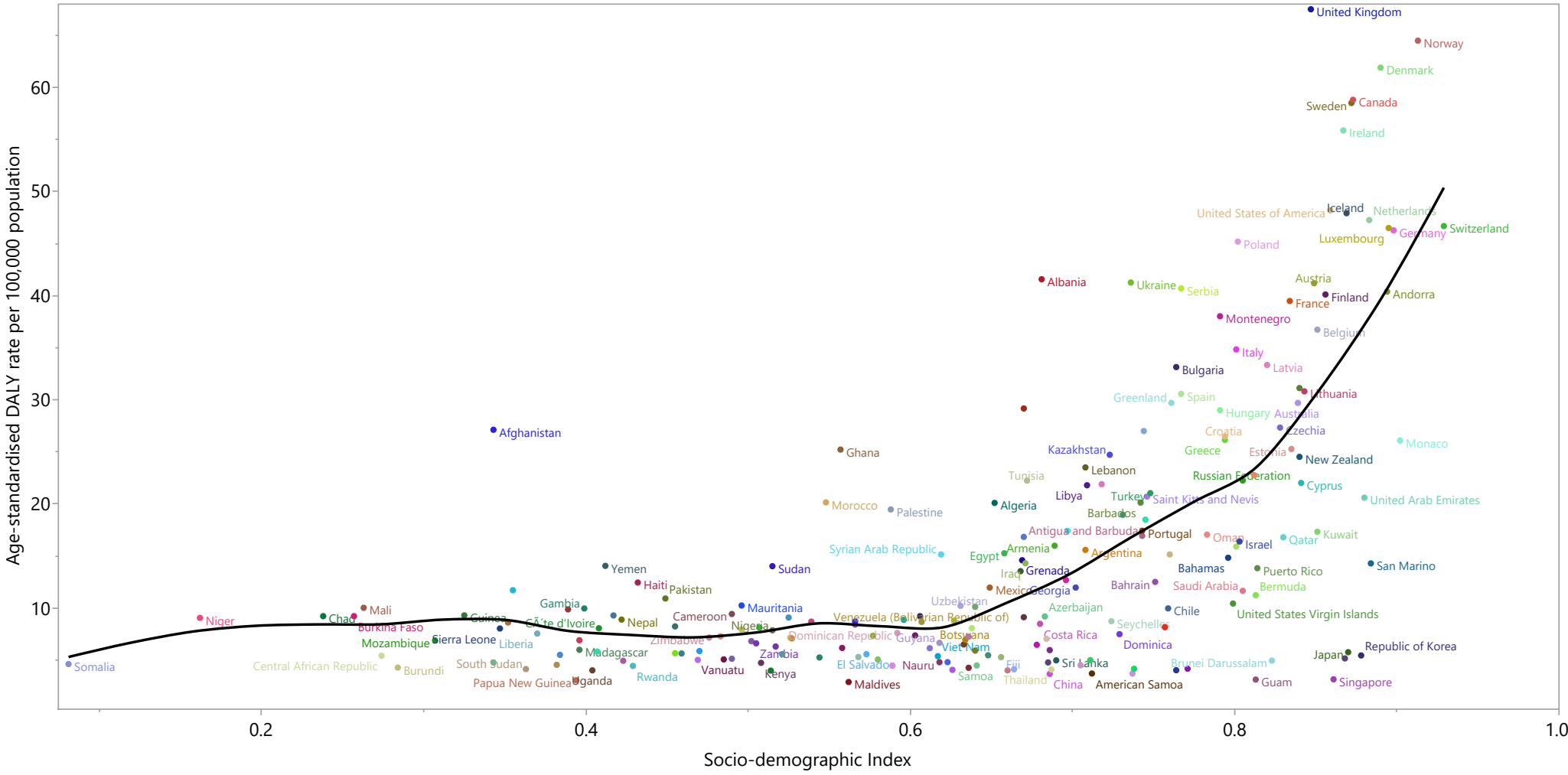

Supplement: SUPPLEMENTARY FIGURE S8 — The percentage change in age-standardized death rate of multiple sclerosis from 1990 to 2019 for the 21 Global Burden of Disease regions, by sex. (Generated using data available from: https://ghdx.healthdata.org/gbd-results-tool). [file Data_Sheet_8.PDF]

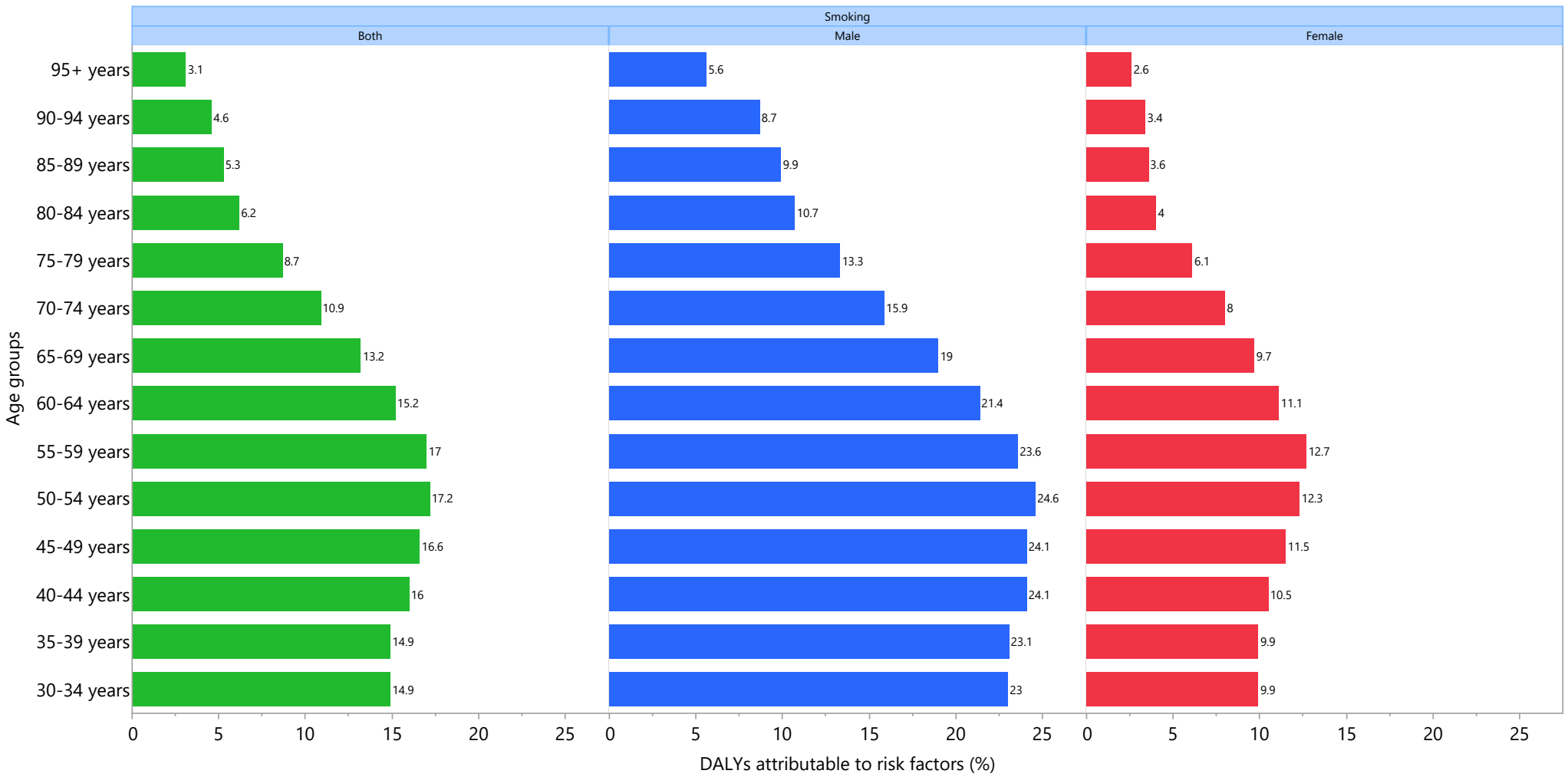

Supplement: SUPPLEMENTARY FIGURE S9 — The percentage change in age-standardized disability-adjusted life years (DALYs) rate of multiple sclerosis from 1990 to 2019 for the 21 Global Burden of Disease regions, by sex. (Generated using data available from: https://ghdx.healthdata.org/gbd-results-tool). [file Data_Sheet_9.PDF]

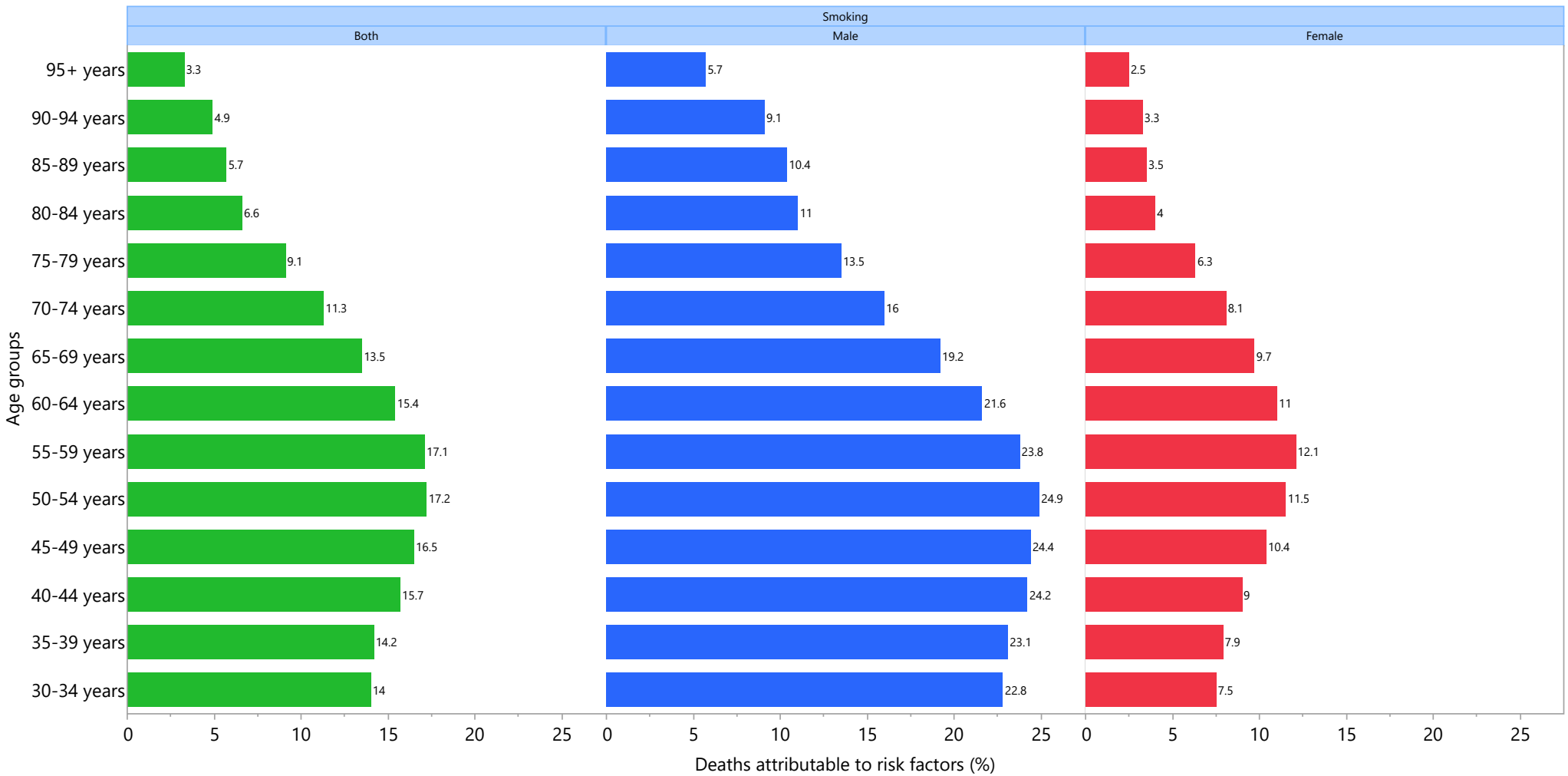

Supplement: SUPPLEMENTARY FIGURE S10 — Global number of death cases and age-standardized death rate of multiple sclerosis per 100 000 population by age and sex, 2019; dotted and dashed lines indicate 95% upper and lower UIs, respectively. (Generated using data available from: https://ghdx.healthdata.org/gbd-results-tool). [file Data_Sheet_10.PDF]

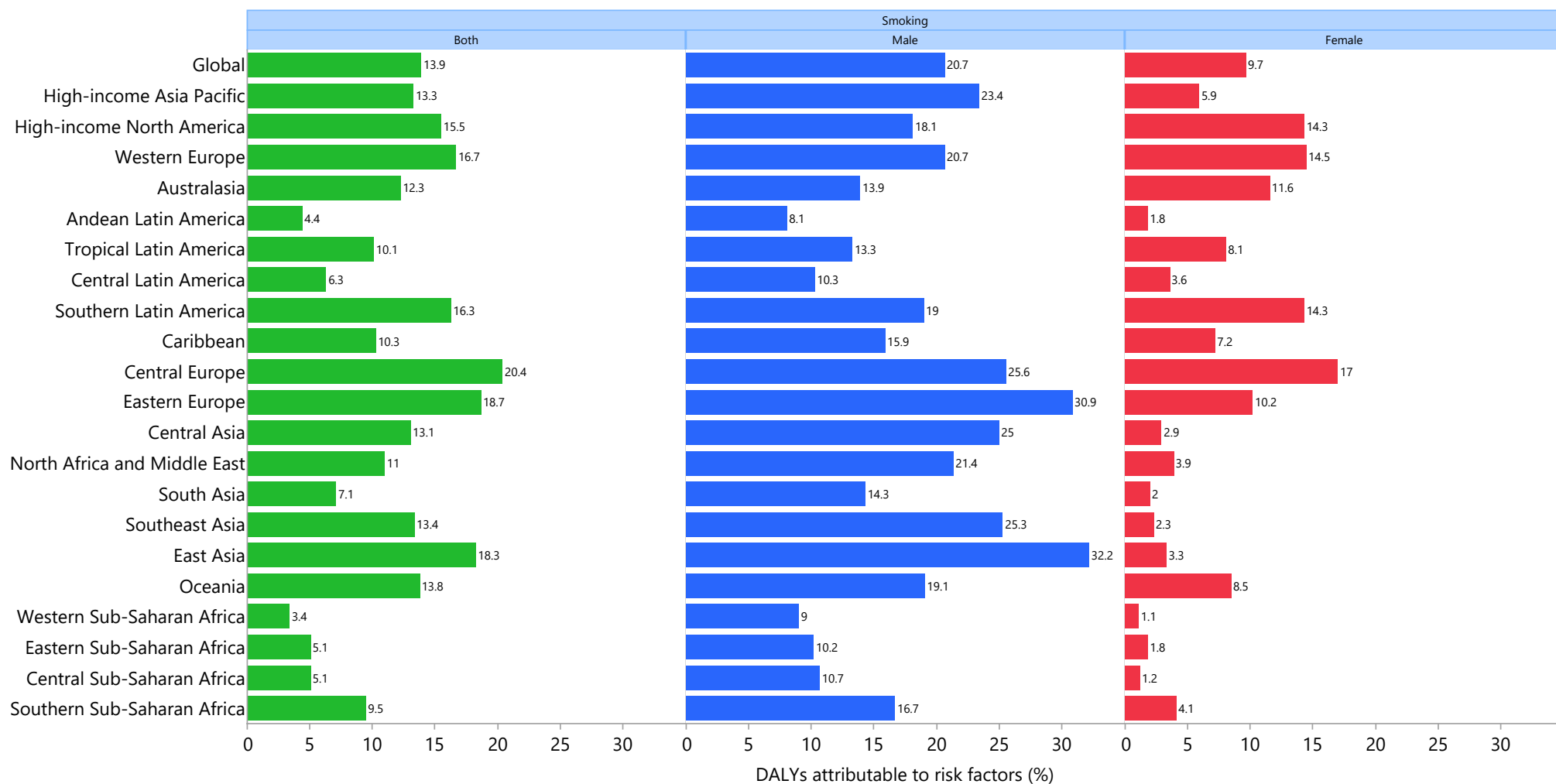

Supplement: SUPPLEMENTARY FIGURE S11 — Global number of disability-adjusted life years (DALYs) and age-standardized disability-adjusted life years (DALYs) rate of multiple sclerosis per 100 000 population by age and sex, 2019; dotted and dashed lines indicate 95% upper and lower UIs, respectively. (Generated using data available from: https://ghdx.healthdata.org/gbd-results-tool). [file Data_Sheet_11.PDF]

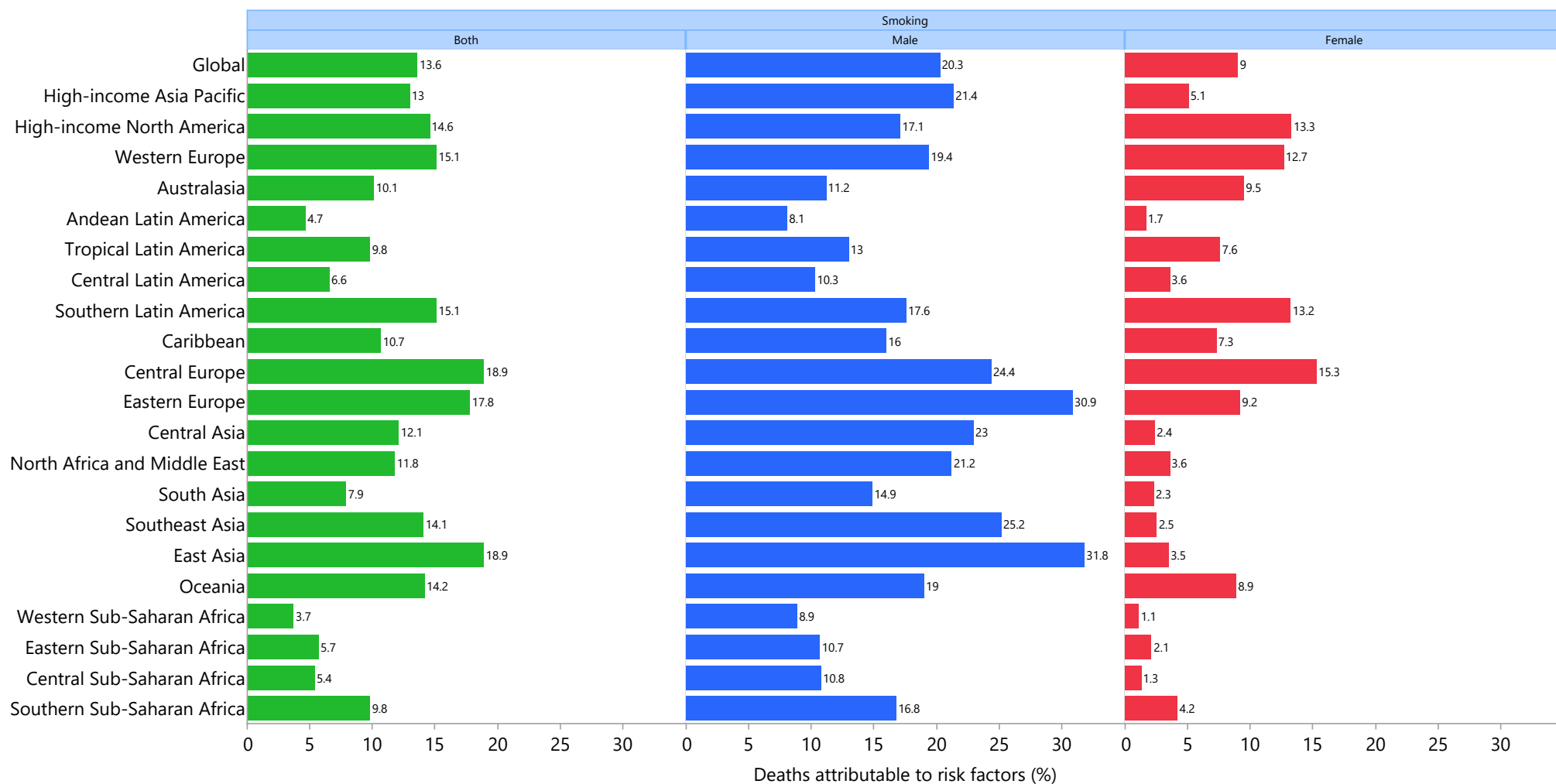

Supplement: SUPPLEMENTARY FIGURE S12 — age-standardized DALY rates of multiple sclerosis per 100 000 population for the 204 countries and territories and sociodemographic Index, 2019; expected values are shown as the black line. DALY, disability-adjusted life year. (Generated using data available from: https://ghdx.healthdata.org/gbd-results-tool). [file Data_Sheet_12.PDF]

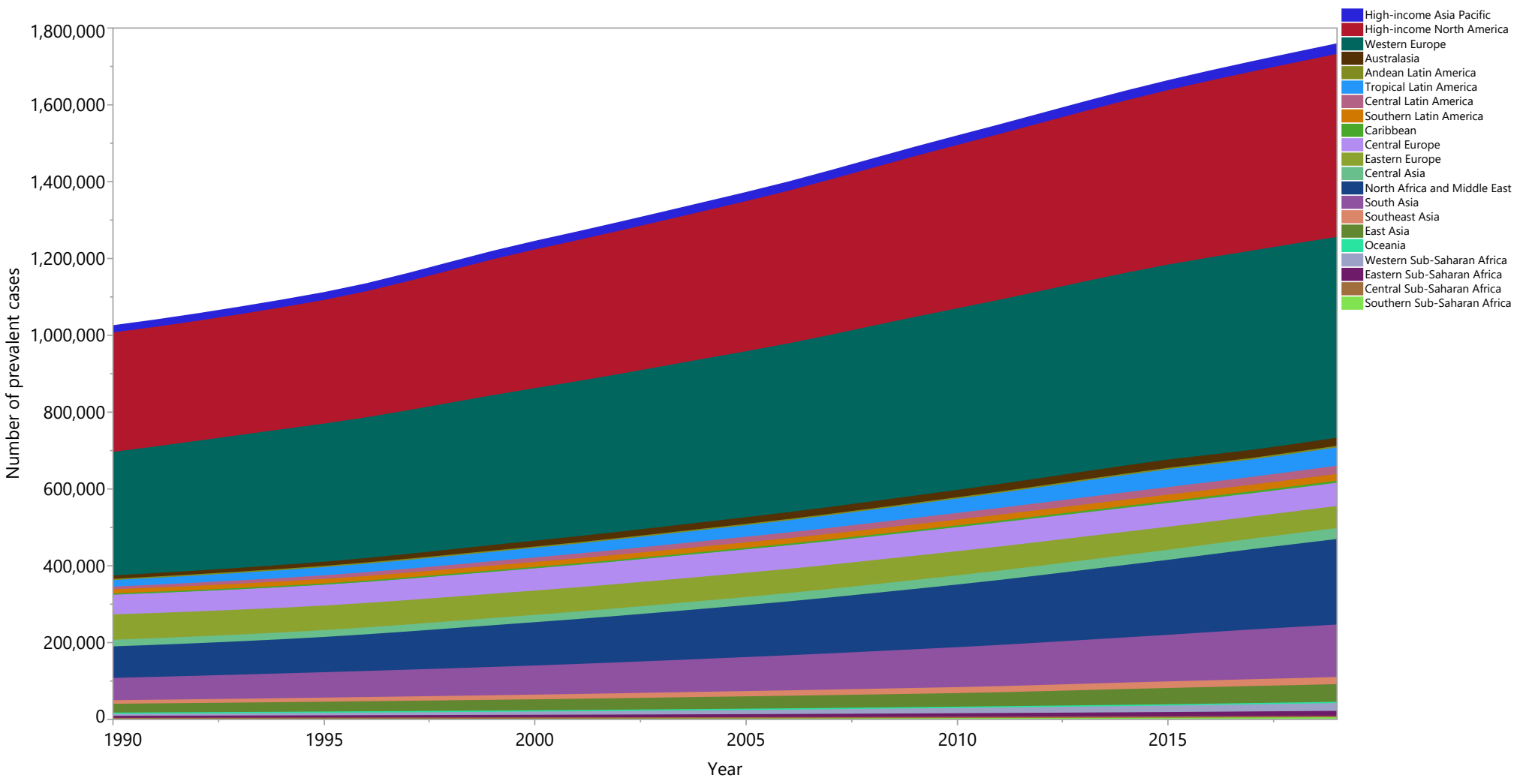

Supplement: SUPPLEMENTARY FIGURE S13 — Percentage of multiple sclerosis-related age-standardized DALYs that were due to smoking for the 21 Global Burden of Disease regions, by sex and age, 2019. DALY, disability-adjusted life year. (Generated using data available from: https://ghdx.healthdata.org/gbd-results-tool). [file Data_Sheet_13.PDF]

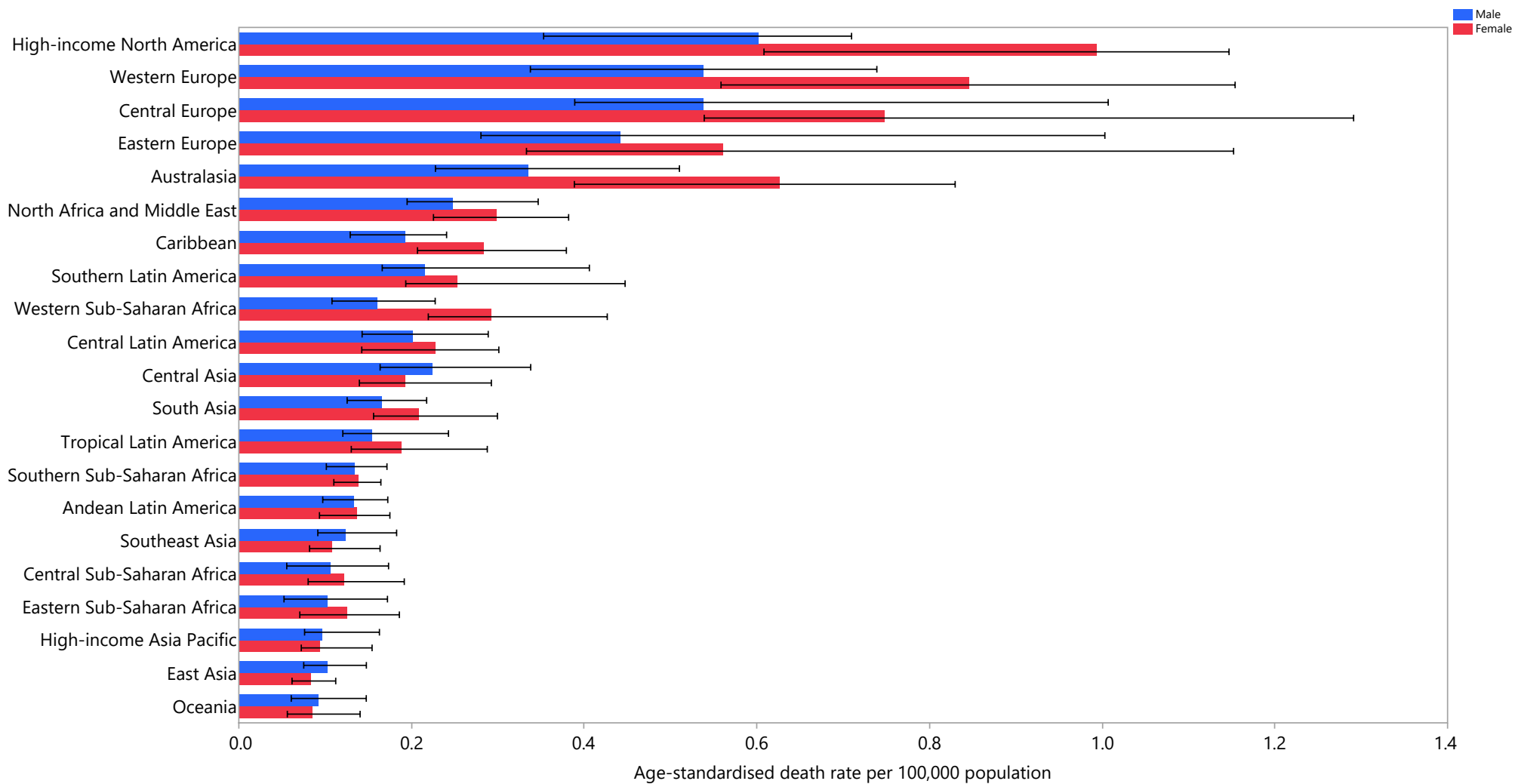

Supplement: SUPPLEMENTARY FIGURE S14 — Percentage of multiple sclerosis-related age-standardized deaths that were attributable to smoking for the 21 Global Burden of Disease regions, by sex and age, 2019. DALY, disability-adjusted life year. (Generated using data available from: https://ghdx.healthdata.org/gbd-results-tool). [file Data_Sheet_14.PDF]

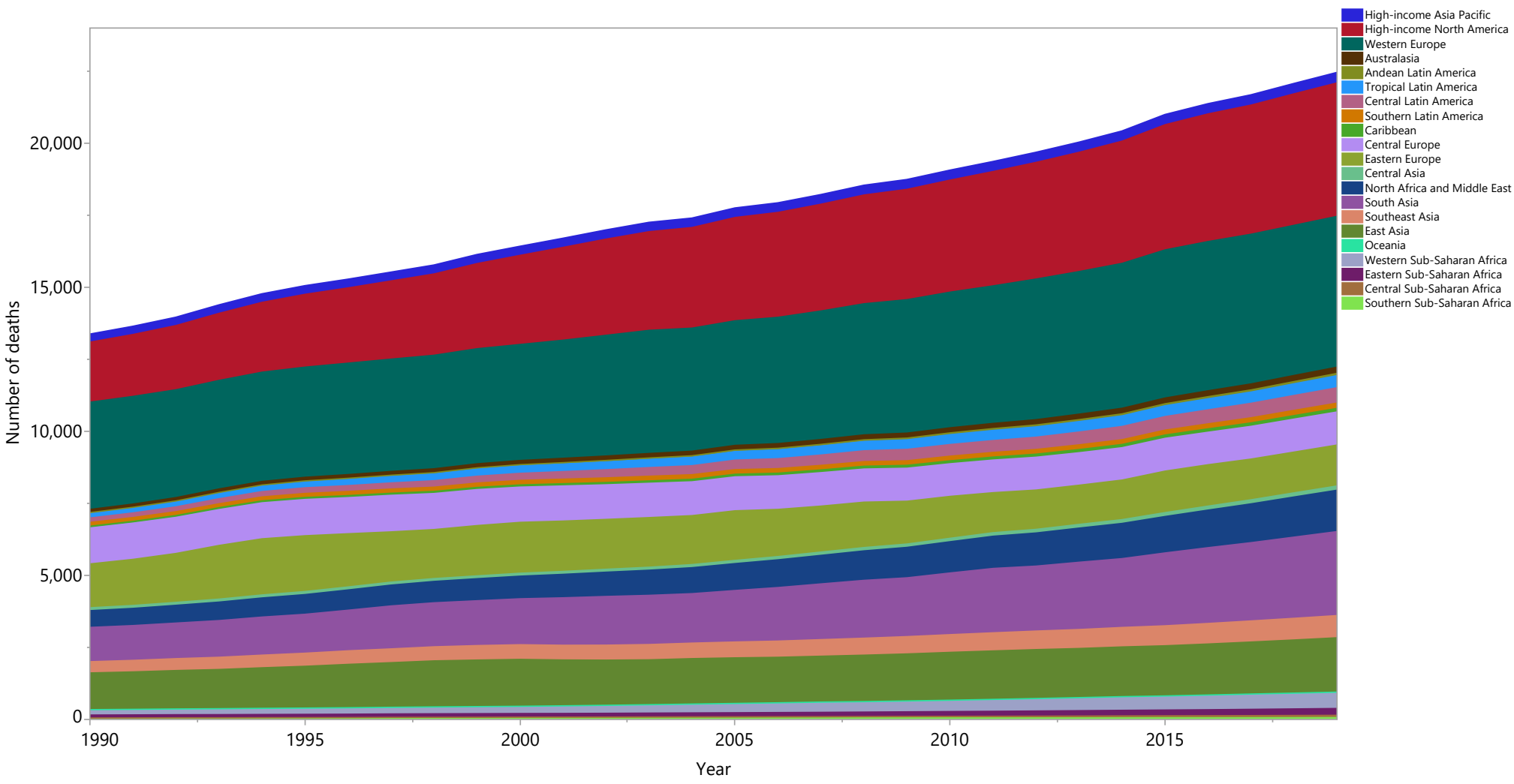

Supplement: SUPPLEMENTARY FIGURE S15 — Percentage of multiple sclerosis-related age-standardized DALYs that were due to smoking for the 21 Global Burden of Disease regions, by sex and region, 2019. DALY, disability-adjusted life year. (Generated using data available from: https://ghdx.healthdata.org/gbd-results-tool). [file Data_Sheet_15.PDF]

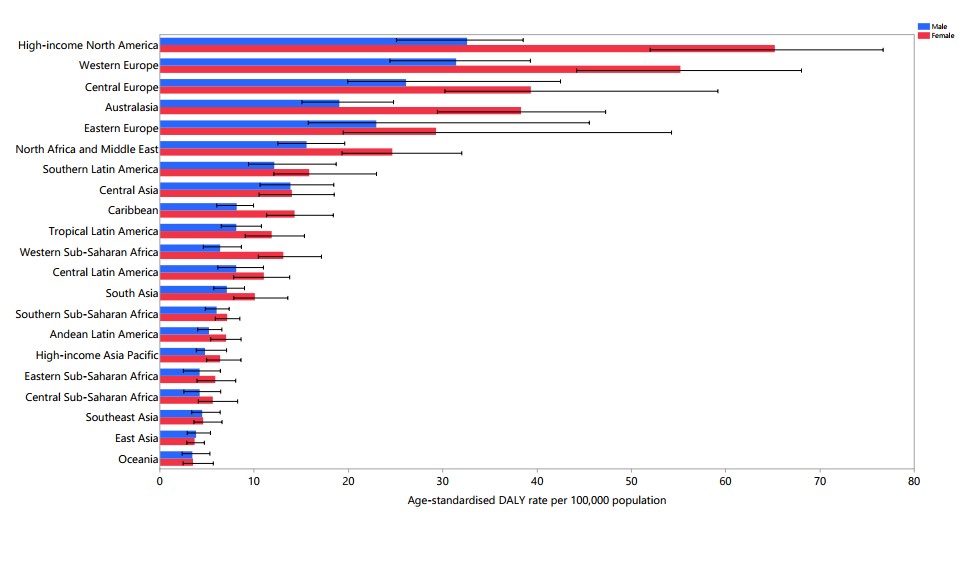

Supplement: SUPPLEMENTARY FIGURE S16 — Percentage of multiple sclerosis-related age-standardized deaths that were due to smoking for the 21 Global Burden of Disease regions, by sex and region, 2019. DALY, disability-adjusted life year. (Generated using data available from: https://ghdx.healthdata.org/gbd-results-tool). [file Image_1.JPEG]
