# Supplementary material for: Global burden of multiple sclerosis and its attributable risk factors, 1990–2019
Source: Front Neurol. 2024 Oct 25;15:1448377. doi: 10.3389/fneur.2024.1448377 (PMC11545682; doi:10.3389/fneur.2024.1448377)
Supplement: Supplementary file 17 [file Table_1.DOC]

| **Table S1: Covariates used in the CODEm models** | | | | |
| --- | --- | --- | --- | --- |
| Cause | Covariate Name | Transformation | Level | Direction |
| Multiple sclerosis | Absolute value of average latitude | None | 1 | 1 |
| Multiple sclerosis | Mean serum total cholesterol (mmol/L) | None | 2 | 1 |
| Multiple sclerosis | Health care access and quality index | None | 2 | -1 |
| Multiple sclerosis | Cumulative cigarettes (10 years) | None | 3 | 1 |
| Multiple sclerosis | Cumulative cigarettes (5 years) | None | 3 | 1 |
| Multiple sclerosis | Education (years per capita) | None | 3 | -1 |
| Multiple sclerosis | Log-transformed LDI (per capita) | None | 3 | -1 |
| Multiple sclerosis | Smoking prevalence | None | 3 | 1 |
| Multiple sclerosis | Socio-demographic Index | None | 3 | 1 |
| **GBD: Global Burden of Disease; CODEm: Cause of Death Ensemble Modeling; LDI: Lag Distributed Income** | | | | |
