# Supplementary material for: Global burden of multiple sclerosis and its attributable risk factors, 1990–2019
Source: Front Neurol. 2024 Oct 25;15:1448377. doi: 10.3389/fneur.2024.1448377 (PMC11545682; doi:10.3389/fneur.2024.1448377)
Supplement: Supplementary file 18 [file Table_2.DOC]

| **Table S2: Prevalent cases of multiple sclerosis in 1990 and 2019 and the percentage change in the age-standardised rates (ASRs) per 100,000, by location**  **(Generated from data available from http://ghdx.healthdata.org/gbd-results-tool)** | | | | | |
| --- | --- | --- | --- | --- | --- |
|  | **1990** | | **2019** | | **Percentage change in ASRs per 100,000** |
|  | **No (95% UI)** | **ASRs per 100,000 (95% UI)** | **No (95% UI)** | **ASRs per 100,000 (95% UI)** |
| **Global** | **1022937 (874531 , 1181199)** | **22.7 (19.4 , 26)** | **1756792 (1531919 , 1973623)** | **21.3 (18.5 , 23.9)** | **-6.2 (-8.7 , -3.8)** |
| **High-income North America** | **310981 (271151 , 357215)** | **98.4 (85.8 , 112.9)** | **476332 (438853 , 513566)** | **103.8 (95.4 , 112.1)** | **5.5 (-4.6 , 14.5)** |
| **Canada** | **35112 (34202 , 36027)** | **112.6 (109.7 , 115.5)** | **67054 (65284 , 69010)** | **136.8 (133 , 140.9)** | **21.5 (18.8 , 24.5)** |
| **Greenland** | **42 (34 , 50)** | **72 (58.4 , 87.2)** | **53 (43 , 64)** | **79.7 (64.1 , 97.2)** | **10.7 (4.4 , 19.1)** |
| **United States of America** | **275820 (236086 , 321944)** | **96.8 (82.9 , 112.9)** | **409217 (373509 , 445047)** | **99.9 (90.9 , 108.9)** | **3.3 (-7.8 , 13.3)** |
| **Australasia** | **8875 (7746 , 9953)** | **39.8 (34.8 , 44.6)** | **21100 (18034 , 23970)** | **57 (48.4 , 65.4)** | **43.2 (30.8 , 55.1)** |
| **Australia** | **7554 (6651 , 8384)** | **40.5 (35.7 , 45)** | **19181 (16305 , 21823)** | **61.1 (51.7 , 70.1)** | **50.7 (36.6 , 64.6)** |
| **New Zealand** | **1321 (1097 , 1540)** | **36.2 (30.1 , 42.2)** | **1920 (1610 , 2223)** | **34.2 (28.4 , 40.1)** | **-5.6 (-9.7 , -1.5)** |
| **High-income Asia Pacific** | **15514 (12179 , 19192)** | **7.7 (6.1 , 9.5)** | **24006 (19021 , 29147)** | **8.6 (6.8 , 10.5)** | **11.4 (9.6 , 13.1)** |
| **Brunei Darussalam** | **6 (4 , 7)** | **3 (2.3 , 3.7)** | **15 (11 , 19)** | **3.2 (2.4 , 4)** | **6.9 (2.3 , 12)** |
| **Japan** | **11914 (9365 , 14675)** | **7.6 (6 , 9.4)** | **17142 (13660 , 20737)** | **8.8 (7 , 10.8)** | **15.7 (13.7 , 17.5)** |
| **Singapore** | **88 (66 , 108)** | **2.8 (2.2 , 3.4)** | **225 (170 , 274)** | **2.9 (2.2 , 3.5)** | **2 (-1.6 , 6)** |
| **Republic of Korea** | **3506 (2704 , 4386)** | **8.4 (6.6 , 10.4)** | **6624 (5186 , 8107)** | **8.7 (6.8 , 10.7)** | **3.7 (-0.2 , 8.1)** |
| **Western Europe** | **321474 (281169 , 368085)** | **69.4 (60.6 , 79.5)** | **522842 (459258 , 590605)** | **88.5 (77.7 , 100.7)** | **27.6 (24.7 , 30.6)** |
| **Andorra** | **33 (28 , 39)** | **52.2 (44.6 , 61)** | **73 (63 , 84)** | **60.6 (52 , 70.7)** | **16.2 (9.9 , 22.5)** |
| **Austria** | **5544 (4746 , 6525)** | **58.9 (50.3 , 69.3)** | **9449 (8193 , 10943)** | **77.5 (66.9 , 90.5)** | **31.6 (23.3 , 41.4)** |
| **Belgium** | **7913 (6936 , 8893)** | **65 (56.9 , 73.3)** | **11050 (9458 , 13038)** | **73.3 (62.6 , 86.9)** | **12.8 (4 , 24)** |
| **Cyprus** | **260 (228 , 293)** | **31.7 (27.9 , 35.8)** | **723 (605 , 863)** | **42.3 (35.4 , 50.6)** | **33.4 (22.6 , 46.6)** |
| **Denmark** | **6592 (6000 , 7352)** | **104.9 (94.7 , 117.3)** | **8777 (7729 , 9975)** | **117.5 (103.4 , 135.9)** | **12 (4.4 , 21.7)** |
| **Finland** | **4161 (3763 , 4579)** | **67.6 (60.8 , 74.3)** | **5981 (5444 , 6558)** | **79.6 (72.4 , 87.6)** | **17.8 (12.6 , 23.6)** |
| **France** | **44786 (37786 , 53347)** | **66.8 (56.5 , 79.4)** | **77061 (68163 , 86632)** | **91.3 (80.6 , 102.9)** | **36.7 (22.7 , 50)** |
| **Germany** | **74306 (64670 , 86571)** | **73.3 (63.9 , 85.7)** | **101390 (88383 , 118804)** | **85.8 (74.9 , 101)** | **17.1 (9.6 , 25.2)** |
| **Greece** | **3082 (2652 , 3516)** | **25 (21.4 , 28.6)** | **4783 (4023 , 5613)** | **33.9 (28.2 , 40.1)** | **35.7 (28.3 , 45.7)** |
| **Iceland** | **266 (252 , 281)** | **101.5 (95.7 , 107)** | **450 (385 , 532)** | **105.8 (90.4 , 125.1)** | **4.2 (-9 , 20.3)** |
| **Ireland** | **4253 (3708 , 4831)** | **117.2 (102.1 , 133.3)** | **7646 (6455 , 8975)** | **126.1 (106.8 , 149.5)** | **7.6 (-1.5 , 18.6)** |
| **Israel** | **1394 (1136 , 1673)** | **30.1 (24.5 , 36)** | **3305 (2699 , 3944)** | **33.9 (27.7 , 40.6)** | **12.8 (8.4 , 18.2)** |
| **Italy** | **45114 (38270 , 52987)** | **64 (54.2 , 75.4)** | **73486 (61962 , 86509)** | **85.6 (71.7 , 102.2)** | **33.8 (30.7 , 38.4)** |
| **Luxembourg** | **367 (322 , 424)** | **77.5 (68.2 , 89.7)** | **700 (605 , 819)** | **85.5 (73.9 , 100.7)** | **10.3 (3.8 , 18)** |
| **Malta** | **74 (63 , 85)** | **17.8 (15.2 , 20.5)** | **145 (119 , 170)** | **24.4 (20 , 28.7)** | **36.7 (27.4 , 49.8)** |
| **Monaco** | **20 (17 , 24)** | **47.2 (39.8 , 56.2)** | **31 (26 , 36)** | **57.4 (48.4 , 66.8)** | **21.5 (14.1 , 29.1)** |
| **Netherlands** | **14484 (12707 , 17005)** | **82.2 (72 , 96.5)** | **20286 (17360 , 23646)** | **88.9 (76.3 , 104)** | **8.2 (1.5 , 15.2)** |
| **Norway** | **4409 (3754 , 5105)** | **87.9 (74.9 , 101.8)** | **9787 (8254 , 11454)** | **139.2 (117 , 163.8)** | **58.3 (52.5 , 64.2)** |
| **Portugal** | **3572 (2927 , 4202)** | **30.7 (25 , 36.1)** | **4678 (4281 , 5077)** | **30.6 (27.9 , 33.4)** | **-0.4 (-11.9 , 15)** |
| **San Marino** | **10 (8 , 12)** | **37.1 (29.7 , 45.2)** | **18 (15 , 22)** | **41.4 (33.1 , 50.4)** | **11.5 (7.1 , 16)** |
| **Spain** | **23156 (20563 , 26095)** | **52 (46.3 , 58.5)** | **50145 (45971 , 54458)** | **77.4 (70.8 , 84.4)** | **48.7 (37.6 , 59.4)** |
| **Sweden** | **14066 (12121 , 16038)** | **128.7 (111.3 , 146.9)** | **20971 (18290 , 23762)** | **152.2 (132.1 , 174)** | **18.2 (14.1 , 22.5)** |
| **Switzerland** | **7309 (6346 , 8555)** | **85.7 (74.4 , 100.4)** | **10991 (9637 , 12588)** | **91.4 (79.7 , 104.8)** | **6.6 (-0.6 , 14.8)** |
| **United Kingdom** | **56035 (48903 , 63930)** | **81.2 (70.3 , 93)** | **100461 (87360 , 115279)** | **114.2 (98.4 , 132.1)** | **40.7 (36.1 , 45)** |
| **Southern Latin America** | **10611 (8624 , 12647)** | **22.4 (18.2 , 26.7)** | **17804 (14604 , 21088)** | **23.6 (19.3 , 28.1)** | **5.3 (2.3 , 8.4)** |
| **Argentina** | **7528 (6200 , 8935)** | **23.5 (19.3 , 27.9)** | **12208 (10098 , 14507)** | **24.7 (20.4 , 29.4)** | **4.9 (0.7 , 9.6)** |
| **Chile** | **2336 (1841 , 2847)** | **19.5 (15.5 , 23.6)** | **4638 (3680 , 5625)** | **21.3 (16.8 , 25.9)** | **9 (5.6 , 13.1)** |
| **Uruguay** | **747 (614 , 883)** | **22.5 (18.5 , 26.7)** | **958 (789 , 1130)** | **23.6 (19.3 , 28)** | **5 (1 , 8.8)** |
| **Eastern Europe** | **65985 (55075 , 77618)** | **25.9 (21.6 , 30.5)** | **57320 (47592 , 67240)** | **22 (18.2 , 26)** | **-15 (-16.9 , -13.3)** |
| **Belarus** | **2300 (1876 , 2726)** | **19.7 (16.1 , 23.4)** | **2416 (1970 , 2864)** | **20 (16.2 , 24)** | **1.6 (-2.5 , 5.7)** |
| **Estonia** | **447 (377 , 522)** | **25.4 (21.4 , 29.9)** | **381 (324 , 446)** | **23.8 (20.2 , 27.9)** | **-6.5 (-10.8 , -1.2)** |
| **Latvia** | **792 (674 , 918)** | **26.5 (22.5 , 30.8)** | **602 (510 , 696)** | **26 (21.9 , 30.2)** | **-2 (-6.3 , 2.8)** |
| **Lithuania** | **1002 (841 , 1161)** | **24.9 (20.9 , 29)** | **831 (698 , 970)** | **24.4 (20.4 , 28.7)** | **-1.7 (-6.6 , 2.8)** |
| **Republic of Moldova** | **613 (482 , 771)** | **13.3 (10.5 , 16.7)** | **694 (584 , 813)** | **14.9 (12.5 , 17.5)** | **12.3 (0.9 , 23.3)** |
| **Russian Federation** | **38710 (31881 , 45698)** | **22.9 (18.8 , 27.1)** | **35369 (29007 , 41794)** | **19.6 (16 , 23.4)** | **-14.2 (-16 , -12.6)** |
| **Ukraine** | **22122 (18653 , 26025)** | **36.5 (30.7 , 43.2)** | **17027 (14360 , 19869)** | **30.3 (25.4 , 35.7)** | **-16.8 (-20.6 , -13.2)** |
| **Central Europe** | **51933 (45283 , 59032)** | **37.7 (32.7 , 43)** | **60506 (53805 , 67750)** | **41 (36.3 , 46.2)** | **8.8 (5.1 , 13.3)** |
| **Albania** | **1991 (1784 , 2213)** | **81.3 (73.3 , 89.3)** | **2529 (2302 , 2762)** | **69.8 (62.6 , 76.9)** | **-14.2 (-20.5 , -7.3)** |
| **Bosnia and Herzegovina** | **1204 (1012 , 1405)** | **24.8 (20.9 , 28.9)** | **1167 (1035 , 1298)** | **26.2 (23.1 , 29.4)** | **5.7 (-1 , 13)** |
| **Bulgaria** | **4791 (4270 , 5305)** | **42.4 (37.5 , 47.4)** | **4188 (3745 , 4646)** | **41.2 (36.2 , 46.5)** | **-2.9 (-8.4 , 3.5)** |
| **Croatia** | **1170 (987 , 1345)** | **19.9 (16.8 , 23)** | **1504 (1292 , 1729)** | **26.6 (22.6 , 30.8)** | **33.7 (28 , 41.7)** |
| **Czechia** | **3808 (3283 , 4343)** | **32 (27.4 , 36.8)** | **4367 (3768 , 5025)** | **31.5 (27 , 36.7)** | **-1.5 (-6.3 , 4.3)** |
| **Hungary** | **5680 (5266 , 6141)** | **45.4 (42 , 49.3)** | **4417 (3808 , 5106)** | **35.1 (30.2 , 40.7)** | **-22.7 (-30.2 , -14.7)** |
| **Montenegro** | **274 (239 , 310)** | **41.9 (36.5 , 47.4)** | **371 (332 , 419)** | **46.3 (40.9 , 52.8)** | **10.4 (5.9 , 15.1)** |
| **North Macedonia** | **594 (500 , 692)** | **28.6 (24.1 , 33.3)** | **1057 (917 , 1208)** | **37.1 (32 , 42.9)** | **29.9 (23.9 , 37)** |
| **Poland** | **22420 (19055 , 26005)** | **53.8 (45.7 , 62.5)** | **28710 (25552 , 32264)** | **58.7 (52.3 , 65.8)** | **9 (1.2 , 18)** |
| **Romania** | **4119 (3393 , 4865)** | **16.1 (13.2 , 19.1)** | **3983 (3273 , 4730)** | **16.3 (13.3 , 19.3)** | **1.1 (-3.4 , 6.1)** |
| **Serbia** | **3816 (3374 , 4302)** | **35.2 (31 , 40)** | **5234 (4629 , 5823)** | **48.5 (42.1 , 54.2)** | **37.7 (28.4 , 49.2)** |
| **Slovakia** | **1270 (1049 , 1491)** | **22.5 (18.6 , 26.5)** | **1873 (1580 , 2178)** | **26.6 (22.2 , 31.2)** | **18.1 (13.5 , 23.3)** |
| **Slovenia** | **796 (692 , 909)** | **34.7 (30 , 39.8)** | **1104 (980 , 1247)** | **39.1 (34.2 , 44.8)** | **12.6 (8.2 , 17.4)** |
| **Central Asia** | **17259 (14851 , 19657)** | **32.8 (28.4 , 37.1)** | **28520 (24097 , 32799)** | **32.1 (27.4 , 36.9)** | **-2 (-5.4 , 1.8)** |
| **Armenia** | **777 (654 , 901)** | **25.2 (21.3 , 29.1)** | **1268 (1087 , 1450)** | **33.1 (28.2 , 37.8)** | **31.4 (24 , 39.9)** |
| **Azerbaijan** | **1095 (881 , 1313)** | **18.4 (14.9 , 21.9)** | **2378 (1932 , 2818)** | **20.7 (16.9 , 24.5)** | **12.6 (8.1 , 16.8)** |
| **Georgia** | **1234 (1001 , 1471)** | **20.3 (16.4 , 24.3)** | **1258 (1041 , 1469)** | **25.8 (21.3 , 30.1)** | **26.9 (19.2 , 35.4)** |
| **Kazakhstan** | **8219 (7247 , 9142)** | **58.7 (51.9 , 65.3)** | **11823 (10369 , 13332)** | **61.6 (54.1 , 69.3)** | **4.9 (-1.1 , 12.1)** |
| **Kyrgyzstan** | **633 (511 , 763)** | **18.7 (15.1 , 22.3)** | **1142 (912 , 1374)** | **19.7 (15.8 , 23.7)** | **5.8 (0.9 , 10.9)** |
| **Mongolia** | **292 (231 , 358)** | **21.3 (17.1 , 25.8)** | **754 (603 , 907)** | **23 (18.5 , 27.4)** | **7.9 (2.5 , 13.5)** |
| **Tajikistan** | **571 (461 , 697)** | **16.8 (13.6 , 20.2)** | **1389 (1111 , 1688)** | **18.3 (14.8 , 22.1)** | **9 (3.5 , 14.4)** |
| **Turkmenistan** | **759 (658 , 866)** | **33.1 (29 , 37.3)** | **1659 (1445 , 1872)** | **35.2 (30.9 , 39.7)** | **6.4 (1.7 , 12.5)** |
| **Uzbekistan** | **3679 (3088 , 4279)** | **27.3 (23.2 , 31.7)** | **6850 (5565 , 8246)** | **22.9 (18.8 , 27.3)** | **-16.2 (-21.5 , -10)** |
| **Central Latin America** | **7221 (5614 , 8902)** | **6.2 (4.9 , 7.5)** | **21533 (17150 , 25931)** | **8.4 (6.7 , 10.2)** | **36.2 (32.9 , 40.5)** |
| **Colombia** | **920 (701 , 1150)** | **3.7 (2.9 , 4.6)** | **2483 (1912 , 3062)** | **4.8 (3.7 , 5.9)** | **28.6 (21.7 , 36.2)** |
| **Costa Rica** | **133 (102 , 164)** | **5.9 (4.6 , 7.2)** | **389 (305 , 467)** | **7.4 (5.8 , 8.9)** | **25.2 (18 , 31.9)** |
| **El Salvador** | **168 (130 , 213)** | **4.6 (3.5 , 5.7)** | **351 (273 , 432)** | **5.9 (4.5 , 7.2)** | **28.5 (22.6 , 34.8)** |
| **Guatemala** | **246 (188 , 307)** | **4.7 (3.7 , 5.8)** | **925 (709 , 1157)** | **6.5 (5 , 7.9)** | **37.3 (31.2 , 43.8)** |
| **Honduras** | **135 (103 , 170)** | **4.6 (3.6 , 5.7)** | **454 (347 , 569)** | **5.7 (4.4 , 7)** | **23.3 (17.4 , 29.3)** |
| **Mexico** | **4799 (3753 , 5921)** | **8 (6.4 , 9.7)** | **14602 (11682 , 17523)** | **11.3 (9.1 , 13.5)** | **40.7 (36.7 , 45.7)** |
| **Nicaragua** | **106 (81 , 134)** | **4.7 (3.6 , 5.7)** | **338 (261 , 420)** | **5.9 (4.6 , 7.2)** | **26.1 (19.3 , 33.5)** |
| **Panama** | **83 (65 , 102)** | **4.5 (3.5 , 5.4)** | **218 (174 , 261)** | **5.2 (4.1 , 6.2)** | **16.2 (10.2 , 22.8)** |
| **Venezuela (Bolivarian Republic of)** | **630 (483 , 782)** | **4.6 (3.5 , 5.6)** | **1773 (1383 , 2156)** | **5.8 (4.5 , 7)** | **26.3 (20.3 , 31.8)** |
| **Andean Latin America** | **1394 (1076 , 1733)** | **5.1 (4 , 6.3)** | **4177 (3242 , 5078)** | **6.8 (5.3 , 8.3)** | **32.9 (28 , 38.3)** |
| **Bolivia (Plurinational State of)** | **297 (229 , 371)** | **6.7 (5.3 , 8.3)** | **884 (686 , 1087)** | **8.4 (6.5 , 10.3)** | **24.3 (19 , 30)** |
| **Ecuador** | **285 (217 , 357)** | **4 (3.1 , 4.9)** | **876 (670 , 1075)** | **5.3 (4 , 6.4)** | **32.6 (25.7 , 39.6)** |
| **Peru** | **812 (623 , 1010)** | **5.2 (4 , 6.4)** | **2417 (1881 , 2924)** | **7.1 (5.5 , 8.6)** | **36.1 (28.9 , 43.9)** |
| **Caribbean** | **2679 (2090 , 3262)** | **9 (7.1 , 10.8)** | **5285 (4204 , 6317)** | **10.4 (8.3 , 12.5)** | **16.4 (13.8 , 19.6)** |
| **Antigua and Barbuda** | **7 (5 , 8)** | **12.7 (10.4 , 15)** | **18 (15 , 21)** | **17.1 (14.2 , 19.9)** | **34.1 (26 , 43.7)** |
| **Barbados** | **30 (24 , 36)** | **11.9 (9.6 , 14.3)** | **62 (52 , 72)** | **15.8 (13.1 , 18.7)** | **33.3 (24.5 , 42.2)** |
| **Belize** | **8 (6 , 10)** | **6.8 (5.3 , 8.4)** | **29 (23 , 37)** | **8 (6.3 , 9.9)** | **17.2 (12.8 , 23.4)** |
| **Bermuda** | **10 (8 , 12)** | **14 (11.2 , 16.8)** | **15 (12 , 18)** | **15.9 (12.7 , 19.1)** | **13.4 (8.6 , 18.5)** |
| **Bahamas** | **22 (17 , 28)** | **10.4 (8.2 , 12.6)** | **53 (42 , 64)** | **12.3 (9.7 , 14.8)** | **18.4 (13.6 , 23.7)** |
| **Cuba** | **1167 (927 , 1409)** | **10.8 (8.6 , 13)** | **1988 (1608 , 2357)** | **13 (10.4 , 15.6)** | **19.7 (14.4 , 25.8)** |
| **Dominica** | **4 (3 , 5)** | **6.9 (5.4 , 8.4)** | **6 (5 , 7)** | **7.6 (6 , 9.2)** | **10.6 (5.4 , 15.6)** |
| **Dominican Republic** | **372 (285 , 468)** | **7.1 (5.5 , 8.9)** | **888 (685 , 1092)** | **8.4 (6.5 , 10.3)** | **17.7 (12.4 , 23.1)** |
| **Grenada** | **6 (5 , 7)** | **9.1 (7.2 , 11.1)** | **13 (10 , 15)** | **10.9 (8.7 , 13.1)** | **19.2 (14.1 , 26.3)** |
| **Guyana** | **26 (20 , 34)** | **4.5 (3.5 , 5.7)** | **40 (31 , 50)** | **5.4 (4.1 , 6.6)** | **17.9 (12.2 , 25.5)** |
| **Haiti** | **357 (276 , 441)** | **7.7 (6 , 9.4)** | **988 (759 , 1211)** | **9.3 (7.3 , 11.3)** | **21.3 (15.5 , 27.7)** |
| **Jamaica** | **134 (104 , 169)** | **7.3 (5.7 , 9.1)** | **251 (196 , 310)** | **8.3 (6.5 , 10.3)** | **14 (7.9 , 20.1)** |
| **Puerto Rico** | **345 (271 , 418)** | **9.6 (7.6 , 11.6)** | **546 (436 , 655)** | **11.7 (9.3 , 14.1)** | **21.8 (16.7 , 27.4)** |
| **Saint Kitts and Nevis** | **5 (4 , 6)** | **14.4 (11.8 , 17.1)** | **12 (10 , 14)** | **16.1 (13.3 , 19)** | **11.8 (6 , 17.4)** |
| **Saint Lucia** | **7 (5 , 9)** | **6.9 (5.4 , 8.5)** | **17 (13 , 21)** | **8 (6.2 , 9.8)** | **15.6 (10.2 , 20.8)** |
| **Saint Vincent and the Grenadines** | **5 (4 , 7)** | **6.7 (5.2 , 8.2)** | **10 (8 , 12)** | **7.8 (6.1 , 9.5)** | **15.7 (10.6 , 21.3)** |
| **Suriname** | **15 (11 , 19)** | **4.7 (3.5 , 5.8)** | **35 (27 , 42)** | **5.5 (4.3 , 6.8)** | **18.6 (13 , 23.8)** |
| **Trinidad and Tobago** | **60 (46 , 75)** | **5.8 (4.5 , 7.2)** | **119 (94 , 147)** | **7 (5.5 , 8.6)** | **20.3 (13.8 , 26.5)** |
| **United States Virgin Islands** | **10 (7 , 11)** | **9 (7.1 , 10.9)** | **15 (12 , 17)** | **10.8 (8.6 , 12.9)** | **19.7 (13.9 , 25.7)** |
| **Tropical Latin America** | **18819 (15190 , 22518)** | **16.3 (13.2 , 19.3)** | **47516 (38255 , 56927)** | **18.9 (15.2 , 22.6)** | **16 (13.3 , 18.9)** |
| **Brazil** | **18445 (14894 , 22086)** | **16.3 (13.3 , 19.3)** | **46575 (37515 , 55717)** | **19 (15.3 , 22.7)** | **16.2 (13.4 , 19.1)** |
| **Paraguay** | **374 (294 , 458)** | **13.4 (10.5 , 16.2)** | **941 (735 , 1145)** | **14.7 (11.5 , 17.8)** | **9.9 (5.8 , 16.3)** |
| **East Asia** | **22918 (17390 , 29272)** | **1.9 (1.5 , 2.4)** | **45850 (35804 , 57092)** | **2.4 (1.9 , 3)** | **25.4 (22.8 , 28.1)** |
| **China** | **21609 (16325 , 27657)** | **1.9 (1.4 , 2.4)** | **42571 (33001 , 53329)** | **2.3 (1.8 , 2.9)** | **23.3 (20.5 , 25.9)** |
| **Democratic People's Republic of Korea** | **845 (655 , 1059)** | **4.1 (3.2 , 5.2)** | **1339 (1047 , 1663)** | **4.3 (3.3 , 5.3)** | **3.4 (-1.1 , 8.1)** |
| **Taiwan (Province of China)** | **465 (396 , 536)** | **2.3 (2 , 2.6)** | **1940 (1637 , 2282)** | **6.2 (5.2 , 7.4)** | **169.4 (138.8 , 206.2)** |
| **Southeast Asia** | **9292 (6963 , 11891)** | **2.4 (1.9 , 3.1)** | **18584 (14181 , 23400)** | **2.6 (2 , 3.2)** | **5.7 (4.2 , 7.1)** |
| **Cambodia** | **162 (121 , 206)** | **2.2 (1.7 , 2.8)** | **377 (285 , 477)** | **2.4 (1.8 , 3)** | **6.3 (2.3 , 10.8)** |
| **Indonesia** | **3246 (2430 , 4183)** | **2.1 (1.6 , 2.7)** | **6172 (4671 , 7803)** | **2.2 (1.7 , 2.8)** | **4.6 (3 , 6.4)** |
| **Lao People's Democratic Republic** | **81 (61 , 103)** | **2.6 (2 , 3.3)** | **186 (140 , 238)** | **2.8 (2.1 , 3.5)** | **5.2 (0.9 , 9.9)** |
| **Malaysia** | **254 (190 , 325)** | **1.8 (1.3 , 2.2)** | **609 (464 , 764)** | **1.9 (1.4 , 2.3)** | **6.1 (1.5 , 11.1)** |
| **Maldives** | **2 (2 , 3)** | **1.7 (1.3 , 2.1)** | **9 (7 , 12)** | **1.7 (1.3 , 2.2)** | **2.5 (-3.3 , 6.2)** |
| **Mauritius** | **32 (24 , 40)** | **3.1 (2.4 , 3.9)** | **51 (40 , 64)** | **3.2 (2.5 , 4.1)** | **4.9 (0.3 , 8.7)** |
| **Myanmar** | **1066 (818 , 1349)** | **3.2 (2.5 , 4)** | **1886 (1451 , 2348)** | **3.4 (2.6 , 4.2)** | **5.4 (1.1 , 10.8)** |
| **Philippines** | **1245 (929 , 1591)** | **2.6 (2 , 3.3)** | **2840 (2161 , 3600)** | **2.7 (2.1 , 3.4)** | **5.7 (4.7 , 6.6)** |
| **Sri Lanka** | **345 (260 , 436)** | **2.2 (1.7 , 2.8)** | **584 (450 , 730)** | **2.4 (1.8 , 3)** | **6 (1.8 , 10.4)** |
| **Seychelles** | **2 (1 , 2)** | **2.6 (2 , 3.2)** | **3 (3 , 4)** | **2.8 (2.2 , 3.4)** | **8.2 (2.7 , 12.8)** |
| **Thailand** | **1379 (1033 , 1781)** | **2.6 (2 , 3.3)** | **2536 (1974 , 3171)** | **2.8 (2.1 , 3.5)** | **4.8 (0.8 , 9.1)** |
| **Timor-Leste** | **11 (8 , 14)** | **1.9 (1.4 , 2.4)** | **21 (16 , 27)** | **2 (1.5 , 2.6)** | **7.7 (2.3 , 13.2)** |
| **Viet Nam** | **1455 (1099 , 1858)** | **2.8 (2.1 , 3.5)** | **3285 (2500 , 4124)** | **3 (2.3 , 3.7)** | **6.3 (2.1 , 10.5)** |
| **Oceania** | **89 (66 , 115)** | **1.8 (1.3 , 2.2)** | **201 (150 , 258)** | **1.7 (1.3 , 2.2)** | **-1 (-3.7 , 2)** |
| **American Samoa** | **1 (1 , 1)** | **2.2 (1.7 , 2.8)** | **1 (1 , 2)** | **2.3 (1.8 , 2.9)** | **4.2 (-0.6 , 8.3)** |
| **Cook Islands** | **0 (0 , 1)** | **2.8 (2.1 , 3.5)** | **1 (0 , 1)** | **2.9 (2.3 , 3.7)** | **5.7 (1.9 , 10)** |
| **Micronesia (Federated States of)** | **1 (1 , 2)** | **1.7 (1.3 , 2.2)** | **2 (1 , 2)** | **1.8 (1.4 , 2.3)** | **4.7 (0.2 , 8.8)** |
| **Fiji** | **15 (11 , 20)** | **2.4 (1.8 , 3)** | **23 (17 , 29)** | **2.5 (1.9 , 3.1)** | **2.6 (-1.1 , 7.1)** |
| **Guam** | **3 (2 , 4)** | **2.2 (1.7 , 2.8)** | **4 (3 , 5)** | **2.3 (1.7 , 2.9)** | **2.5 (-1.8 , 7.4)** |
| **Kiribati** | **1 (1 , 1)** | **1.6 (1.2 , 2)** | **2 (1 , 2)** | **1.6 (1.2 , 2)** | **2.4 (-3.4 , 9.3)** |
| **Marshall Islands** | **1 (0 , 1)** | **1.8 (1.4 , 2.3)** | **1 (1 , 1)** | **1.9 (1.4 , 2.4)** | **3 (-1.3 , 7.9)** |
| **Nauru** | **0 (0 , 0)** | **1.4 (1 , 1.7)** | **0 (0 , 0)** | **1.4 (1.1 , 1.8)** | **5.9 (0.9 , 11.2)** |
| **Niue** | **0 (0 , 0)** | **2.7 (2.1 , 3.4)** | **0 (0 , 0)** | **2.8 (2.1 , 3.5)** | **4.2 (-0.2 , 8.1)** |
| **Northern Mariana Islands** | **1 (1 , 1)** | **2.4 (1.9 , 3.1)** | **1 (1 , 2)** | **2.6 (2 , 3.2)** | **5.2 (1.5 , 8.9)** |
| **Palau** | **0 (0 , 0)** | **1.8 (1.4 , 2.3)** | **0 (0 , 1)** | **1.8 (1.4 , 2.3)** | **2.8 (-1.1 , 6.5)** |
| **Papua New Guinea** | **49 (36 , 64)** | **1.5 (1.2 , 2)** | **134 (99 , 173)** | **1.6 (1.2 , 2)** | **2.5 (-1.6 , 7.1)** |
| **Samoa** | **3 (2 , 3)** | **2.2 (1.7 , 2.8)** | **4 (3 , 5)** | **2.3 (1.7 , 2.8)** | **2.5 (-1.5 , 6.4)** |
| **Solomon Islands** | **4 (3 , 5)** | **1.8 (1.3 , 2.3)** | **10 (8 , 13)** | **1.9 (1.4 , 2.3)** | **4.1 (-0.1 , 9.1)** |
| **Tokelau** | **0 (0 , 0)** | **1.8 (1.4 , 2.3)** | **0 (0 , 0)** | **1.9 (1.4 , 2.4)** | **3.4 (-0.7 , 8.9)** |
| **Tonga** | **2 (1 , 2)** | **2.8 (2.1 , 3.5)** | **3 (2 , 3)** | **2.8 (2.2 , 3.6)** | **2.6 (-1 , 6.8)** |
| **Tuvalu** | **0 (0 , 0)** | **1.7 (1.3 , 2.1)** | **0 (0 , 0)** | **1.7 (1.3 , 2.1)** | **1.3 (-3.2 , 6.3)** |
| **Vanuatu** | **2 (2 , 3)** | **2.2 (1.7 , 2.8)** | **6 (4 , 7)** | **2.3 (1.8 , 2.9)** | **2.9 (-1.3 , 6.9)** |
| **North Africa and Middle East** | **82071 (70381 , 94702)** | **35 (30.1 , 39.9)** | **222696 (190733 , 256781)** | **39 (33.6 , 44.7)** | **11.5 (10 , 12.8)** |
| **Afghanistan** | **2778 (2305 , 3290)** | **35.9 (29.6 , 42.8)** | **9996 (8248 , 12114)** | **43.1 (36 , 51.2)** | **19.9 (13.6 , 25.7)** |
| **Algeria** | **5451 (4440 , 6681)** | **33.2 (27.1 , 40)** | **17581 (14373 , 21340)** | **41.9 (34.2 , 50.6)** | **26.4 (20.4 , 33.6)** |
| **Bahrain** | **101 (79 , 128)** | **25.3 (20.1 , 30.8)** | **592 (471 , 734)** | **32.7 (26.3 , 40.3)** | **29.1 (21.6 , 36.4)** |
| **Egypt** | **6831 (5352 , 8364)** | **16.8 (13.3 , 20.5)** | **17764 (14045 , 21567)** | **20.6 (16.4 , 25)** | **22.2 (17.7 , 27.4)** |
| **Iran (Islamic Republic of)** | **19921 (16655 , 23710)** | **53.9 (45.2 , 63.2)** | **46854 (39948 , 53742)** | **51.2 (44.1 , 58.5)** | **-4.9 (-8.2 , -1.6)** |
| **Iraq** | **3420 (2704 , 4216)** | **31.6 (25.1 , 38.8)** | **12797 (10065 , 16073)** | **37.8 (30 , 46.9)** | **19.5 (14.2 , 25.2)** |
| **Jordan** | **1035 (898 , 1172)** | **47.6 (41.1 , 53.5)** | **4613 (3653 , 5615)** | **47 (37.9 , 56.8)** | **-1.2 (-13.3 , 10.6)** |
| **Kuwait** | **464 (374 , 568)** | **33.2 (27.4 , 39.8)** | **2717 (2228 , 3296)** | **53 (43.7 , 63.5)** | **59.7 (50.5 , 69.9)** |
| **Lebanon** | **1045 (845 , 1265)** | **40 (32.5 , 48.5)** | **2918 (2372 , 3504)** | **53.9 (43.9 , 64.2)** | **34.7 (27.2 , 43.9)** |
| **Libya** | **823 (663 , 1000)** | **31.2 (25.3 , 37.6)** | **2974 (2455 , 3556)** | **41.1 (34.2 , 48.9)** | **31.6 (24.9 , 38.4)** |
| **Morocco** | **5962 (4787 , 7237)** | **32.3 (26.3 , 38.8)** | **15001 (12205 , 18295)** | **40.9 (33.3 , 49.7)** | **26.4 (19.3 , 34.6)** |
| **Palestine** | **407 (325 , 502)** | **35 (28.1 , 42.6)** | **1498 (1205 , 1827)** | **41.5 (33.6 , 50.2)** | **18.5 (13.9 , 24.3)** |
| **Oman** | **328 (262 , 401)** | **25.6 (20.7 , 30.8)** | **1483 (1206 , 1835)** | **34.3 (28.3 , 41)** | **33.8 (27.2 , 41.9)** |
| **Qatar** | **139 (108 , 175)** | **36.4 (28.9 , 44.6)** | **1784 (1551 , 2001)** | **56.1 (49.2 , 62.9)** | **54.1 (34 , 77.5)** |
| **Saudi Arabia** | **2314 (1800 , 2877)** | **21.9 (17.2 , 26.7)** | **10757 (8342 , 13614)** | **28.4 (22.4 , 35.2)** | **29.7 (23.9 , 35.9)** |
| **Sudan** | **2398 (1877 , 2955)** | **18.6 (14.6 , 22.7)** | **6715 (5309 , 8260)** | **23.1 (18.5 , 27.9)** | **23.9 (18.2 , 30.7)** |
| **Syrian Arab Republic** | **2559 (1999 , 3179)** | **34 (26.8 , 41.7)** | **5899 (4683 , 7200)** | **42.1 (33.4 , 51.5)** | **23.8 (18.6 , 28.4)** |
| **Tunisia** | **2430 (1984 , 2977)** | **38.7 (31.7 , 46.9)** | **6567 (5389 , 7931)** | **49.7 (40.8 , 59.8)** | **28.4 (22.4 , 34.2)** |
| **Turkey** | **21801 (20854 , 22771)** | **47.6 (45.5 , 49.7)** | **45814 (43903 , 47892)** | **48.7 (46.7 , 50.9)** | **2.5 (0.1 , 5)** |
| **United Arab Emirates** | **383 (308 , 474)** | **24.1 (19.9 , 28.6)** | **3103 (2586 , 3694)** | **23.8 (20.5 , 27.5)** | **-0.9 (-9.2 , 7.7)** |
| **Yemen** | **1427 (1112 , 1772)** | **19 (14.8 , 23.2)** | **5042 (4003 , 6214)** | **23.3 (18.6 , 28.4)** | **22.9 (16.8 , 29.2)** |
| **South Asia** | **58044 (45007 , 71723)** | **7 (5.5 , 8.5)** | **136608 (107613 , 167811)** | **8 (6.3 , 9.7)** | **14.3 (13 , 15.8)** |
| **Bangladesh** | **4893 (3763 , 6059)** | **6.9 (5.4 , 8.4)** | **11556 (9035 , 14342)** | **7.5 (5.9 , 9.2)** | **9.2 (5 , 13.7)** |
| **Bhutan** | **31 (24 , 40)** | **7.6 (6 , 9.6)** | **61 (48 , 76)** | **8.4 (6.6 , 10.3)** | **10.2 (4.8 , 15.8)** |
| **India** | **45485 (35340 , 56228)** | **6.8 (5.3 , 8.2)** | **106598 (83823 , 130270)** | **7.8 (6.2 , 9.5)** | **15.5 (13.9 , 17.2)** |
| **Nepal** | **1072 (838 , 1327)** | **7.8 (6.1 , 9.5)** | **2386 (1852 , 2934)** | **8.8 (6.9 , 10.8)** | **13.5 (8 , 18.4)** |
| **Pakistan** | **6563 (5143 , 8096)** | **8.7 (6.9 , 10.6)** | **16007 (12487 , 19819)** | **9.5 (7.5 , 11.6)** | **9.6 (7.2 , 12.3)** |
| **Southern Sub-Saharan Africa** | **2843 (2199 , 3569)** | **7.4 (5.8 , 9.1)** | **5667 (4417 , 7047)** | **7.8 (6.1 , 9.5)** | **5 (3.4 , 6.4)** |
| **Botswana** | **52 (39 , 66)** | **6.1 (4.7 , 7.6)** | **141 (109 , 178)** | **6.6 (5.1 , 8.2)** | **8.3 (4.1 , 13.4)** |
| **Lesotho** | **96 (75 , 120)** | **7.5 (5.9 , 9.2)** | **144 (111 , 180)** | **8 (6.2 , 9.7)** | **5.8 (1.5 , 10.3)** |
| **Namibia** | **58 (45 , 73)** | **6.1 (4.7 , 7.5)** | **127 (97 , 160)** | **6.5 (5 , 8)** | **7.1 (3.2 , 11.5)** |
| **South Africa** | **2256 (1751 , 2818)** | **7.9 (6.2 , 9.7)** | **4551 (3557 , 5626)** | **8.2 (6.5 , 10.1)** | **4.2 (2.4 , 5.5)** |
| **Eswatini** | **34 (26 , 43)** | **7 (5.5 , 8.7)** | **67 (52 , 85)** | **7.5 (5.8 , 9.3)** | **6.7 (2 , 11.8)** |
| **Zimbabwe** | **347 (263 , 444)** | **5.5 (4.2 , 6.9)** | **638 (487 , 818)** | **5.8 (4.5 , 7.2)** | **5.1 (0.8 , 10.3)** |
| **Western Sub-Saharan Africa** | **8225 (6460 , 10164)** | **6.5 (5.1 , 7.9)** | **23509 (18611 , 28685)** | **7.8 (6.3 , 9.2)** | **19.4 (16.7 , 22.8)** |
| **Benin** | **179 (137 , 230)** | **6.2 (4.8 , 7.7)** | **531 (407 , 674)** | **6.6 (5.2 , 8.1)** | **5.7 (1.3 , 10.6)** |
| **Burkina Faso** | **398 (304 , 502)** | **6.7 (5.2 , 8.3)** | **1045 (806 , 1321)** | **7.1 (5.5 , 8.7)** | **5.1 (-1.1 , 10.5)** |
| **Cameroon** | **376 (287 , 478)** | **5.6 (4.4 , 6.9)** | **1221 (928 , 1523)** | **6 (4.7 , 7.3)** | **7.7 (2.9 , 12.4)** |
| **Cabo Verde** | **17 (14 , 22)** | **7.8 (6.1 , 9.5)** | **44 (34 , 55)** | **8.2 (6.4 , 10.1)** | **5.7 (0.9 , 9.5)** |
| **Chad** | **269 (208 , 331)** | **7.2 (5.6 , 8.8)** | **699 (541 , 887)** | **7.5 (5.9 , 9.2)** | **3.3 (-1.8 , 8)** |
| **CÃ´te d'Ivoire** | **434 (333 , 554)** | **5.7 (4.4 , 7)** | **1171 (891 , 1487)** | **6.1 (4.7 , 7.5)** | **7.1 (3.3 , 11.8)** |
| **Gambia** | **40 (31 , 51)** | **6.7 (5.2 , 8.2)** | **116 (89 , 145)** | **7.5 (5.9 , 9.2)** | **12.7 (8.6 , 17.2)** |
| **Ghana** | **1131 (922 , 1347)** | **11.9 (9.9 , 13.9)** | **4586 (3965 , 5334)** | **19.3 (17 , 22.1)** | **62.5 (49.9 , 79.1)** |
| **Guinea** | **263 (206 , 330)** | **6.2 (4.9 , 7.7)** | **562 (435 , 709)** | **6.8 (5.3 , 8.4)** | **9 (4.2 , 13.5)** |
| **Guinea-Bissau** | **43 (33 , 55)** | **6.9 (5.3 , 8.5)** | **96 (74 , 121)** | **7.3 (5.7 , 8.9)** | **6.4 (1.1 , 11.2)** |
| **Liberia** | **71 (54 , 88)** | **5.2 (4 , 6.4)** | **201 (154 , 250)** | **5.6 (4.3 , 6.8)** | **7.9 (3.3 , 12.7)** |
| **Mali** | **441 (340 , 544)** | **7.7 (6.1 , 9.5)** | **1095 (852 , 1369)** | **8.1 (6.4 , 9.9)** | **5.5 (1.3 , 9.9)** |
| **Mauritania** | **124 (96 , 152)** | **9.1 (7.2 , 11)** | **284 (223 , 349)** | **9.8 (7.8 , 11.9)** | **7.4 (2.5 , 13)** |
| **Niger** | **359 (276 , 455)** | **7.5 (5.9 , 9.3)** | **1030 (796 , 1282)** | **8.1 (6.3 , 9.9)** | **7.5 (2.4 , 12.5)** |
| **Nigeria** | **3465 (2681 , 4350)** | **5.6 (4.3 , 6.9)** | **9219 (7099 , 11556)** | **6.4 (5 , 7.8)** | **14.8 (13.2 , 16.6)** |
| **Sao Tome and Principe** | **3 (3 , 4)** | **4.3 (3.3 , 5.4)** | **8 (6 , 9)** | **4.7 (3.6 , 5.8)** | **8.6 (3.1 , 13.4)** |
| **Senegal** | **339 (264 , 429)** | **7.2 (5.6 , 8.9)** | **853 (664 , 1068)** | **7.9 (6.2 , 9.7)** | **8.8 (4.5 , 13.7)** |
| **Sierra Leone** | **142 (109 , 178)** | **5.6 (4.3 , 6.9)** | **354 (270 , 453)** | **6.1 (4.7 , 7.5)** | **8.7 (4.4 , 13.6)** |
| **Togo** | **131 (100 , 167)** | **6.1 (4.7 , 7.5)** | **391 (300 , 486)** | **6.5 (5 , 8)** | **7.3 (3.1 , 13.8)** |
| **Eastern Sub-Saharan Africa** | **5338 (4060 , 6732)** | **4.5 (3.5 , 5.6)** | **13071 (9911 , 16699)** | **4.7 (3.7 , 5.9)** | **4.8 (3.7 , 6.1)** |
| **Burundi** | **130 (97 , 165)** | **3.7 (2.8 , 4.6)** | **293 (219 , 380)** | **3.7 (2.8 , 4.7)** | **0.5 (-3.7 , 5)** |
| **Comoros** | **16 (12 , 20)** | **5.2 (4 , 6.4)** | **34 (27 , 43)** | **5.5 (4.3 , 6.7)** | **5.5 (1.4 , 9.6)** |
| **Djibouti** | **15 (11 , 19)** | **5 (3.9 , 6.2)** | **55 (42 , 70)** | **5.3 (4.1 , 6.6)** | **5.5 (1.6 , 8.8)** |
| **Eritrea** | **108 (82 , 138)** | **5.7 (4.5 , 7.1)** | **296 (228 , 377)** | **6.1 (4.7 , 7.5)** | **6.2 (1.2 , 10.3)** |
| **Ethiopia** | **1476 (1125 , 1882)** | **4.6 (3.5 , 5.7)** | **3359 (2549 , 4288)** | **4.7 (3.7 , 5.9)** | **3.9 (1.3 , 6)** |
| **Kenya** | **503 (381 , 640)** | **3.7 (2.9 , 4.6)** | **1475 (1119 , 1857)** | **4 (3 , 4.9)** | **6.2 (5.2 , 7.3)** |
| **Madagascar** | **493 (379 , 625)** | **6.4 (5 , 8)** | **1294 (993 , 1664)** | **6.7 (5.2 , 8.4)** | **4.9 (0.5 , 8.6)** |
| **Malawi** | **314 (236 , 403)** | **5.2 (4 , 6.5)** | **665 (508 , 848)** | **5.5 (4.2 , 6.8)** | **6.1 (1.8 , 10.2)** |
| **Mozambique** | **531 (408 , 660)** | **6 (4.6 , 7.4)** | **1187 (913 , 1496)** | **6.5 (5 , 8)** | **7.8 (3.6 , 13)** |
| **Rwanda** | **160 (120 , 204)** | **3.6 (2.7 , 4.5)** | **360 (272 , 461)** | **3.8 (2.9 , 4.8)** | **5.8 (1.1 , 10.1)** |
| **Somalia** | **185 (138 , 235)** | **4 (3 , 5)** | **519 (387 , 662)** | **4.1 (3.1 , 5.2)** | **4 (-0.1 , 8.4)** |
| **South Sudan** | **152 (116 , 194)** | **4.1 (3.2 , 5.1)** | **275 (209 , 347)** | **4.4 (3.4 , 5.5)** | **7.7 (3.2 , 12.4)** |
| **United Republic of Tanzania** | **660 (500 , 825)** | **4.1 (3.1 , 5.1)** | **1673 (1276 , 2118)** | **4.3 (3.3 , 5.4)** | **5.5 (0.7 , 9.5)** |
| **Uganda** | **335 (248 , 424)** | **3.3 (2.5 , 4.1)** | **879 (653 , 1108)** | **3.6 (2.7 , 4.5)** | **8 (4 , 11.7)** |
| **Zambia** | **256 (196 , 325)** | **5.4 (4.1 , 6.6)** | **696 (529 , 885)** | **5.7 (4.4 , 7)** | **5.4 (0.7 , 10.1)** |
| **Central Sub-Saharan Africa** | **1371 (1047 , 1743)** | **3.8 (2.9 , 4.8)** | **3665 (2759 , 4617)** | **4.1 (3.1 , 5.1)** | **6.4 (3.4 , 9.6)** |
| **Angola** | **332 (252 , 426)** | **4.9 (3.8 , 6.1)** | **1044 (781 , 1308)** | **5.3 (4.1 , 6.6)** | **9.2 (4.4 , 13.8)** |
| **Central African Republic** | **75 (57 , 94)** | **4 (3 , 5)** | **156 (117 , 198)** | **4.1 (3.1 , 5.1)** | **2.6 (-1.7 , 7.5)** |
| **Congo** | **55 (42 , 70)** | **3.5 (2.7 , 4.4)** | **157 (120 , 199)** | **3.6 (2.8 , 4.5)** | **4.6 (0.7 , 8.4)** |
| **Democratic Republic of the Congo** | **874 (663 , 1107)** | **3.6 (2.7 , 4.5)** | **2217 (1663 , 2792)** | **3.7 (2.8 , 4.6)** | **4 (-0.1 , 8.4)** |
| **Equatorial Guinea** | **10 (7 , 12)** | **3.4 (2.6 , 4.3)** | **34 (26 , 44)** | **3.7 (2.9 , 4.6)** | **9 (4.9 , 13.7)** |
| **Gabon** | **24 (18 , 30)** | **3.4 (2.6 , 4.3)** | **56 (43 , 70)** | **3.7 (2.9 , 4.6)** | **8.7 (4.5 , 13.1)** |
