# Supplementary material for: Global burden of multiple sclerosis and its attributable risk factors, 1990–2019
Source: Front Neurol. 2024 Oct 25;15:1448377. doi: 10.3389/fneur.2024.1448377 (PMC11545682; doi:10.3389/fneur.2024.1448377)
Supplement: Supplementary file 19 [file Table_3.DOC]

| **Table S3: Deaths due to multiple sclerosis in 1990 and 2019 and the percentage change in the age-standardised rates (ASRs) per 100,000, by location**  **(Generated from data available from http://ghdx.healthdata.org/gbd-results-tool)** | | | | | |
| --- | --- | --- | --- | --- | --- |
|  | **1990** | | **2019** | | **Percentage change in ASRs per 100,000** |
|  | **No (95% UI)** | **ASRs per 100,000 (95% UI)** | **No (95% UI)** | **ASRs per 100,000 (95% UI)** |
| **Global** | **13356 (11904 , 17571)** | **0.3 (0.3 , 0.4)** | **22439 (20226 , 27792)** | **0.3 (0.2 , 0.3)** | **-14 (-29.1 , -5.9)** |
| **High-income North America** | **2085 (1886 , 2912)** | **0.6 (0.6 , 0.9)** | **4632 (3116 , 5134)** | **0.8 (0.6 , 0.9)** | **25.1 (-16.2 , 44.7)** |
| **Canada** | **238 (178 , 275)** | **0.8 (0.6 , 0.9)** | **506 (278 , 601)** | **0.8 (0.5 , 1)** | **9 (-29.7 , 25.5)** |
| **Greenland** | **0 (0 , 0)** | **0.3 (0.2 , 0.4)** | **0 (0 , 0)** | **0.3 (0.2 , 0.4)** | **0.5 (-36.6 , 45.5)** |
| **United States of America** | **1846 (1661 , 2631)** | **0.6 (0.6 , 0.9)** | **4126 (2821 , 4564)** | **0.8 (0.6 , 0.9)** | **27 (-14.6 , 47.9)** |
| **Australasia** | **109 (94 , 160)** | **0.5 (0.4 , 0.7)** | **215 (156 , 276)** | **0.5 (0.4 , 0.6)** | **2.6 (-36.4 , 19.5)** |
| **Australia** | **88 (74 , 134)** | **0.5 (0.4 , 0.7)** | **178 (132 , 233)** | **0.5 (0.4 , 0.6)** | **4.8 (-35.6 , 25)** |
| **New Zealand** | **21 (16 , 27)** | **0.6 (0.4 , 0.7)** | **36 (24 , 43)** | **0.5 (0.3 , 0.6)** | **-6.2 (-40.7 , 11.9)** |
| **High-income Asia Pacific** | **237 (210 , 351)** | **0.1 (0.1 , 0.2)** | **321 (266 , 498)** | **0.1 (0.1 , 0.1)** | **-18.3 (-37.6 , 2.5)** |
| **Brunei Darussalam** | **0 (0 , 0)** | **0.1 (0.1 , 0.2)** | **0 (0 , 1)** | **0.1 (0.1 , 0.2)** | **-4.6 (-25.3 , 21.9)** |
| **Japan** | **191 (168 , 291)** | **0.1 (0.1 , 0.2)** | **240 (191 , 393)** | **0.1 (0.1 , 0.2)** | **-14.9 (-31.6 , 10.2)** |
| **Singapore** | **3 (2 , 3)** | **0.1 (0.1 , 0.1)** | **6 (4 , 7)** | **0.1 (0 , 0.1)** | **-29 (-54.1 , 13)** |
| **Republic of Korea** | **44 (37 , 65)** | **0.1 (0.1 , 0.2)** | **75 (60 , 110)** | **0.1 (0.1 , 0.1)** | **-25 (-55.4 , -0.7)** |
| **Western Europe** | **3722 (3359 , 5327)** | **0.7 (0.6 , 1)** | **5235 (3829 , 6538)** | **0.7 (0.5 , 0.9)** | **-2.8 (-36.2 , 9)** |
| **Andorra** | **0 (0 , 1)** | **0.7 (0.4 , 1.2)** | **1 (0 , 2)** | **0.8 (0.4 , 1.3)** | **10.3 (-29.2 , 55.8)** |
| **Austria** | **62 (54 , 90)** | **0.6 (0.5 , 0.9)** | **110 (65 , 129)** | **0.7 (0.4 , 0.8)** | **21.8 (-39.5 , 53.3)** |
| **Belgium** | **94 (66 , 105)** | **0.7 (0.5 , 0.7)** | **122 (75 , 143)** | **0.6 (0.4 , 0.7)** | **-5.9 (-26.2 , 15.7)** |
| **Cyprus** | **3 (2 , 4)** | **0.3 (0.2 , 0.5)** | **7 (4 , 8)** | **0.4 (0.2 , 0.4)** | **16.1 (-43.3 , 66.9)** |
| **Denmark** | **96 (54 , 114)** | **1.3 (0.8 , 1.6)** | **110 (64 , 134)** | **1.1 (0.7 , 1.3)** | **-17.3 (-39.4 , 11.2)** |
| **Finland** | **39 (32 , 52)** | **0.6 (0.5 , 0.8)** | **63 (36 , 76)** | **0.7 (0.4 , 0.8)** | **9.2 (-40.8 , 36.6)** |
| **France** | **380 (315 , 485)** | **0.5 (0.4 , 0.7)** | **587 (354 , 694)** | **0.5 (0.3 , 0.6)** | **3.4 (-32.2 , 18.3)** |
| **Germany** | **1030 (895 , 1561)** | **0.9 (0.8 , 1.4)** | **1278 (1009 , 1727)** | **0.8 (0.7 , 1.1)** | **-9.4 (-40 , 4.1)** |
| **Greece** | **44 (38 , 66)** | **0.3 (0.3 , 0.5)** | **86 (55 , 99)** | **0.5 (0.3 , 0.6)** | **57 (-11.4 , 101.9)** |
| **Iceland** | **2 (2 , 4)** | **0.9 (0.7 , 1.3)** | **3 (3 , 6)** | **0.7 (0.5 , 1.1)** | **-24.8 (-41.4 , 2.9)** |
| **Ireland** | **34 (22 , 38)** | **0.9 (0.6 , 1)** | **55 (29 , 67)** | **0.8 (0.4 , 1)** | **-9.8 (-43.2 , 15.4)** |
| **Israel** | **9 (8 , 11)** | **0.2 (0.2 , 0.2)** | **25 (14 , 30)** | **0.2 (0.1 , 0.3)** | **19.1 (-35.8 , 43.3)** |
| **Italy** | **334 (287 , 530)** | **0.4 (0.4 , 0.7)** | **471 (356 , 660)** | **0.4 (0.3 , 0.6)** | **-0.1 (-38.7 , 15)** |
| **Luxembourg** | **5 (4 , 8)** | **1 (0.8 , 1.5)** | **7 (5 , 12)** | **0.8 (0.6 , 1.3)** | **-16.7 (-48.8 , 6.4)** |
| **Malta** | **1 (1 , 2)** | **0.3 (0.3 , 0.5)** | **2 (2 , 3)** | **0.3 (0.2 , 0.4)** | **-6.9 (-32.6 , 13.8)** |
| **Monaco** | **0 (0 , 0)** | **0.3 (0.2 , 0.5)** | **0 (0 , 0)** | **0.4 (0.3 , 0.5)** | **24.4 (-31.2 , 101.7)** |
| **Netherlands** | **172 (132 , 205)** | **0.9 (0.7 , 1.1)** | **235 (172 , 280)** | **0.8 (0.6 , 1)** | **-12.1 (-29.1 , 6.6)** |
| **Norway** | **59 (38 , 66)** | **1 (0.7 , 1.2)** | **85 (39 , 105)** | **1 (0.5 , 1.2)** | **-3.5 (-41.2 , 9.5)** |
| **Portugal** | **45 (38 , 71)** | **0.4 (0.3 , 0.5)** | **55 (43 , 97)** | **0.3 (0.2 , 0.5)** | **-17.6 (-33.1 , 19.4)** |
| **San Marino** | **0 (0 , 0)** | **0.1 (0.1 , 0.1)** | **0 (0 , 0)** | **0.1 (0.1 , 0.2)** | **-0.2 (-41.1 , 63.5)** |
| **Spain** | **172 (132 , 303)** | **0.4 (0.3 , 0.6)** | **270 (218 , 450)** | **0.3 (0.3 , 0.6)** | **-1.8 (-35.6 , 16.6)** |
| **Sweden** | **83 (66 , 102)** | **0.7 (0.5 , 0.8)** | **126 (71 , 151)** | **0.7 (0.4 , 0.8)** | **6.9 (-35.2 , 25)** |
| **Switzerland** | **99 (72 , 123)** | **1 (0.8 , 1.3)** | **132 (89 , 156)** | **0.9 (0.6 , 1.1)** | **-16.7 (-41.5 , 6.3)** |
| **United Kingdom** | **954 (847 , 1424)** | **1.2 (1.1 , 1.8)** | **1399 (1049 , 1717)** | **1.3 (1 , 1.6)** | **6.9 (-30 , 20.1)** |
| **Southern Latin America** | **131 (117 , 186)** | **0.3 (0.3 , 0.4)** | **185 (150 , 315)** | **0.2 (0.2 , 0.4)** | **-16.5 (-28.1 , 16.8)** |
| **Argentina** | **103 (90 , 153)** | **0.3 (0.3 , 0.5)** | **140 (106 , 257)** | **0.3 (0.2 , 0.5)** | **-14.9 (-29.9 , 20.8)** |
| **Chile** | **16 (14 , 22)** | **0.1 (0.1 , 0.2)** | **30 (25 , 45)** | **0.1 (0.1 , 0.2)** | **-7.7 (-33.4 , 25.9)** |
| **Uruguay** | **12 (7 , 14)** | **0.3 (0.2 , 0.4)** | **15 (9 , 18)** | **0.3 (0.2 , 0.4)** | **-4 (-15.3 , 15.3)** |
| **Eastern Europe** | **1522 (1300 , 2260)** | **0.6 (0.5 , 0.9)** | **1421 (942 , 2848)** | **0.5 (0.3 , 1)** | **-14.7 (-37.5 , 29.6)** |
| **Belarus** | **44 (37 , 58)** | **0.4 (0.3 , 0.5)** | **46 (29 , 81)** | **0.3 (0.2 , 0.6)** | **-5.4 (-39 , 47.1)** |
| **Estonia** | **13 (7 , 16)** | **0.7 (0.4 , 0.9)** | **9 (5 , 13)** | **0.5 (0.3 , 0.7)** | **-30.1 (-51.7 , 26.3)** |
| **Latvia** | **26 (15 , 30)** | **0.8 (0.5 , 1)** | **18 (11 , 24)** | **0.7 (0.4 , 0.9)** | **-16.5 (-37.5 , 31.1)** |
| **Lithuania** | **32 (18 , 38)** | **0.8 (0.4 , 0.9)** | **26 (16 , 34)** | **0.6 (0.4 , 0.8)** | **-15.7 (-36.7 , 31.8)** |
| **Republic of Moldova** | **9 (7 , 15)** | **0.2 (0.2 , 0.3)** | **11 (8 , 17)** | **0.2 (0.2 , 0.3)** | **9.5 (-22.8 , 37.7)** |
| **Russian Federation** | **936 (803 , 1331)** | **0.6 (0.5 , 0.8)** | **843 (559 , 1659)** | **0.4 (0.3 , 0.9)** | **-22.4 (-44.7 , 21.2)** |
| **Ukraine** | **463 (366 , 807)** | **0.7 (0.6 , 1.3)** | **468 (255 , 1085)** | **0.8 (0.4 , 1.8)** | **5.7 (-31.1 , 68.5)** |
| **Central Europe** | **1250 (1052 , 1548)** | **0.9 (0.7 , 1.1)** | **1148 (872 , 1845)** | **0.6 (0.5 , 1.1)** | **-26.7 (-44.1 , 18.5)** |
| **Albania** | **32 (27 , 41)** | **1.6 (1.2 , 1.9)** | **33 (20 , 64)** | **0.9 (0.5 , 1.7)** | **-46.6 (-70.6 , 15.7)** |
| **Bosnia and Herzegovina** | **23 (20 , 28)** | **0.5 (0.4 , 0.6)** | **21 (15 , 36)** | **0.4 (0.3 , 0.7)** | **-14.3 (-39.3 , 30.7)** |
| **Bulgaria** | **73 (57 , 124)** | **0.7 (0.6 , 1.1)** | **78 (54 , 135)** | **0.7 (0.5 , 1.2)** | **-0.1 (-23.9 , 28.6)** |
| **Croatia** | **34 (26 , 41)** | **0.5 (0.4 , 0.7)** | **39 (22 , 54)** | **0.6 (0.3 , 0.8)** | **4 (-41.7 , 42.8)** |
| **Czechia** | **130 (76 , 152)** | **1 (0.6 , 1.2)** | **100 (71 , 138)** | **0.6 (0.4 , 0.8)** | **-43.1 (-59.6 , 13)** |
| **Hungary** | **105 (73 , 119)** | **0.8 (0.5 , 0.9)** | **89 (67 , 124)** | **0.6 (0.4 , 0.8)** | **-26.7 (-45.4 , 19)** |
| **Montenegro** | **5 (4 , 8)** | **0.8 (0.6 , 1.2)** | **7 (5 , 11)** | **0.8 (0.5 , 1.2)** | **-2.3 (-25.1 , 28.7)** |
| **North Macedonia** | **10 (8 , 13)** | **0.5 (0.4 , 0.7)** | **15 (11 , 21)** | **0.5 (0.4 , 0.7)** | **6.6 (-21.1 , 40)** |
| **Poland** | **558 (498 , 797)** | **1.3 (1.2 , 1.9)** | **514 (333 , 1056)** | **0.9 (0.6 , 1.8)** | **-33.2 (-54.2 , 23.5)** |
| **Romania** | **148 (80 , 175)** | **0.6 (0.3 , 0.7)** | **87 (64 , 138)** | **0.3 (0.2 , 0.5)** | **-46.8 (-65 , 22.3)** |
| **Serbia** | **89 (71 , 124)** | **0.8 (0.6 , 1.1)** | **101 (76 , 134)** | **0.8 (0.6 , 1.1)** | **0.7 (-27.9 , 36.4)** |
| **Slovakia** | **25 (20 , 30)** | **0.4 (0.4 , 0.5)** | **38 (23 , 52)** | **0.5 (0.3 , 0.6)** | **7.8 (-38.9 , 56)** |
| **Slovenia** | **20 (12 , 28)** | **0.8 (0.5 , 1.2)** | **24 (12 , 33)** | **0.7 (0.3 , 0.9)** | **-21.6 (-52.7 , 25.8)** |
| **Central Asia** | **101 (81 , 122)** | **0.2 (0.2 , 0.2)** | **146 (117 , 223)** | **0.2 (0.2 , 0.3)** | **-5.9 (-21.9 , 31.8)** |
| **Armenia** | **5 (4 , 6)** | **0.2 (0.1 , 0.2)** | **9 (6 , 11)** | **0.2 (0.2 , 0.3)** | **36.7 (-6.2 , 74.9)** |
| **Azerbaijan** | **6 (5 , 7)** | **0.1 (0.1 , 0.1)** | **12 (9 , 16)** | **0.1 (0.1 , 0.2)** | **7.1 (-16.8 , 38.8)** |
| **Georgia** | **6 (5 , 8)** | **0.1 (0.1 , 0.1)** | **10 (5 , 13)** | **0.2 (0.1 , 0.2)** | **67.9 (-15 , 130.4)** |
| **Kazakhstan** | **44 (37 , 63)** | **0.4 (0.3 , 0.5)** | **56 (37 , 102)** | **0.4 (0.3 , 0.6)** | **-3.4 (-26.5 , 36.3)** |
| **Kyrgyzstan** | **5 (3 , 5)** | **0.1 (0.1 , 0.2)** | **6 (5 , 8)** | **0.1 (0.1 , 0.2)** | **-23 (-42 , 29.7)** |
| **Mongolia** | **1 (0 , 2)** | **0.1 (0 , 0.2)** | **3 (2 , 4)** | **0.1 (0.1 , 0.2)** | **68.2 (-17.3 , 300.7)** |
| **Tajikistan** | **3 (3 , 4)** | **0.1 (0.1 , 0.1)** | **8 (6 , 10)** | **0.1 (0.1 , 0.2)** | **15 (-9.4 , 51.3)** |
| **Turkmenistan** | **5 (4 , 6)** | **0.3 (0.2 , 0.3)** | **12 (9 , 16)** | **0.3 (0.2 , 0.4)** | **5.2 (-19.2 , 44.7)** |
| **Uzbekistan** | **26 (13 , 32)** | **0.2 (0.1 , 0.3)** | **32 (23 , 60)** | **0.1 (0.1 , 0.3)** | **-38.4 (-63.1 , 101.4)** |
| **Central Latin America** | **154 (133 , 240)** | **0.2 (0.1 , 0.2)** | **533 (414 , 683)** | **0.2 (0.2 , 0.3)** | **40.5 (-7.2 , 80.9)** |
| **Colombia** | **28 (24 , 41)** | **0.1 (0.1 , 0.2)** | **80 (53 , 113)** | **0.2 (0.1 , 0.2)** | **16.1 (-32.6 , 60.8)** |
| **Costa Rica** | **3 (3 , 4)** | **0.2 (0.1 , 0.2)** | **10 (7 , 13)** | **0.2 (0.1 , 0.3)** | **15.6 (-15.3 , 52.7)** |
| **El Salvador** | **3 (3 , 4)** | **0.1 (0.1 , 0.1)** | **7 (5 , 10)** | **0.1 (0.1 , 0.2)** | **17.5 (-19.4 , 57.5)** |
| **Guatemala** | **5 (4 , 7)** | **0.1 (0.1 , 0.2)** | **20 (13 , 27)** | **0.2 (0.1 , 0.2)** | **32.8 (-21.4 , 86.2)** |
| **Honduras** | **3 (3 , 4)** | **0.1 (0.1 , 0.2)** | **13 (8 , 21)** | **0.2 (0.1 , 0.3)** | **37.4 (-5 , 103.2)** |
| **Mexico** | **86 (69 , 153)** | **0.2 (0.1 , 0.3)** | **326 (254 , 427)** | **0.3 (0.2 , 0.3)** | **56.5 (-5.2 , 118.3)** |
| **Nicaragua** | **2 (2 , 3)** | **0.1 (0.1 , 0.2)** | **8 (6 , 10)** | **0.2 (0.1 , 0.2)** | **27.5 (-3.3 , 71.8)** |
| **Panama** | **2 (2 , 3)** | **0.1 (0.1 , 0.2)** | **6 (4 , 8)** | **0.1 (0.1 , 0.2)** | **0.9 (-26.6 , 38.3)** |
| **Venezuela (Bolivarian Republic of)** | **20 (16 , 25)** | **0.2 (0.1 , 0.2)** | **64 (44 , 87)** | **0.2 (0.1 , 0.3)** | **24.1 (-11.1 , 67.7)** |
| **Andean Latin America** | **31 (26 , 39)** | **0.1 (0.1 , 0.2)** | **79 (59 , 101)** | **0.1 (0.1 , 0.2)** | **2.9 (-28 , 37.6)** |
| **Bolivia (Plurinational State of)** | **7 (5 , 10)** | **0.2 (0.1 , 0.3)** | **20 (14 , 27)** | **0.2 (0.2 , 0.3)** | **11 (-23.9 , 62.3)** |
| **Ecuador** | **8 (7 , 9)** | **0.1 (0.1 , 0.2)** | **22 (16 , 29)** | **0.1 (0.1 , 0.2)** | **10.8 (-19.4 , 46.7)** |
| **Peru** | **16 (13 , 20)** | **0.1 (0.1 , 0.1)** | **37 (25 , 53)** | **0.1 (0.1 , 0.2)** | **-5.4 (-41.3 , 45)** |
| **Caribbean** | **59 (49 , 72)** | **0.2 (0.2 , 0.3)** | **123 (92 , 156)** | **0.2 (0.2 , 0.3)** | **14.2 (-11.5 , 37.5)** |
| **Antigua and Barbuda** | **0 (0 , 0)** | **0.3 (0.2 , 0.4)** | **0 (0 , 0)** | **0.4 (0.2 , 0.5)** | **22.5 (-18.8 , 60.9)** |
| **Barbados** | **1 (1 , 1)** | **0.3 (0.3 , 0.5)** | **2 (1 , 2)** | **0.4 (0.3 , 0.6)** | **28 (-13.5 , 72.4)** |
| **Belize** | **0 (0 , 0)** | **0.1 (0.1 , 0.2)** | **0 (0 , 1)** | **0.1 (0.1 , 0.2)** | **29.2 (2.2 , 80.4)** |
| **Bermuda** | **0 (0 , 0)** | **0.2 (0.2 , 0.3)** | **0 (0 , 0)** | **0.2 (0.1 , 0.3)** | **-18.7 (-48.2 , 13.1)** |
| **Bahamas** | **1 (0 , 1)** | **0.3 (0.2 , 0.4)** | **1 (1 , 2)** | **0.3 (0.2 , 0.4)** | **15.9 (-17.9 , 59.5)** |
| **Cuba** | **26 (21 , 33)** | **0.2 (0.2 , 0.3)** | **49 (32 , 63)** | **0.3 (0.2 , 0.4)** | **17.8 (-24.4 , 51.2)** |
| **Dominica** | **0 (0 , 0)** | **0.1 (0.1 , 0.2)** | **0 (0 , 0)** | **0.1 (0.1 , 0.2)** | **7.3 (-18.8 , 40.4)** |
| **Dominican Republic** | **5 (4 , 6)** | **0.1 (0.1 , 0.1)** | **15 (10 , 23)** | **0.2 (0.1 , 0.2)** | **39.4 (-7.2 , 111.6)** |
| **Grenada** | **0 (0 , 0)** | **0.3 (0.3 , 0.5)** | **0 (0 , 1)** | **0.3 (0.3 , 0.5)** | **-3.7 (-20.6 , 31.3)** |
| **Guyana** | **1 (0 , 1)** | **0.1 (0.1 , 0.2)** | **1 (1 , 2)** | **0.1 (0.1 , 0.2)** | **16.6 (-15.8 , 58.3)** |
| **Haiti** | **11 (7 , 20)** | **0.3 (0.2 , 0.5)** | **25 (15 , 44)** | **0.3 (0.2 , 0.5)** | **3.3 (-30.9 , 49.6)** |
| **Jamaica** | **2 (1 , 2)** | **0.1 (0.1 , 0.1)** | **4 (3 , 5)** | **0.1 (0.1 , 0.2)** | **18 (-13 , 59.2)** |
| **Puerto Rico** | **9 (6 , 11)** | **0.2 (0.2 , 0.3)** | **15 (9 , 20)** | **0.3 (0.2 , 0.4)** | **15.9 (-25.9 , 59.1)** |
| **Saint Kitts and Nevis** | **0 (0 , 0)** | **0.5 (0.4 , 0.7)** | **0 (0 , 1)** | **0.5 (0.3 , 0.7)** | **-4.8 (-34.2 , 39.3)** |
| **Saint Lucia** | **0 (0 , 0)** | **0.2 (0.1 , 0.3)** | **0 (0 , 1)** | **0.2 (0.1 , 0.3)** | **9.2 (-15.9 , 37)** |
| **Saint Vincent and the Grenadines** | **0 (0 , 0)** | **0.2 (0.1 , 0.2)** | **0 (0 , 0)** | **0.2 (0.1 , 0.2)** | **12.9 (-18 , 45)** |
| **Suriname** | **0 (0 , 1)** | **0.1 (0.1 , 0.2)** | **1 (1 , 1)** | **0.2 (0.1 , 0.2)** | **17.5 (-8.7 , 49.1)** |
| **Trinidad and Tobago** | **1 (1 , 1)** | **0.1 (0.1 , 0.2)** | **3 (2 , 4)** | **0.2 (0.1 , 0.2)** | **26.1 (-24.8 , 84.3)** |
| **United States Virgin Islands** | **0 (0 , 0)** | **0.2 (0.2 , 0.3)** | **0 (0 , 0)** | **0.2 (0.2 , 0.3)** | **-0.4 (-27.2 , 42)** |
| **Tropical Latin America** | **158 (138 , 241)** | **0.2 (0.1 , 0.2)** | **421 (362 , 599)** | **0.2 (0.1 , 0.2)** | **5.9 (-20.9 , 25)** |
| **Brazil** | **156 (135 , 239)** | **0.2 (0.1 , 0.2)** | **413 (354 , 592)** | **0.2 (0.1 , 0.2)** | **5.2 (-21.7 , 25.2)** |
| **Paraguay** | **2 (2 , 3)** | **0.1 (0.1 , 0.1)** | **8 (4 , 11)** | **0.1 (0.1 , 0.2)** | **35.1 (-7.6 , 87.1)** |
| **East Asia** | **1274 (923 , 1543)** | **0.1 (0.1 , 0.2)** | **1888 (1527 , 2506)** | **0.1 (0.1 , 0.1)** | **-27.5 (-46.1 , 10.7)** |
| **China** | **1224 (884 , 1487)** | **0.1 (0.1 , 0.2)** | **1804 (1437 , 2414)** | **0.1 (0.1 , 0.1)** | **-28.1 (-47.2 , 11.2)** |
| **Democratic People's Republic of Korea** | **31 (19 , 46)** | **0.2 (0.1 , 0.2)** | **47 (33 , 69)** | **0.1 (0.1 , 0.2)** | **-13 (-39.4 , 34.5)** |
| **Taiwan (Province of China)** | **20 (16 , 24)** | **0.1 (0.1 , 0.1)** | **37 (28 , 55)** | **0.1 (0.1 , 0.1)** | **-7.5 (-30.5 , 34.6)** |
| **Southeast Asia** | **390 (325 , 537)** | **0.1 (0.1 , 0.2)** | **771 (593 , 1106)** | **0.1 (0.1 , 0.2)** | **-6 (-26.5 , 30.4)** |
| **Cambodia** | **8 (5 , 13)** | **0.1 (0.1 , 0.2)** | **18 (13 , 26)** | **0.1 (0.1 , 0.2)** | **-2.6 (-37.3 , 41.9)** |
| **Indonesia** | **120 (94 , 176)** | **0.1 (0.1 , 0.1)** | **261 (171 , 407)** | **0.1 (0.1 , 0.2)** | **9.4 (-20.7 , 49.8)** |
| **Lao People's Democratic Republic** | **4 (2 , 7)** | **0.2 (0.1 , 0.3)** | **7 (5 , 10)** | **0.1 (0.1 , 0.2)** | **-19 (-49.2 , 28)** |
| **Malaysia** | **11 (8 , 13)** | **0.1 (0.1 , 0.1)** | **28 (21 , 38)** | **0.1 (0.1 , 0.1)** | **2.9 (-24.8 , 47.2)** |
| **Maldives** | **0 (0 , 0)** | **0.1 (0.1 , 0.1)** | **0 (0 , 1)** | **0.1 (0 , 0.1)** | **-26 (-62.9 , 16.3)** |
| **Mauritius** | **1 (1 , 1)** | **0.1 (0.1 , 0.1)** | **2 (1 , 3)** | **0.1 (0.1 , 0.2)** | **-4.6 (-26.4 , 27.6)** |
| **Myanmar** | **45 (28 , 84)** | **0.2 (0.1 , 0.3)** | **70 (51 , 106)** | **0.1 (0.1 , 0.2)** | **-15.4 (-47.8 , 31.3)** |
| **Philippines** | **52 (42 , 78)** | **0.1 (0.1 , 0.2)** | **106 (75 , 178)** | **0.1 (0.1 , 0.2)** | **-13 (-32.9 , 25.7)** |
| **Sri Lanka** | **34 (24 , 57)** | **0.2 (0.2 , 0.3)** | **32 (17 , 70)** | **0.1 (0.1 , 0.3)** | **-41.6 (-73.3 , 14.3)** |
| **Seychelles** | **0 (0 , 0)** | **0.2 (0.1 , 0.2)** | **0 (0 , 0)** | **0.2 (0.1 , 0.3)** | **13.8 (-19.1 , 56.5)** |
| **Thailand** | **58 (44 , 81)** | **0.1 (0.1 , 0.2)** | **99 (70 , 143)** | **0.1 (0.1 , 0.1)** | **-22.1 (-50.6 , 18.3)** |
| **Timor-Leste** | **0 (0 , 1)** | **0.1 (0.1 , 0.1)** | **1 (1 , 1)** | **0.1 (0.1 , 0.1)** | **13.3 (-26.9 , 90.9)** |
| **Viet Nam** | **58 (38 , 86)** | **0.1 (0.1 , 0.2)** | **144 (103 , 210)** | **0.1 (0.1 , 0.2)** | **4.9 (-28.1 , 63.6)** |
| **Oceania** | **4 (2 , 5)** | **0.1 (0.1 , 0.1)** | **8 (6 , 12)** | **0.1 (0.1 , 0.1)** | **-10.2 (-28.3 , 16.8)** |
| **American Samoa** | **0 (0 , 0)** | **0.1 (0.1 , 0.1)** | **0 (0 , 0)** | **0.1 (0.1 , 0.1)** | **-9 (-30.4 , 24.6)** |
| **Cook Islands** | **0 (0 , 0)** | **0.1 (0.1 , 0.2)** | **0 (0 , 0)** | **0.1 (0.1 , 0.1)** | **-29.2 (-47.6 , -4.9)** |
| **Micronesia (Federated States of)** | **0 (0 , 0)** | **0.2 (0.1 , 0.2)** | **0 (0 , 0)** | **0.1 (0.1 , 0.2)** | **-14.3 (-45.9 , 31.6)** |
| **Fiji** | **1 (0 , 1)** | **0.1 (0.1 , 0.1)** | **1 (1 , 1)** | **0.1 (0.1 , 0.1)** | **-9.4 (-35.7 , 31.3)** |
| **Guam** | **0 (0 , 0)** | **0.1 (0.1 , 0.1)** | **0 (0 , 0)** | **0.1 (0.1 , 0.1)** | **-20.1 (-38.9 , 13.6)** |
| **Kiribati** | **0 (0 , 0)** | **0.2 (0.2 , 0.4)** | **0 (0 , 0)** | **0.2 (0.1 , 0.3)** | **-14.3 (-34.4 , 9.2)** |
| **Marshall Islands** | **0 (0 , 0)** | **0.2 (0.1 , 0.2)** | **0 (0 , 0)** | **0.1 (0.1 , 0.2)** | **-10.1 (-35.4 , 43.9)** |
| **Nauru** | **0 (0 , 0)** | **0.1 (0.1 , 0.2)** | **0 (0 , 0)** | **0.1 (0.1 , 0.2)** | **-14.7 (-33.6 , 18.4)** |
| **Niue** | **0 (0 , 0)** | **0.2 (0.1 , 0.2)** | **0 (0 , 0)** | **0.1 (0.1 , 0.2)** | **-22.3 (-46.2 , 17.7)** |
| **Northern Mariana Islands** | **0 (0 , 0)** | **0.1 (0.1 , 0.2)** | **0 (0 , 0)** | **0.1 (0.1 , 0.1)** | **-12.9 (-33 , 9.9)** |
| **Palau** | **0 (0 , 0)** | **0.1 (0.1 , 0.2)** | **0 (0 , 0)** | **0.1 (0.1 , 0.1)** | **-6.4 (-34.5 , 29.4)** |
| **Papua New Guinea** | **2 (1 , 3)** | **0.1 (0 , 0.1)** | **5 (3 , 8)** | **0.1 (0 , 0.1)** | **-5.1 (-29.9 , 33)** |
| **Samoa** | **0 (0 , 0)** | **0.1 (0.1 , 0.2)** | **0 (0 , 0)** | **0.1 (0.1 , 0.2)** | **-19.6 (-41.9 , 12.7)** |
| **Solomon Islands** | **0 (0 , 0)** | **0.2 (0.1 , 0.3)** | **1 (0 , 1)** | **0.1 (0.1 , 0.2)** | **-6.9 (-32.3 , 35.7)** |
| **Tokelau** | **0 (0 , 0)** | **0.1 (0.1 , 0.2)** | **0 (0 , 0)** | **0.1 (0.1 , 0.1)** | **-22.3 (-46.9 , 23.6)** |
| **Tonga** | **0 (0 , 0)** | **0.1 (0.1 , 0.1)** | **0 (0 , 0)** | **0.1 (0.1 , 0.1)** | **-6.4 (-30.7 , 34)** |
| **Tuvalu** | **0 (0 , 0)** | **0.1 (0.1 , 0.2)** | **0 (0 , 0)** | **0.1 (0.1 , 0.2)** | **-22.3 (-45.3 , 17.3)** |
| **Vanuatu** | **0 (0 , 0)** | **0.1 (0.1 , 0.2)** | **0 (0 , 0)** | **0.1 (0.1 , 0.2)** | **0 (-28.9 , 51.4)** |
| **North Africa and Middle East** | **581 (435 , 778)** | **0.3 (0.2 , 0.4)** | **1436 (1176 , 1814)** | **0.3 (0.2 , 0.3)** | **5.1 (-25 , 47.6)** |
| **Afghanistan** | **31 (14 , 74)** | **0.4 (0.2 , 0.9)** | **90 (36 , 205)** | **0.4 (0.2 , 0.9)** | **8.8 (-28.9 , 66.6)** |
| **Algeria** | **38 (21 , 59)** | **0.3 (0.2 , 0.4)** | **101 (52 , 142)** | **0.3 (0.1 , 0.4)** | **1.6 (-38.3 , 50.7)** |
| **Bahrain** | **0 (0 , 0)** | **0.1 (0.1 , 0.2)** | **2 (1 , 3)** | **0.1 (0.1 , 0.2)** | **-6.6 (-44.1 , 28.6)** |
| **Egypt** | **97 (65 , 169)** | **0.2 (0.1 , 0.3)** | **191 (96 , 426)** | **0.2 (0.1 , 0.4)** | **5.9 (-48 , 122.5)** |
| **Iran (Islamic Republic of)** | **144 (96 , 239)** | **0.4 (0.3 , 0.8)** | **396 (328 , 587)** | **0.5 (0.4 , 0.7)** | **4.8 (-28.2 , 55.7)** |
| **Iraq** | **12 (9 , 17)** | **0.1 (0.1 , 0.2)** | **40 (28 , 57)** | **0.1 (0.1 , 0.2)** | **4.8 (-31.5 , 69.7)** |
| **Jordan** | **4 (3 , 6)** | **0.2 (0.2 , 0.3)** | **17 (11 , 23)** | **0.2 (0.1 , 0.2)** | **-14.5 (-43.1 , 18)** |
| **Kuwait** | **1 (1 , 2)** | **0.1 (0.1 , 0.2)** | **5 (4 , 7)** | **0.1 (0.1 , 0.2)** | **0.8 (-23 , 34.7)** |
| **Lebanon** | **6 (4 , 9)** | **0.3 (0.2 , 0.3)** | **15 (8 , 21)** | **0.3 (0.1 , 0.4)** | **7.5 (-29.5 , 66.7)** |
| **Libya** | **5 (3 , 7)** | **0.2 (0.1 , 0.3)** | **21 (11 , 35)** | **0.3 (0.2 , 0.5)** | **38.4 (-17.3 , 139.5)** |
| **Morocco** | **36 (19 , 54)** | **0.2 (0.1 , 0.3)** | **99 (51 , 148)** | **0.3 (0.1 , 0.4)** | **31.5 (-16.5 , 103.4)** |
| **Palestine** | **3 (2 , 4)** | **0.2 (0.1 , 0.4)** | **8 (6 , 12)** | **0.2 (0.2 , 0.4)** | **1.6 (-37.7 , 77.2)** |
| **Oman** | **2 (1 , 3)** | **0.2 (0.1 , 0.3)** | **8 (4 , 12)** | **0.3 (0.1 , 0.4)** | **16.2 (-42.4 , 115.1)** |
| **Qatar** | **0 (0 , 0)** | **0.1 (0.1 , 0.1)** | **2 (1 , 3)** | **0.1 (0.1 , 0.1)** | **-14.3 (-44.8 , 29.6)** |
| **Saudi Arabia** | **9 (4 , 13)** | **0.1 (0.1 , 0.2)** | **40 (27 , 60)** | **0.1 (0.1 , 0.2)** | **17.7 (-33.3 , 198.9)** |
| **Sudan** | **21 (10 , 34)** | **0.2 (0.1 , 0.3)** | **56 (25 , 89)** | **0.2 (0.1 , 0.3)** | **19.7 (-31.4 , 105.2)** |
| **Syrian Arab Republic** | **8 (5 , 12)** | **0.1 (0.1 , 0.2)** | **18 (12 , 25)** | **0.1 (0.1 , 0.2)** | **4.4 (-34.3 , 71.8)** |
| **Tunisia** | **12 (7 , 17)** | **0.2 (0.1 , 0.3)** | **35 (18 , 52)** | **0.3 (0.1 , 0.4)** | **24.3 (-22 , 102.2)** |
| **Turkey** | **134 (93 , 210)** | **0.3 (0.2 , 0.4)** | **206 (151 , 292)** | **0.2 (0.2 , 0.3)** | **-23.2 (-53.2 , 26)** |
| **United Arab Emirates** | **4 (2 , 5)** | **0.3 (0.2 , 0.5)** | **44 (20 , 80)** | **0.4 (0.2 , 0.7)** | **24.9 (-27.7 , 81.3)** |
| **Yemen** | **11 (5 , 20)** | **0.2 (0.1 , 0.3)** | **41 (20 , 67)** | **0.2 (0.1 , 0.3)** | **28.9 (-26.4 , 121.5)** |
| **South Asia** | **1190 (766 , 1754)** | **0.2 (0.1 , 0.3)** | **2915 (2387 , 3672)** | **0.2 (0.2 , 0.2)** | **8.2 (-18.3 , 64.4)** |
| **Bangladesh** | **99 (54 , 153)** | **0.2 (0.1 , 0.3)** | **219 (136 , 303)** | **0.2 (0.1 , 0.2)** | **-10.9 (-46.8 , 40.8)** |
| **Bhutan** | **1 (0 , 1)** | **0.2 (0.1 , 0.3)** | **1 (1 , 2)** | **0.2 (0.1 , 0.3)** | **9.5 (-36.6 , 80.1)** |
| **India** | **947 (622 , 1396)** | **0.2 (0.1 , 0.2)** | **2312 (1863 , 2934)** | **0.2 (0.1 , 0.2)** | **8.2 (-19.9 , 66.8)** |
| **Nepal** | **20 (10 , 36)** | **0.2 (0.1 , 0.3)** | **47 (28 , 66)** | **0.2 (0.1 , 0.3)** | **13.3 (-29.2 , 76.8)** |
| **Pakistan** | **124 (74 , 198)** | **0.2 (0.1 , 0.3)** | **336 (241 , 513)** | **0.2 (0.2 , 0.4)** | **24.6 (-8.5 , 107.4)** |
| **Southern Sub-Saharan Africa** | **42 (35 , 48)** | **0.1 (0.1 , 0.1)** | **87 (71 , 104)** | **0.1 (0.1 , 0.2)** | **4.2 (-9.7 , 21.6)** |
| **Botswana** | **1 (1 , 1)** | **0.1 (0.1 , 0.2)** | **3 (2 , 4)** | **0.2 (0.1 , 0.2)** | **20.5 (-25.9 , 97)** |
| **Lesotho** | **1 (1 , 2)** | **0.1 (0.1 , 0.2)** | **3 (2 , 4)** | **0.2 (0.1 , 0.3)** | **55.6 (-2.5 , 166.1)** |
| **Namibia** | **1 (1 , 1)** | **0.1 (0.1 , 0.2)** | **2 (2 , 3)** | **0.1 (0.1 , 0.2)** | **13.4 (-22.6 , 70.6)** |
| **South Africa** | **33 (27 , 37)** | **0.1 (0.1 , 0.2)** | **64 (52 , 78)** | **0.1 (0.1 , 0.2)** | **-2.8 (-18.2 , 14.3)** |
| **Eswatini** | **0 (0 , 1)** | **0.1 (0.1 , 0.2)** | **1 (1 , 2)** | **0.2 (0.1 , 0.2)** | **21.1 (-22.7 , 94.2)** |
| **Zimbabwe** | **6 (5 , 7)** | **0.1 (0.1 , 0.1)** | **14 (9 , 21)** | **0.2 (0.1 , 0.2)** | **29.9 (-8.1 , 87.6)** |
| **Western Sub-Saharan Africa** | **184 (133 , 293)** | **0.2 (0.1 , 0.3)** | **559 (439 , 740)** | **0.2 (0.2 , 0.3)** | **28.4 (-7.3 , 86.2)** |
| **Benin** | **4 (3 , 7)** | **0.2 (0.1 , 0.3)** | **13 (9 , 19)** | **0.2 (0.1 , 0.3)** | **8.6 (-22.5 , 60.9)** |
| **Burkina Faso** | **9 (5 , 17)** | **0.2 (0.1 , 0.3)** | **25 (16 , 38)** | **0.2 (0.1 , 0.3)** | **12.6 (-18.3 , 57.1)** |
| **Cameroon** | **10 (7 , 16)** | **0.2 (0.1 , 0.3)** | **36 (23 , 59)** | **0.2 (0.1 , 0.4)** | **17.4 (-20.9 , 75.4)** |
| **Cabo Verde** | **0 (0 , 1)** | **0.2 (0.1 , 0.3)** | **1 (1 , 1)** | **0.2 (0.1 , 0.3)** | **34.3 (-3.2 , 110.2)** |
| **Chad** | **6 (3 , 10)** | **0.2 (0.1 , 0.3)** | **15 (10 , 24)** | **0.2 (0.1 , 0.3)** | **14.9 (-17.6 , 71.1)** |
| **CÃ´te d'Ivoire** | **10 (7 , 15)** | **0.2 (0.1 , 0.3)** | **28 (19 , 42)** | **0.2 (0.1 , 0.3)** | **5.3 (-27.1 , 53.6)** |
| **Gambia** | **1 (0 , 1)** | **0.2 (0.1 , 0.3)** | **3 (2 , 4)** | **0.2 (0.2 , 0.4)** | **40.7 (-7.2 , 134.2)** |
| **Ghana** | **30 (23 , 43)** | **0.4 (0.3 , 0.5)** | **122 (70 , 181)** | **0.6 (0.3 , 0.9)** | **57.5 (-17 , 157.7)** |
| **Guinea** | **6 (4 , 11)** | **0.2 (0.1 , 0.3)** | **14 (10 , 21)** | **0.2 (0.2 , 0.3)** | **20.1 (-16 , 75.4)** |
| **Guinea-Bissau** | **1 (1 , 2)** | **0.3 (0.1 , 0.5)** | **3 (2 , 4)** | **0.3 (0.2 , 0.4)** | **8 (-27.5 , 69.1)** |
| **Liberia** | **2 (1 , 3)** | **0.2 (0.1 , 0.3)** | **5 (3 , 7)** | **0.2 (0.1 , 0.3)** | **4.8 (-26.5 , 48.6)** |
| **Mali** | **10 (6 , 20)** | **0.2 (0.1 , 0.4)** | **24 (16 , 37)** | **0.2 (0.1 , 0.4)** | **2.9 (-29.1 , 44.9)** |
| **Mauritania** | **3 (2 , 5)** | **0.3 (0.2 , 0.4)** | **5 (4 , 9)** | **0.2 (0.2 , 0.4)** | **-14.8 (-45 , 28.3)** |
| **Niger** | **7 (3 , 13)** | **0.2 (0.1 , 0.3)** | **20 (11 , 32)** | **0.2 (0.1 , 0.3)** | **6 (-20.1 , 45.5)** |
| **Nigeria** | **71 (49 , 116)** | **0.1 (0.1 , 0.2)** | **205 (139 , 321)** | **0.2 (0.1 , 0.3)** | **30.3 (-12.9 , 105.4)** |
| **Sao Tome and Principe** | **0 (0 , 0)** | **0.1 (0.1 , 0.2)** | **0 (0 , 0)** | **0.2 (0.1 , 0.3)** | **39.4 (-6.7 , 109.4)** |
| **Senegal** | **7 (5 , 12)** | **0.2 (0.1 , 0.3)** | **20 (14 , 30)** | **0.2 (0.2 , 0.3)** | **15.1 (-20.6 , 64.6)** |
| **Sierra Leone** | **3 (2 , 5)** | **0.2 (0.1 , 0.2)** | **9 (6 , 12)** | **0.2 (0.1 , 0.3)** | **21 (-18.5 , 83.8)** |
| **Togo** | **3 (2 , 5)** | **0.2 (0.1 , 0.3)** | **11 (7 , 16)** | **0.2 (0.2 , 0.3)** | **14.1 (-18.7 , 62.7)** |
| **Eastern Sub-Saharan Africa** | **103 (51 , 175)** | **0.1 (0.1 , 0.2)** | **237 (136 , 348)** | **0.1 (0.1 , 0.2)** | **1.4 (-28.1 , 37.1)** |
| **Burundi** | **3 (2 , 5)** | **0.1 (0.1 , 0.2)** | **6 (3 , 9)** | **0.1 (0 , 0.1)** | **-7.5 (-42.9 , 43.9)** |
| **Comoros** | **0 (0 , 0)** | **0.1 (0 , 0.2)** | **1 (0 , 1)** | **0.1 (0.1 , 0.2)** | **14.6 (-25.8 , 143.1)** |
| **Djibouti** | **0 (0 , 0)** | **0.1 (0.1 , 0.2)** | **1 (1 , 2)** | **0.1 (0.1 , 0.2)** | **18.4 (-22 , 89.8)** |
| **Eritrea** | **2 (1 , 4)** | **0.1 (0.1 , 0.2)** | **6 (3 , 9)** | **0.2 (0.1 , 0.2)** | **23.5 (-18.3 , 102.3)** |
| **Ethiopia** | **34 (16 , 65)** | **0.1 (0.1 , 0.2)** | **55 (25 , 84)** | **0.1 (0 , 0.2)** | **-22 (-58.2 , 35.5)** |
| **Kenya** | **8 (5 , 12)** | **0.1 (0 , 0.1)** | **31 (21 , 48)** | **0.1 (0.1 , 0.2)** | **35.6 (6.6 , 92.3)** |
| **Madagascar** | **8 (4 , 13)** | **0.1 (0.1 , 0.2)** | **18 (11 , 28)** | **0.1 (0.1 , 0.2)** | **0.8 (-28 , 42.7)** |
| **Malawi** | **5 (2 , 9)** | **0.1 (0.1 , 0.2)** | **11 (5 , 17)** | **0.1 (0.1 , 0.2)** | **5.4 (-29.9 , 49.8)** |
| **Mozambique** | **9 (3 , 15)** | **0.1 (0 , 0.2)** | **21 (10 , 35)** | **0.2 (0.1 , 0.2)** | **27.8 (-10 , 110.1)** |
| **Rwanda** | **4 (3 , 7)** | **0.1 (0.1 , 0.2)** | **8 (5 , 12)** | **0.1 (0.1 , 0.2)** | **-10 (-49.2 , 49.6)** |
| **Somalia** | **4 (1 , 7)** | **0.1 (0 , 0.2)** | **9 (3 , 17)** | **0.1 (0 , 0.2)** | **2 (-27.9 , 42.8)** |
| **South Sudan** | **3 (1 , 4)** | **0.1 (0 , 0.1)** | **4 (2 , 7)** | **0.1 (0 , 0.1)** | **-0.2 (-30.9 , 42.2)** |
| **United Republic of Tanzania** | **13 (6 , 19)** | **0.1 (0.1 , 0.2)** | **34 (19 , 51)** | **0.1 (0.1 , 0.2)** | **9.7 (-19.7 , 52.5)** |
| **Uganda** | **6 (2 , 9)** | **0.1 (0 , 0.1)** | **17 (9 , 25)** | **0.1 (0.1 , 0.1)** | **25.1 (-10.5 , 107.9)** |
| **Zambia** | **5 (3 , 9)** | **0.1 (0.1 , 0.2)** | **14 (9 , 22)** | **0.1 (0.1 , 0.2)** | **11.7 (-26.2 , 62.1)** |
| **Central Sub-Saharan Africa** | **31 (18 , 52)** | **0.1 (0.1 , 0.2)** | **79 (50 , 123)** | **0.1 (0.1 , 0.2)** | **4 (-23.8 , 44.3)** |
| **Angola** | **6 (3 , 12)** | **0.1 (0.1 , 0.2)** | **20 (13 , 31)** | **0.1 (0.1 , 0.2)** | **7 (-31.4 , 72)** |
| **Central African Republic** | **2 (1 , 4)** | **0.1 (0.1 , 0.2)** | **4 (2 , 7)** | **0.1 (0.1 , 0.2)** | **-4.6 (-30.5 , 30.3)** |
| **Congo** | **2 (1 , 3)** | **0.1 (0.1 , 0.2)** | **4 (3 , 8)** | **0.1 (0.1 , 0.2)** | **-6.2 (-41.5 , 46.5)** |
| **Democratic Republic of the Congo** | **20 (11 , 32)** | **0.1 (0.1 , 0.2)** | **49 (28 , 76)** | **0.1 (0.1 , 0.2)** | **3.8 (-25.6 , 42.2)** |
| **Equatorial Guinea** | **0 (0 , 0)** | **0.1 (0 , 0.2)** | **1 (0 , 1)** | **0.1 (0.1 , 0.2)** | **11.4 (-47.8 , 177.3)** |
| **Gabon** | **1 (1 , 1)** | **0.1 (0.1 , 0.2)** | **2 (1 , 3)** | **0.1 (0.1 , 0.2)** | **6.3 (-32.7 , 63.1)** |
