# Supplementary material for: Global burden of multiple sclerosis and its attributable risk factors, 1990–2019
Source: Front Neurol. 2024 Oct 25;15:1448377. doi: 10.3389/fneur.2024.1448377 (PMC11545682; doi:10.3389/fneur.2024.1448377)
Supplement: Supplementary file 20 [file Table_4.DOC]

| **Table S4: DALYs due to multiple sclerosis in 1990 and 2019 and the percentage change in the age-standardised rates (ASRs) per 100,000, by location**  **(Generated from data available from http://ghdx.healthdata.org/gbd-results-tool)** | | | | | |
| --- | --- | --- | --- | --- | --- |
|  | **1990** | | **2019** | | **Percentage change in ASRs per 100,000** |
|  | **No (95% UI)** | **ASRs per 100,000 (95% UI)** | **No (95% UI)** | **ASRs per 100,000 (95% UI)** |
| **Global** | **726066 (621892 , 867796)** | **16.1 (13.8 , 19.3)** | **1159832 (1001180 , 1381870)** | **14 (12 , 16.6)** | **-13.2 (-20.9 , -6.3)** |
| **High-income North America** | **142094 (116995 , 171098)** | **45.3 (37.3 , 54.5)** | **241678 (195635 , 278601)** | **49.3 (40.2 , 57.4)** | **8.8 (-5.6 , 17.8)** |
| **Canada** | **16206 (13331 , 19103)** | **52.1 (42.9 , 61.4)** | **30269 (22843 , 36169)** | **58.8 (45.2 , 70.7)** | **12.8 (-4.5 , 23.3)** |
| **Greenland** | **16 (12 , 20)** | **28.2 (21.7 , 35.8)** | **20 (15 , 26)** | **29.7 (22.2 , 38.2)** | **5.5 (-13.1 , 25.5)** |
| **United States of America** | **125869 (102949 , 152967)** | **44.6 (36.6 , 54.2)** | **211385 (172946 , 243686)** | **48.2 (39.6 , 56.2)** | **8.1 (-6.5 , 17.9)** |
| **Australasia** | **5483 (4571 , 7033)** | **24.5 (20.4 , 31.4)** | **11116 (9024 , 13401)** | **28.9 (23.3 , 35.3)** | **17.9 (-10.5 , 32.9)** |
| **Australia** | **4528 (3725 , 5981)** | **24.2 (19.9 , 31.8)** | **9639 (7777 , 11738)** | **29.7 (23.9 , 36.4)** | **22.6 (-7.4 , 40.6)** |
| **New Zealand** | **956 (785 , 1145)** | **26.1 (21.4 , 31.3)** | **1477 (1120 , 1737)** | **24.5 (18.8 , 29.1)** | **-6 (-31.3 , 7)** |
| **High-income Asia Pacific** | **12485 (10441 , 16556)** | **6.1 (5.1 , 8.1)** | **15516 (12324 , 20968)** | **5.6 (4.4 , 7.7)** | **-8.9 (-23.3 , 4.7)** |
| **Brunei Darussalam** | **10 (7 , 14)** | **5.3 (3.5 , 7.2)** | **23 (16 , 34)** | **5 (3.4 , 7.4)** | **-5.9 (-22.7 , 16)** |
| **Japan** | **9681 (8000 , 13217)** | **6.1 (5 , 8.2)** | **11012 (8689 , 15306)** | **5.8 (4.6 , 8)** | **-5.1 (-17.3 , 11.4)** |
| **Singapore** | **131 (93 , 150)** | **4.3 (3.1 , 4.9)** | **254 (172 , 310)** | **3.2 (2.1 , 3.9)** | **-26.2 (-45.9 , 9.2)** |
| **Republic of Korea** | **2663 (2183 , 3588)** | **6.5 (5.3 , 8.7)** | **4226 (3403 , 5399)** | **5.5 (4.4 , 6.9)** | **-15.7 (-39.9 , 1)** |
| **Western Europe** | **192347 (162403 , 237882)** | **40.5 (34.1 , 49.9)** | **271039 (222001 , 324346)** | **43.5 (35.8 , 52.7)** | **7.3 (-13.5 , 16)** |
| **Andorra** | **23 (15 , 33)** | **37.2 (24.6 , 53.2)** | **51 (32 , 73)** | **40.4 (25.3 , 57)** | **8.7 (-16.8 , 38.2)** |
| **Austria** | **3250 (2677 , 4133)** | **34 (28 , 43.2)** | **5323 (3984 , 6323)** | **41.2 (31 , 49)** | **21.3 (-14.7 , 40.5)** |
| **Belgium** | **4645 (3709 , 5439)** | **36.6 (29.7 , 42.9)** | **5927 (4681 , 7041)** | **36.7 (29.5 , 44.1)** | **0.4 (-11.5 , 14.8)** |
| **Cyprus** | **147 (117 , 191)** | **18 (14.3 , 23.4)** | **384 (290 , 471)** | **22 (16.6 , 27.1)** | **22.6 (-16.6 , 54.3)** |
| **Denmark** | **4400 (3126 , 5209)** | **67.6 (48.2 , 80.2)** | **5062 (3778 , 6098)** | **61.9 (47.2 , 74.1)** | **-8.4 (-22.5 , 11.2)** |
| **Finland** | **2285 (1903 , 2741)** | **36.5 (30.4 , 43.7)** | **3165 (2372 , 3740)** | **40.1 (30.5 , 47.8)** | **9.8 (-18.1 , 26.2)** |
| **France** | **22817 (18799 , 27338)** | **33.6 (27.6 , 40.3)** | **35071 (27584 , 41807)** | **39.5 (31.2 , 47.5)** | **17.4 (-1.3 , 31.8)** |
| **Germany** | **49760 (41792 , 64894)** | **47.6 (39.9 , 61.3)** | **58888 (48767 , 74117)** | **46.3 (37.8 , 58.8)** | **-2.9 (-23.2 , 9.1)** |
| **Greece** | **2240 (1914 , 2807)** | **17.7 (15 , 21.7)** | **3843 (2698 , 4588)** | **26.2 (18 , 31.5)** | **48 (4.8 , 77.1)** |
| **Iceland** | **140 (115 , 178)** | **53.6 (43.8 , 68.1)** | **208 (162 , 279)** | **47.9 (37.1 , 64.1)** | **-10.7 (-24.9 , 5.3)** |
| **Ireland** | **2080 (1598 , 2483)** | **57.1 (43.9 , 68.3)** | **3489 (2595 , 4254)** | **55.9 (41.6 , 68.6)** | **-2.2 (-16.7 , 12)** |
| **Israel** | **660 (523 , 807)** | **14.4 (11.4 , 17.6)** | **1611 (1148 , 1980)** | **16.4 (11.7 , 20.2)** | **14.2 (-12.8 , 30.4)** |
| **Italy** | **21694 (17513 , 27394)** | **30.3 (24.5 , 37.7)** | **31010 (24611 , 38344)** | **34.8 (27.4 , 43.4)** | **15 (-7.4 , 23.6)** |
| **Luxembourg** | **242 (200 , 326)** | **50.1 (41.3 , 67.3)** | **393 (309 , 527)** | **46.5 (36.8 , 62)** | **-7.2 (-31.9 , 8.7)** |
| **Malta** | **62 (50 , 87)** | **14.6 (11.9 , 20.4)** | **101 (81 , 133)** | **15.9 (12.8 , 21.2)** | **9 (-14 , 24.7)** |
| **Monaco** | **9 (7 , 13)** | **21.4 (16.1 , 29)** | **15 (12 , 18)** | **26.1 (20.6 , 32.2)** | **22.2 (-10.3 , 56.1)** |
| **Netherlands** | **8822 (7263 , 10321)** | **49.5 (40.7 , 58)** | **11538 (9316 , 13667)** | **47.3 (38.4 , 57)** | **-4.5 (-16.2 , 8.3)** |
| **Norway** | **2808 (2128 , 3277)** | **54.9 (41.6 , 64.2)** | **4724 (3279 , 5773)** | **64.5 (45.5 , 79.4)** | **17.4 (-2.7 , 28.8)** |
| **Portugal** | **2362 (1958 , 3176)** | **19.9 (16.5 , 26.4)** | **2752 (2183 , 3934)** | **17.4 (13.8 , 24.5)** | **-12.6 (-25.4 , 10.7)** |
| **San Marino** | **4 (3 , 5)** | **13.3 (10 , 17.5)** | **6 (5 , 8)** | **14.3 (10.2 , 19.1)** | **7.4 (-11.4 , 30.8)** |
| **Spain** | **11270 (8753 , 15419)** | **25.1 (19.6 , 33.8)** | **20264 (15950 , 25983)** | **30.6 (24 , 39)** | **21.8 (-2.3 , 39.8)** |
| **Sweden** | **5874 (4755 , 7080)** | **53.2 (42.8 , 64.6)** | **8309 (6378 , 10111)** | **58.5 (45.5 , 71.2)** | **9.8 (-7.3 , 19.1)** |
| **Switzerland** | **4481 (3671 , 5585)** | **50.9 (42 , 63.8)** | **6035 (4924 , 7382)** | **46.7 (37.9 , 58.3)** | **-8.3 (-26.5 , 8.5)** |
| **United Kingdom** | **42114 (35892 , 55177)** | **59.2 (50.4 , 76.5)** | **62633 (53152 , 75111)** | **67.5 (57.2 , 82.3)** | **13.9 (-10.2 , 22.5)** |
| **Southern Latin America** | **7248 (6032 , 9269)** | **15.4 (12.8 , 19.7)** | **10639 (8314 , 15021)** | **14 (11 , 19.8)** | **-8.5 (-18.8 , 12.8)** |
| **Argentina** | **5472 (4546 , 7279)** | **17 (14.1 , 22.7)** | **7728 (5856 , 11571)** | **15.6 (11.8 , 23.3)** | **-8.6 (-21.2 , 15.1)** |
| **Chile** | **1179 (935 , 1500)** | **10 (7.9 , 12.6)** | **2193 (1694 , 2861)** | **10 (7.7 , 13)** | **-0.2 (-16.4 , 16.9)** |
| **Uruguay** | **597 (417 , 713)** | **17.7 (12.3 , 21.2)** | **717 (503 , 864)** | **17.4 (12.1 , 21.1)** | **-1.8 (-10.9 , 10.7)** |
| **Eastern Europe** | **77451 (65376 , 106471)** | **30.5 (25.9 , 41.6)** | **69170 (48217 , 125154)** | **26.2 (18.3 , 47.6)** | **-14.2 (-32.9 , 23.7)** |
| **Belarus** | **2221 (1904 , 2928)** | **19.1 (16.3 , 25.1)** | **2271 (1570 , 3666)** | **18.5 (12.7 , 30.1)** | **-3 (-28.5 , 36.8)** |
| **Estonia** | **622 (385 , 747)** | **34.7 (21.6 , 41.7)** | **420 (273 , 560)** | **25.3 (16.7 , 33.5)** | **-27.2 (-45.5 , 15.2)** |
| **Latvia** | **1203 (768 , 1419)** | **39.3 (25.2 , 46.4)** | **805 (527 , 1045)** | **33.3 (22.2 , 43.5)** | **-15.2 (-33.8 , 24.1)** |
| **Lithuania** | **1471 (957 , 1734)** | **36.1 (23.6 , 42.6)** | **1105 (766 , 1406)** | **30.8 (21.9 , 38.9)** | **-14.6 (-33 , 21.8)** |
| **Republic of Moldova** | **526 (414 , 792)** | **11.4 (9 , 17.2)** | **593 (460 , 850)** | **12.7 (9.9 , 17.8)** | **10.9 (-15 , 32.3)** |
| **Russian Federation** | **47502 (40150 , 63657)** | **28.2 (23.9 , 37.4)** | **41082 (28543 , 72956)** | **22.3 (15.4 , 39.6)** | **-21 (-39.1 , 15.6)** |
| **Ukraine** | **23905 (19303 , 36890)** | **40.1 (32.8 , 60)** | **22893 (14285 , 47512)** | **41.3 (26.1 , 83.4)** | **2.9 (-26.5 , 59.7)** |
| **Central Europe** | **57785 (50041 , 67953)** | **41.5 (35.9 , 48.7)** | **51364 (40325 , 75257)** | **32.7 (25.6 , 48.1)** | **-21.1 (-35.8 , 16.4)** |
| **Albania** | **1404 (1105 , 2013)** | **58.2 (47.8 , 78.1)** | **1483 (952 , 2709)** | **41.6 (25.8 , 78.4)** | **-28.6 (-49.6 , 10.6)** |
| **Bosnia and Herzegovina** | **1177 (997 , 1355)** | **24.5 (20.8 , 28.2)** | **986 (758 , 1401)** | **21.9 (17.1 , 30.3)** | **-10.6 (-29.1 , 22.4)** |
| **Bulgaria** | **3445 (2578 , 5454)** | **31.7 (24.1 , 49.4)** | **3316 (2389 , 5337)** | **33.1 (23.8 , 52.5)** | **4.4 (-14.3 , 26.6)** |
| **Croatia** | **1479 (1178 , 1727)** | **24.6 (19.5 , 28.7)** | **1580 (1021 , 2073)** | **26.5 (17.1 , 34.9)** | **7.6 (-28.2 , 39.1)** |
| **Czechia** | **5367 (3534 , 6238)** | **43.5 (28.8 , 50.6)** | **4138 (3194 , 5440)** | **27.3 (21.4 , 36.5)** | **-37.2 (-52.1 , 9.4)** |
| **Hungary** | **5097 (3832 , 5844)** | **40.2 (30.1 , 46.4)** | **3944 (3127 , 5101)** | **29 (22.8 , 37.8)** | **-27.8 (-42.7 , 6)** |
| **Montenegro** | **252 (205 , 347)** | **38.6 (31.3 , 52.9)** | **308 (231 , 443)** | **38 (28.8 , 54.5)** | **-1.5 (-18.5 , 19.6)** |
| **North Macedonia** | **511 (435 , 635)** | **24.7 (21 , 30.4)** | **771 (601 , 993)** | **27 (21.1 , 34.4)** | **9.3 (-11.1 , 33)** |
| **Poland** | **26038 (22765 , 33830)** | **62 (54.1 , 80.3)** | **23645 (16706 , 40543)** | **45.2 (31.8 , 77.3)** | **-27.1 (-44.2 , 20.3)** |
| **Romania** | **6737 (3836 , 8096)** | **26.1 (14.7 , 31.6)** | **3947 (3090 , 5472)** | **15.2 (11.9 , 20.8)** | **-42 (-58.3 , 18.9)** |
| **Serbia** | **4228 (3517 , 5315)** | **38.5 (32.2 , 47.8)** | **4591 (3656 , 5631)** | **40.7 (32.2 , 50.2)** | **5.7 (-18.4 , 33.3)** |
| **Slovakia** | **1196 (1003 , 1423)** | **21.1 (17.6 , 25.2)** | **1704 (1195 , 2214)** | **22.8 (16.1 , 29.4)** | **7.8 (-26.4 , 41.7)** |
| **Slovenia** | **856 (605 , 1120)** | **36.7 (26 , 48)** | **952 (615 , 1231)** | **31.1 (20.9 , 40)** | **-15.2 (-40.6 , 21)** |
| **Central Asia** | **7574 (6135 , 9191)** | **14.4 (11.7 , 17.5)** | **12221 (9341 , 15781)** | **13.8 (10.7 , 17.6)** | **-3.9 (-13.2 , 10.8)** |
| **Armenia** | **385 (309 , 465)** | **12.3 (10 , 14.9)** | **595 (449 , 738)** | **16 (11.9 , 19.9)** | **29.5 (6 , 49.8)** |
| **Azerbaijan** | **525 (421 , 648)** | **8.8 (7.1 , 10.7)** | **1055 (807 , 1323)** | **9.2 (7.1 , 11.5)** | **5.1 (-8.4 , 19.9)** |
| **Georgia** | **531 (410 , 659)** | **8.8 (6.9 , 10.9)** | **579 (402 , 731)** | **12 (8.4 , 15.2)** | **36.1 (6.2 , 62.3)** |
| **Kazakhstan** | **3355 (2584 , 4398)** | **24 (18.7 , 30.8)** | **4688 (3382 , 6549)** | **24.7 (18 , 34.1)** | **3.2 (-11.4 , 20.4)** |
| **Kyrgyzstan** | **342 (248 , 425)** | **10.1 (7.4 , 12.5)** | **517 (396 , 655)** | **8.9 (6.8 , 11.2)** | **-12.1 (-26.8 , 15)** |
| **Mongolia** | **103 (68 , 156)** | **7.5 (5 , 11.4)** | **307 (224 , 405)** | **9.2 (6.8 , 12.1)** | **22.3 (-5.9 , 57)** |
| **Tajikistan** | **277 (221 , 345)** | **8.2 (6.5 , 10.1)** | **654 (501 , 823)** | **8.7 (6.8 , 10.9)** | **6.4 (-7.8 , 23.3)** |
| **Turkmenistan** | **347 (277 , 429)** | **15.4 (12.5 , 18.6)** | **779 (587 , 1020)** | **16.8 (13 , 21.3)** | **9.3 (-8 , 29)** |
| **Uzbekistan** | **1708 (1278 , 2090)** | **12.8 (9.5 , 15.8)** | **3046 (2222 , 4164)** | **10.2 (7.5 , 14)** | **-20.4 (-36.7 , 20.9)** |
| **Central Latin America** | **7920 (6630 , 11288)** | **7 (5.8 , 10)** | **24524 (20015 , 29814)** | **9.6 (7.9 , 11.7)** | **38.5 (0.1 , 68.1)** |
| **Colombia** | **1321 (1135 , 1811)** | **5.5 (4.7 , 7.5)** | **3400 (2430 , 4560)** | **6.5 (4.7 , 8.7)** | **18.8 (-24.4 , 55.7)** |
| **Costa Rica** | **158 (137 , 196)** | **7.2 (6.2 , 9)** | **445 (345 , 563)** | **8.5 (6.6 , 10.7)** | **18.1 (-7.5 , 47.6)** |
| **El Salvador** | **170 (141 , 197)** | **4.8 (3.9 , 5.5)** | **332 (243 , 430)** | **5.6 (4.1 , 7.2)** | **16.3 (-9.8 , 45.6)** |
| **Guatemala** | **275 (227 , 358)** | **5.5 (4.6 , 7.1)** | **998 (733 , 1270)** | **7.2 (5.2 , 9.1)** | **30.1 (-11.8 , 68.6)** |
| **Honduras** | **171 (136 , 207)** | **6 (4.8 , 7.1)** | **645 (371 , 1092)** | **7.9 (5.1 , 12.4)** | **33.3 (-8.9 , 102.6)** |
| **Mexico** | **4681 (3714 , 7189)** | **8 (6.2 , 12.6)** | **15440 (12665 , 18906)** | **12 (9.8 , 14.7)** | **50.2 (3.3 , 91)** |
| **Nicaragua** | **118 (98 , 142)** | **5.4 (4.5 , 6.5)** | **349 (273 , 431)** | **6.3 (5 , 7.8)** | **16.2 (-6.3 , 46.3)** |
| **Panama** | **105 (76 , 123)** | **5.8 (4.2 , 6.7)** | **252 (181 , 326)** | **6 (4.3 , 7.7)** | **3.7 (-19.6 , 33.4)** |
| **Venezuela (Bolivarian Republic of)** | **920 (763 , 1125)** | **7 (5.8 , 8.5)** | **2664 (1968 , 3508)** | **8.7 (6.4 , 11.4)** | **24.8 (-5 , 62.2)** |
| **Andean Latin America** | **1494 (1246 , 1837)** | **5.7 (4.8 , 7)** | **3735 (2952 , 4623)** | **6.1 (4.9 , 7.6)** | **7.2 (-17 , 34)** |
| **Bolivia (Plurinational State of)** | **335 (249 , 463)** | **8 (6 , 11)** | **888 (670 , 1151)** | **8.7 (6.7 , 11.3)** | **8.3 (-21.2 , 48.5)** |
| **Ecuador** | **361 (311 , 424)** | **5.2 (4.5 , 6.2)** | **980 (764 , 1232)** | **5.9 (4.6 , 7.4)** | **13.1 (-10 , 41.2)** |
| **Peru** | **799 (647 , 985)** | **5.3 (4.3 , 6.5)** | **1866 (1409 , 2476)** | **5.5 (4.1 , 7.3)** | **3.2 (-24.6 , 39.9)** |
| **Caribbean** | **2979 (2564 , 3605)** | **10 (8.7 , 12.1)** | **5730 (4627 , 7078)** | **11.3 (9.1 , 14)** | **12.6 (-8.1 , 30.3)** |
| **Antigua and Barbuda** | **8 (6 , 10)** | **14.3 (12 , 19.2)** | **18 (14 , 23)** | **16.9 (12.7 , 20.8)** | **18.6 (-14.3 , 48.7)** |
| **Barbados** | **40 (34 , 54)** | **15.9 (13.6 , 21.7)** | **79 (60 , 97)** | **20.1 (15.2 , 25)** | **26.5 (-8 , 63)** |
| **Belize** | **7 (5 , 9)** | **5.7 (4.7 , 7.7)** | **27 (22 , 37)** | **7.4 (6 , 10.1)** | **29.5 (7.7 , 63.8)** |
| **Bermuda** | **8 (7 , 11)** | **12.3 (10.5 , 15.5)** | **10 (8 , 14)** | **11.2 (8.6 , 15)** | **-8.9 (-33.6 , 13.4)** |
| **Bahamas** | **27 (23 , 36)** | **12.6 (10.7 , 16.8)** | **65 (50 , 81)** | **14.8 (11.5 , 18.5)** | **17.8 (-12 , 51.1)** |
| **Cuba** | **1297 (1099 , 1594)** | **12 (10.2 , 14.8)** | **2098 (1502 , 2642)** | **13.6 (9.7 , 17.1)** | **13 (-19.6 , 38.3)** |
| **Dominica** | **4 (4 , 5)** | **6.9 (5.8 , 8.6)** | **6 (5 , 8)** | **7.5 (5.9 , 9.5)** | **8.2 (-12.6 , 33.4)** |
| **Dominican Republic** | **294 (242 , 357)** | **5.9 (4.8 , 6.9)** | **799 (590 , 1078)** | **7.6 (5.6 , 10.3)** | **30.2 (-0.3 , 77.4)** |
| **Grenada** | **10 (8 , 13)** | **15.2 (12.8 , 20.5)** | **17 (14 , 24)** | **14.6 (11.9 , 20.2)** | **-3.9 (-19.3 , 26.5)** |
| **Guyana** | **30 (24 , 44)** | **5.5 (4.3 , 7.9)** | **51 (38 , 71)** | **6.7 (5 , 9.3)** | **21.5 (-7.1 , 57.5)** |
| **Haiti** | **519 (358 , 938)** | **11.6 (8.1 , 20.1)** | **1279 (843 , 2199)** | **12.5 (8.3 , 20.9)** | **7.2 (-23.4 , 46)** |
| **Jamaica** | **106 (80 , 126)** | **6 (4.5 , 7.1)** | **212 (164 , 265)** | **7.1 (5.5 , 8.8)** | **17.9 (-3.9 , 45.8)** |
| **Puerto Rico** | **417 (321 , 483)** | **11.6 (9 , 13.5)** | **629 (423 , 827)** | **13.8 (9.2 , 18.2)** | **18.9 (-15.5 , 55.3)** |
| **Saint Kitts and Nevis** | **7 (6 , 9)** | **22.7 (18.4 , 29.3)** | **15 (11 , 21)** | **20.7 (14.3 , 29)** | **-9 (-36.5 , 33.7)** |
| **Saint Lucia** | **8 (7 , 11)** | **8.1 (6.9 , 11.1)** | **19 (16 , 25)** | **9.1 (7.5 , 11.8)** | **12.1 (-10.3 , 33.7)** |
| **Saint Vincent and the Grenadines** | **6 (5 , 8)** | **7.7 (6.5 , 9.9)** | **11 (9 , 14)** | **8.8 (6.9 , 10.6)** | **14.6 (-10.4 , 39.3)** |
| **Suriname** | **19 (16 , 25)** | **6.1 (5 , 8)** | **45 (36 , 59)** | **7.3 (5.8 , 9.4)** | **19.5 (-3.4 , 47.1)** |
| **Trinidad and Tobago** | **62 (51 , 74)** | **6.2 (5.1 , 7.3)** | **138 (86 , 199)** | **8.2 (5 , 11.9)** | **31.7 (-13.2 , 83.6)** |
| **United States Virgin Islands** | **11 (9 , 14)** | **10.4 (8.4 , 13.4)** | **15 (12 , 18)** | **10.4 (8.3 , 13.5)** | **0.9 (-21.9 , 34.5)** |
| **Tropical Latin America** | **10529 (8439 , 13679)** | **9.1 (7.4 , 11.9)** | **25312 (20305 , 31971)** | **10.1 (8.1 , 12.7)** | **10.2 (-8.8 , 20.5)** |
| **Brazil** | **10343 (8280 , 13508)** | **9.2 (7.4 , 12)** | **24795 (19864 , 31459)** | **10.1 (8.1 , 12.8)** | **10 (-9.2 , 20.8)** |
| **Paraguay** | **186 (136 , 237)** | **6.7 (5 , 8.5)** | **517 (350 , 677)** | **8.1 (5.5 , 10.5)** | **20.6 (-1.3 , 48.1)** |
| **East Asia** | **54947 (40126 , 66524)** | **5 (3.6 , 6)** | **75175 (62164 , 95960)** | **3.8 (3.1 , 4.8)** | **-24.4 (-41.9 , 11.1)** |
| **China** | **52674 (38471 , 63630)** | **4.9 (3.6 , 5.9)** | **71439 (58360 , 92254)** | **3.7 (3 , 4.8)** | **-25.2 (-43.2 , 11.2)** |
| **Democratic People's Republic of Korea** | **1426 (907 , 2061)** | **7.1 (4.5 , 10.2)** | **2014 (1447 , 2898)** | **6.2 (4.5 , 8.8)** | **-12.7 (-38.9 , 29.2)** |
| **Taiwan (Province of China)** | **848 (695 , 1033)** | **4.4 (3.6 , 5.4)** | **1722 (1344 , 2318)** | **5.2 (4.1 , 6.9)** | **17.6 (-5.5 , 59.4)** |
| **Southeast Asia** | **18651 (15589 , 24837)** | **5.1 (4.3 , 6.7)** | **32510 (25887 , 44139)** | **4.5 (3.6 , 6.1)** | **-10.9 (-30 , 21.7)** |
| **Cambodia** | **344 (244 , 576)** | **5.4 (3.8 , 8.7)** | **750 (569 , 1065)** | **5 (3.8 , 7.1)** | **-6.5 (-39.4 , 31.5)** |
| **Indonesia** | **5666 (4481 , 7867)** | **4 (3.2 , 5.6)** | **11019 (7647 , 16288)** | **4 (2.8 , 5.9)** | **1 (-24.1 , 32.7)** |
| **Lao People's Democratic Republic** | **177 (116 , 325)** | **6.4 (4.3 , 11.4)** | **316 (232 , 447)** | **5.1 (3.8 , 7.3)** | **-19.6 (-48.8 , 23)** |
| **Malaysia** | **480 (370 , 563)** | **3.7 (2.8 , 4.3)** | **1176 (890 , 1515)** | **3.7 (2.8 , 4.8)** | **0.9 (-22.7 , 37)** |
| **Maldives** | **5 (4 , 7)** | **4 (2.9 , 5.2)** | **15 (8 , 25)** | **2.9 (1.7 , 4.8)** | **-26.9 (-62.6 , 12.3)** |
| **Mauritius** | **44 (37 , 54)** | **4.7 (4 , 5.8)** | **75 (59 , 101)** | **4.5 (3.6 , 6)** | **-3.1 (-21.7 , 23.6)** |
| **Myanmar** | **2094 (1387 , 3854)** | **6.8 (4.5 , 11.8)** | **3083 (2297 , 4460)** | **5.6 (4.2 , 8)** | **-17.3 (-49.1 , 24.5)** |
| **Philippines** | **2475 (2084 , 3425)** | **5.5 (4.5 , 7.9)** | **4861 (3646 , 7364)** | **4.8 (3.6 , 7.4)** | **-11.8 (-29.4 , 20.9)** |
| **Sri Lanka** | **1929 (1088 , 3907)** | **11.1 (7.4 , 20.5)** | **1251 (732 , 2516)** | **5 (2.9 , 9.9)** | **-55.1 (-82.8 , 13.2)** |
| **Seychelles** | **4 (3 , 5)** | **7.6 (5 , 9.3)** | **11 (5 , 16)** | **8.7 (4.4 , 13.1)** | **14.9 (-17.8 , 54.7)** |
| **Thailand** | **2911 (2113 , 4678)** | **5.6 (4.2 , 8.8)** | **3948 (2868 , 5615)** | **4.1 (3 , 5.8)** | **-27.3 (-58.4 , 14.7)** |
| **Timor-Leste** | **19 (13 , 30)** | **3.7 (2.5 , 5.7)** | **38 (25 , 53)** | **4 (2.6 , 5.5)** | **7.5 (-31.2 , 68)** |
| **Viet Nam** | **2477 (1722 , 3482)** | **5.2 (3.6 , 7.5)** | **5924 (4357 , 8295)** | **5.4 (4 , 7.4)** | **2.6 (-27.8 , 49)** |
| **Oceania** | **168 (115 , 226)** | **3.8 (2.6 , 5.2)** | **363 (264 , 502)** | **3.5 (2.5 , 4.8)** | **-9.6 (-26.7 , 14)** |
| **American Samoa** | **1 (1 , 2)** | **4 (3.2 , 4.7)** | **2 (2 , 3)** | **3.7 (3 , 4.7)** | **-6.3 (-26.9 , 24.8)** |
| **Cook Islands** | **1 (1 , 1)** | **5.4 (4.3 , 6.6)** | **1 (1 , 1)** | **4 (3 , 5.2)** | **-25.2 (-44.5 , -0.6)** |
| **Micronesia (Federated States of)** | **4 (3 , 5)** | **5.9 (4.1 , 8.2)** | **5 (3 , 7)** | **5.1 (3 , 7.5)** | **-13.8 (-46.3 , 30.9)** |
| **Fiji** | **26 (20 , 32)** | **4.5 (3.5 , 5.6)** | **38 (29 , 49)** | **4.1 (3.2 , 5.3)** | **-8.7 (-32.8 , 26.2)** |
| **Guam** | **4 (3 , 5)** | **3.5 (2.7 , 4.1)** | **6 (5 , 7)** | **3.1 (2.6 , 3.9)** | **-9.6 (-27.4 , 23)** |
| **Kiribati** | **4 (3 , 7)** | **8.2 (5.9 , 13.2)** | **7 (5 , 11)** | **7.1 (4.8 , 11.7)** | **-13.6 (-34.1 , 11.9)** |
| **Marshall Islands** | **1 (1 , 2)** | **5.7 (3.8 , 7.5)** | **3 (2 , 4)** | **5.3 (3.9 , 7.5)** | **-7.1 (-32.4 , 44.2)** |
| **Nauru** | **0 (0 , 1)** | **5.6 (4.1 , 7.3)** | **0 (0 , 1)** | **4.8 (3.3 , 6.8)** | **-13.3 (-32.1 , 17.5)** |
| **Niue** | **0 (0 , 0)** | **6.4 (4.8 , 8.2)** | **0 (0 , 0)** | **5 (3.6 , 6.9)** | **-21.4 (-45.5 , 15.6)** |
| **Northern Mariana Islands** | **2 (1 , 2)** | **4.8 (3.8 , 5.9)** | **2 (2 , 3)** | **4.2 (3.3 , 5.2)** | **-11.9 (-32.5 , 12.1)** |
| **Palau** | **1 (0 , 1)** | **4.4 (3.3 , 6)** | **1 (1 , 1)** | **4.2 (3.2 , 5.6)** | **-4.6 (-31.8 , 28.6)** |
| **Papua New Guinea** | **89 (52 , 127)** | **3.2 (1.9 , 4.7)** | **230 (148 , 333)** | **3.1 (2 , 4.5)** | **-5.1 (-28.7 , 27.9)** |
| **Samoa** | **6 (4 , 8)** | **5.4 (3.7 , 7.1)** | **8 (6 , 11)** | **4.5 (3.4 , 6)** | **-16.5 (-40.1 , 17.5)** |
| **Solomon Islands** | **12 (7 , 21)** | **6.2 (3.7 , 10.4)** | **29 (17 , 49)** | **5.8 (3.7 , 9.9)** | **-5.8 (-30.7 , 34)** |
| **Tokelau** | **0 (0 , 0)** | **5.1 (3.6 , 7.1)** | **0 (0 , 0)** | **4.1 (2.9 , 5.7)** | **-20.7 (-46.8 , 22.6)** |
| **Tonga** | **3 (2 , 4)** | **4.5 (3.1 , 5.8)** | **4 (3 , 5)** | **4.3 (3.1 , 5.7)** | **-5.6 (-28.6 , 28.9)** |
| **Tuvalu** | **0 (0 , 1)** | **5.6 (4.1 , 7.7)** | **0 (0 , 1)** | **4.5 (3.4 , 6.1)** | **-20.4 (-43.4 , 18.2)** |
| **Vanuatu** | **5 (3 , 7)** | **5 (3.1 , 7.5)** | **11 (8 , 16)** | **5.1 (3.5 , 7.3)** | **2.3 (-25.8 , 49)** |
| **North Africa and Middle East** | **47116 (36128 , 60785)** | **18.8 (14.7 , 23.2)** | **115886 (93053 , 144758)** | **19.9 (16.1 , 24.7)** | **6.2 (-11.6 , 30.5)** |
| **Afghanistan** | **1947 (1124 , 3952)** | **24.5 (14.5 , 48.8)** | **6575 (3900 , 12354)** | **27.1 (16.8 , 48.7)** | **10.9 (-21.5 , 51.8)** |
| **Algeria** | **3057 (2110 , 4340)** | **17.9 (12.9 , 24.4)** | **8523 (5733 , 11226)** | **20.1 (13.6 , 26.4)** | **12.2 (-14.2 , 41.1)** |
| **Bahrain** | **43 (32 , 55)** | **11.1 (8.7 , 14)** | **232 (166 , 309)** | **12.5 (9.1 , 16.2)** | **12.3 (-7.6 , 32.6)** |
| **Egypt** | **7332 (4673 , 13908)** | **14.4 (10.4 , 22.4)** | **14335 (8608 , 35878)** | **15.3 (9.6 , 32.4)** | **6.4 (-44.2 , 137.3)** |
| **Iran (Islamic Republic of)** | **11056 (8407 , 14846)** | **29 (21.9 , 40.2)** | **26915 (21772 , 34918)** | **29.2 (23.7 , 37.4)** | **0.5 (-18.6 , 20.3)** |
| **Iraq** | **1382 (1044 , 1799)** | **12.7 (9.6 , 16.5)** | **4893 (3596 , 6482)** | **14.3 (10.6 , 18.6)** | **12.4 (-8.4 , 39.2)** |
| **Jordan** | **448 (336 , 569)** | **20.4 (15.5 , 25.8)** | **1903 (1351 , 2405)** | **19 (13.6 , 23.9)** | **-6.9 (-24.9 , 11.7)** |
| **Kuwait** | **174 (130 , 225)** | **12.5 (9.7 , 15.9)** | **894 (644 , 1180)** | **17.3 (12.8 , 22.6)** | **38.2 (21.1 , 58.7)** |
| **Lebanon** | **515 (363 , 657)** | **19.5 (13.9 , 24.7)** | **1270 (891 , 1630)** | **23.5 (16.4 , 30.3)** | **20.9 (-2.5 , 51.3)** |
| **Libya** | **440 (317 , 577)** | **16.2 (11.9 , 20.6)** | **1604 (1091 , 2241)** | **21.8 (14.9 , 30.2)** | **34.2 (2.3 , 87.9)** |
| **Morocco** | **2992 (2097 , 4065)** | **16.1 (11.4 , 21.5)** | **7437 (5171 , 10001)** | **20.2 (14.1 , 27)** | **25.1 (-1.6 , 63.3)** |
| **Palestine** | **214 (155 , 285)** | **18 (13.2 , 23.4)** | **726 (572 , 901)** | **19.5 (15.3 , 24.3)** | **8.5 (-18.1 , 44.4)** |
| **Oman** | **182 (127 , 245)** | **14.2 (10.2 , 18.5)** | **728 (501 , 973)** | **17.1 (11.6 , 23.2)** | **20.3 (-15.8 , 68.5)** |
| **Qatar** | **47 (33 , 63)** | **12.6 (9.3 , 16.3)** | **540 (388 , 720)** | **16.8 (12.3 , 21.8)** | **33.7 (10.4 , 61.8)** |
| **Saudi Arabia** | **996 (700 , 1292)** | **9.5 (6.6 , 12.2)** | **4558 (3316 , 5953)** | **11.7 (8.7 , 15)** | **23 (-5 , 77)** |
| **Sudan** | **1521 (977 , 2286)** | **11.7 (7.7 , 16.6)** | **4194 (2566 , 6043)** | **14 (8.9 , 19.2)** | **20.2 (-13.9 , 70.1)** |
| **Syrian Arab Republic** | **986 (723 , 1316)** | **13.1 (9.8 , 17.2)** | **2153 (1605 , 2776)** | **15.2 (11.3 , 19.6)** | **15.4 (-5.9 , 40.2)** |
| **Tunisia** | **1136 (791 , 1512)** | **17.7 (12.5 , 22.8)** | **2938 (2061 , 3853)** | **22.2 (15.5 , 29.2)** | **25.6 (1 , 58.9)** |
| **Turkey** | **11501 (8423 , 17546)** | **23.8 (18.1 , 32.3)** | **19425 (15255 , 24609)** | **21 (16.3 , 27.6)** | **-11.8 (-37.6 , 20)** |
| **United Arab Emirates** | **273 (195 , 358)** | **17.3 (12.2 , 23)** | **2827 (1713 , 4429)** | **20.6 (12.3 , 32.5)** | **19 (-16.5 , 59.7)** |
| **Yemen** | **844 (532 , 1345)** | **11.1 (7.1 , 16.4)** | **3096 (1957 , 4548)** | **14.1 (9.2 , 19.6)** | **26.4 (-9.6 , 72.1)** |
| **South Asia** | **61498 (43488 , 87448)** | **7.8 (5.5 , 10.8)** | **144077 (119712 , 177476)** | **8.6 (7.1 , 10.5)** | **10.3 (-11.7 , 47.5)** |
| **Bangladesh** | **5156 (3289 , 7475)** | **7.8 (5 , 11)** | **11029 (7574 , 14627)** | **7.3 (5 , 9.7)** | **-6.7 (-39.7 , 33.8)** |
| **Bhutan** | **29 (18 , 45)** | **7.8 (4.7 , 11.8)** | **57 (37 , 79)** | **8.2 (5.3 , 11.3)** | **5.4 (-32.6 , 55.5)** |
| **India** | **48814 (35060 , 69271)** | **7.6 (5.4 , 10.6)** | **112992 (92518 , 139835)** | **8.4 (6.9 , 10.4)** | **10.4 (-12.5 , 50.5)** |
| **Nepal** | **1076 (646 , 1777)** | **8.2 (4.9 , 13.1)** | **2338 (1623 , 3146)** | **8.9 (6.1 , 11.9)** | **8.9 (-27.1 , 49.4)** |
| **Pakistan** | **6423 (4303 , 9487)** | **9 (6 , 13.1)** | **17660 (13202 , 25510)** | **10.9 (8.3 , 15.5)** | **22 (-3.2 , 75.4)** |
| **Southern Sub-Saharan Africa** | **2380 (2006 , 2770)** | **6.5 (5.5 , 7.5)** | **4686 (3927 , 5553)** | **6.6 (5.6 , 7.8)** | **1.4 (-9.7 , 12.9)** |
| **Botswana** | **45 (35 , 59)** | **5.9 (4.5 , 7.6)** | **141 (97 , 195)** | **7 (4.9 , 9.7)** | **18.6 (-19.1 , 71.9)** |
| **Lesotho** | **69 (49 , 91)** | **5.8 (4.1 , 7.6)** | **135 (94 , 189)** | **8.1 (5.7 , 11.4)** | **40.6 (-1.6 , 107.4)** |
| **Namibia** | **48 (37 , 59)** | **5.5 (4.3 , 6.8)** | **112 (82 , 149)** | **6.2 (4.6 , 8.2)** | **12.3 (-16.4 , 54.4)** |
| **South Africa** | **1871 (1584 , 2177)** | **6.8 (5.8 , 7.9)** | **3505 (2910 , 4235)** | **6.5 (5.4 , 7.8)** | **-5 (-18.1 , 9.6)** |
| **Eswatini** | **26 (20 , 33)** | **6.1 (4.8 , 7.8)** | **61 (42 , 83)** | **7.4 (5.2 , 10.2)** | **19.6 (-14.9 , 72.2)** |
| **Zimbabwe** | **321 (247 , 389)** | **5.7 (4.5 , 6.8)** | **732 (497 , 1042)** | **7.2 (5 , 10)** | **26.5 (-4.8 , 72.2)** |
| **Western Sub-Saharan Africa** | **9037 (6974 , 13289)** | **7.7 (6 , 11.4)** | **28344 (22716 , 35170)** | **9.9 (8 , 12.5)** | **28 (-4.3 , 70)** |
| **Benin** | **198 (145 , 294)** | **7.7 (5.6 , 11.5)** | **653 (475 , 893)** | **8.6 (6.3 , 12)** | **11.9 (-16.8 , 52.6)** |
| **Burkina Faso** | **438 (279 , 721)** | **8 (5 , 13.2)** | **1285 (890 , 1776)** | **9.2 (6.3 , 13.1)** | **15.5 (-13.5 , 50.4)** |
| **Cameroon** | **482 (362 , 730)** | **7.9 (5.9 , 11.9)** | **1796 (1241 , 2684)** | **9.4 (6.6 , 14.5)** | **19.6 (-16.6 , 69.3)** |
| **Cabo Verde** | **16 (11 , 24)** | **7.5 (5.1 , 11.3)** | **47 (34 , 62)** | **9.1 (6.7 , 11.9)** | **20.8 (-6.8 , 59.7)** |
| **Chad** | **273 (174 , 436)** | **8 (5 , 13)** | **789 (555 , 1114)** | **9.2 (6.5 , 13.3)** | **15.4 (-14.1 , 52)** |
| **CÃ´te d'Ivoire** | **496 (354 , 732)** | **7.4 (5.4 , 10.9)** | **1455 (1033 , 2025)** | **8.1 (5.8 , 11.4)** | **8.8 (-20.5 , 47.7)** |
| **Gambia** | **38 (26 , 58)** | **7.2 (4.9 , 11)** | **141 (104 , 191)** | **10 (7.4 , 14)** | **38.1 (-3.3 , 101.8)** |
| **Ghana** | **1468 (1140 , 2014)** | **16.1 (12.7 , 22)** | **5917 (3770 , 8231)** | **25.2 (16.3 , 34.4)** | **56.6 (-8.7 , 132.9)** |
| **Guinea** | **301 (216 , 491)** | **7.6 (5.4 , 12.4)** | **724 (543 , 992)** | **9.3 (7 , 12.9)** | **22.2 (-11.9 , 62)** |
| **Guinea-Bissau** | **60 (37 , 105)** | **10.6 (6.6 , 18.5)** | **141 (100 , 204)** | **11.7 (8.3 , 17.3)** | **10.3 (-22.8 , 59.9)** |
| **Liberia** | **89 (67 , 131)** | **6.9 (5.2 , 10.3)** | **258 (192 , 346)** | **7.6 (5.7 , 10.4)** | **9.5 (-20.4 , 44.8)** |
| **Mali** | **507 (335 , 871)** | **9.5 (6.2 , 16.3)** | **1256 (881 , 1753)** | **10 (7.1 , 14.3)** | **5.4 (-24.2 , 40.9)** |
| **Mauritania** | **137 (100 , 216)** | **11.1 (8.1 , 17.6)** | **283 (205 , 393)** | **10.3 (7.4 , 14.6)** | **-7.7 (-36.8 , 30)** |
| **Niger** | **354 (218 , 571)** | **8.3 (5 , 13.5)** | **1066 (654 , 1535)** | **9.1 (5.6 , 13.4)** | **8.8 (-16.3 , 40.5)** |
| **Nigeria** | **3508 (2618 , 5110)** | **6.1 (4.6 , 9.1)** | **10530 (7732 , 14645)** | **7.9 (5.7 , 11.2)** | **28.4 (-6.7 , 84.6)** |
| **Sao Tome and Principe** | **4 (3 , 5)** | **5.2 (3.9 , 6.5)** | **10 (7 , 15)** | **6.8 (4.7 , 10.2)** | **30.9 (-5.2 , 86.2)** |
| **Senegal** | **362 (257 , 540)** | **8.5 (6 , 12.6)** | **1018 (745 , 1390)** | **9.9 (7.3 , 13.6)** | **16.5 (-16 , 55.8)** |
| **Sierra Leone** | **152 (107 , 223)** | **6.5 (4.6 , 9.6)** | **441 (325 , 595)** | **8 (6 , 11)** | **24 (-10 , 71.8)** |
| **Togo** | **152 (113 , 229)** | **8 (6 , 12)** | **532 (388 , 723)** | **9.3 (6.8 , 13)** | **16.3 (-14.2 , 55.2)** |
| **Eastern Sub-Saharan Africa** | **5352 (3378 , 8359)** | **5 (3.1 , 7.7)** | **12717 (8611 , 17089)** | **5.1 (3.4 , 6.9)** | **1 (-25 , 27.6)** |
| **Burundi** | **145 (95 , 240)** | **4.6 (2.9 , 7.4)** | **307 (189 , 425)** | **4.3 (2.6 , 6)** | **-6.5 (-38 , 34.7)** |
| **Comoros** | **14 (7 , 21)** | **5 (2.7 , 7.3)** | **34 (23 , 49)** | **5.7 (3.8 , 8)** | **13 (-22.1 , 99)** |
| **Djibouti** | **13 (8 , 18)** | **4.9 (3 , 7)** | **55 (36 , 80)** | **5.6 (3.7 , 8.2)** | **15 (-17.8 , 63.7)** |
| **Eritrea** | **97 (61 , 175)** | **5.9 (3.5 , 10.4)** | **305 (213 , 429)** | **6.9 (4.7 , 9.8)** | **18.1 (-16 , 67.9)** |
| **Ethiopia** | **1707 (989 , 3056)** | **5.9 (3.3 , 10.1)** | **3024 (1797 , 4151)** | **4.8 (2.8 , 6.7)** | **-19.2 (-53.5 , 24.1)** |
| **Kenya** | **447 (312 , 608)** | **3.7 (2.5 , 5.1)** | **1605 (1183 , 2273)** | **4.7 (3.5 , 6.8)** | **27.4 (4.7 , 68.4)** |
| **Madagascar** | **435 (298 , 671)** | **6 (4 , 8.9)** | **1076 (773 , 1490)** | **6 (4.3 , 8.4)** | **-0.2 (-22 , 27.7)** |
| **Malawi** | **291 (175 , 442)** | **5.3 (3.1 , 7.8)** | **606 (377 , 857)** | **5.5 (3.4 , 7.8)** | **4.6 (-25.3 , 37.7)** |
| **Mozambique** | **465 (249 , 723)** | **5.6 (3 , 8.7)** | **1149 (700 , 1736)** | **6.9 (4.1 , 10.2)** | **22.4 (-8.5 , 67)** |
| **Rwanda** | **195 (139 , 351)** | **4.9 (3.5 , 8.4)** | **394 (264 , 551)** | **4.5 (3 , 6.2)** | **-8.7 (-44.6 , 43.7)** |
| **Somalia** | **189 (100 , 341)** | **4.5 (2.3 , 8)** | **511 (264 , 794)** | **4.6 (2.2 , 7.3)** | **2 (-21.8 , 34.2)** |
| **South Sudan** | **134 (83 , 189)** | **4.1 (2.4 , 5.8)** | **240 (154 , 348)** | **4.2 (2.6 , 5.9)** | **2.4 (-22.8 , 36.5)** |
| **United Republic of Tanzania** | **664 (414 , 912)** | **4.5 (2.8 , 6.3)** | **1765 (1151 , 2427)** | **4.9 (3.2 , 6.7)** | **9.1 (-16.6 , 41)** |
| **Uganda** | **295 (165 , 415)** | **3.3 (1.8 , 4.6)** | **893 (580 , 1246)** | **4 (2.6 , 5.7)** | **22.4 (-6.7 , 69.9)** |
| **Zambia** | **257 (184 , 421)** | **6 (4.2 , 9.5)** | **742 (549 , 1086)** | **6.6 (4.9 , 9.7)** | **10.5 (-22.7 , 49.4)** |
| **Central Sub-Saharan Africa** | **1527 (1033 , 2369)** | **4.7 (3.1 , 7.2)** | **4031 (2878 , 5787)** | **4.9 (3.5 , 7.1)** | **4.4 (-20.8 , 36.6)** |
| **Angola** | **337 (217 , 565)** | **5.5 (3.5 , 9.2)** | **1056 (767 , 1498)** | **5.9 (4.2 , 8.3)** | **6.6 (-27.1 , 53)** |
| **Central African Republic** | **93 (60 , 166)** | **5.6 (3.5 , 9.7)** | **185 (123 , 307)** | **5.4 (3.5 , 8.8)** | **-2.9 (-25 , 25.9)** |
| **Congo** | **79 (59 , 129)** | **5.6 (4.2 , 8.9)** | **213 (138 , 369)** | **5.3 (3.5 , 9)** | **-4.7 (-36.8 , 41.3)** |
| **Democratic Republic of the Congo** | **974 (640 , 1450)** | **4.4 (2.8 , 6.5)** | **2464 (1660 , 3501)** | **4.6 (3 , 6.5)** | **3.9 (-21.1 , 34.5)** |
| **Equatorial Guinea** | **12 (7 , 20)** | **4.5 (2.6 , 7.7)** | **38 (23 , 67)** | **4.8 (2.8 , 8.3)** | **7.2 (-41.3 , 123.7)** |
| **Gabon** | **33 (25 , 47)** | **5 (3.8 , 7.1)** | **75 (49 , 125)** | **5.3 (3.5 , 8.8)** | **6.1 (-26.1 , 51.8)** |
